# Identification and Spectroscopic Characterization of Nonheme Iron(III) Hypochlorite Intermediates**

**DOI:** 10.1002/anie.201411995

**Published:** 2015-02-06

**Authors:** Apparao Draksharapu, Davide Angelone, Matthew G Quesne, Sandeep K Padamati, Laura Gómez, Ronald Hage, Miquel Costas, Wesley R Browne, Sam P de Visser

**Affiliations:** Stratingh Institute for Chemistry, Faculty of Mathematics and Natural Sciences, University of Groningen Nijenborgh 4, 9747 AG Groningen (The Netherlands) E-mail: w.r.browne@rug.nl; Manchester Institute of Biotechnology and School of Chemical Engineering and Analytical Science, The University of Manchester 131 Princess Street, Manchester M1 7DN (UK) E-mail: sam.devisser@manchester.ac.uk; Departament de Química and Institute of Computational Chemistry and Catalysis (IQCC), University of Girona Campus de Montilivi, Girona 17071 (Spain); Serveis Tècnics de Recerca (STR), Universitat de Girona Parc Científic i Tecnològic, E-17003 Girona, Spain; Catexel Ltd., BioPartner Center Leiden Galileiweg 8, 2333 BD Leiden (The Netherlands)

**Keywords:** EPR spectroscopy, hypochlorite, iron, metalloenzymes, Raman spectroscopy

## Abstract

Fe^III^–hypohalite complexes have been implicated in a wide range of important enzyme-catalyzed halogenation reactions including the biosynthesis of natural products and antibiotics and post-translational modification of proteins. The absence of spectroscopic data on such species precludes their identification. Herein, we report the generation and spectroscopic characterization of nonheme Fe^III^–hypohalite intermediates of possible relevance to iron halogenases. We show that Fe^III^-OCl polypyridylamine complexes can be sufficiently stable at room temperature to be characterized by UV/Vis absorption, resonance Raman and EPR spectroscopies, and cryo-ESIMS. DFT methods rationalize the pathways to the formation of the Fe^III^-OCl, and ultimately Fe^IV^=O, species and provide indirect evidence for a short-lived Fe^II^-OCl intermediate. The species observed and the pathways involved offer insight into and, importantly, a spectroscopic database for the investigation of iron halogenases.

Halogenation of organic substrates, although relatively rare, is a key process in the biosynthesis of natural products and the post-translational modification of proteins,[1] typically proceeding through the activation of otherwise inert C–H bonds. They have attracted considerable attention because such reactions are of immediate interest and relevance to pharmaceutical development and biotechnology. In nature, halogenation is achieved primarily by (vanadium- and heme-dependent) haloperoxidases and (nonheme Fe^II^-dependent) halogenases, which use H_2_O_2_ and O_2_, respectively.[2, 3] The precise operation of this wide range of enzymes is a matter of continuing interest both for biological reasons and for the potential application of biomimetic complexes in C–H activation and biotechnology. It has been proposed that nonheme iron halogenases react through radical pathways by transferring halide atoms (X^.^), whereas haloperoxidases instead use X^+^ in an electrophilic mechanism.[1c, 3, 4] For example, it has been proposed that in the case of the heme haloperoxidases the reaction proceeds through formation of an Fe^IV^=O species, which reacts with a halide anion to form an Fe^III^-OX complex.[1c] The OCl^−^ ion formed can then react directly or dissociates and is protonated to generate HOX in proximity to substrates. In contrast, nonheme iron-dependent halogenases are expected to generate Fe^IV^=O species and engage in hydrogen abstraction from alkanes followed by attack by an iron-bound chloride atom equivalent.

However, major gaps remain in our understanding of the catalytic mode of action of these enzymes because of the rarity and the lack of spectroscopic data on species formed during their catalytic cycles. The short lifetimes and especially low steady-state concentrations of putative reaction intermediates formed during enzyme-catalyzed halogenation, such as Fe^II^-OCl, Fe^III^-OCl and Fe^IV^=O, present an often insurmountable challenge to their characterization. Thus, biomimetic model complexes provide a valuable alternative to the elucidation of the chemical nature of these intermediates.[5] The intermediacy of somewhat related Fe^III^-O-IPh species, which can undergo homolysis to form Fe^IV^=O species, is, however, precedented in the work of McKenzie and Lennartson,[6a] and Nam and co-workers,[6b,c] whereby the former study included full characterization of the intermediate using crystallography.

Notwithstanding the characterization of a heme iron(III) hypohalite by Fujii et al.[7] and Woggon and Wagenknecht,[8] and the tentative assignment of an intermediate as a nonheme Fe^II^-OCl species by Banse and co-workers[9] (both carried out by UV/Vis absorption spectroscopy in non-aqueous solutions), the absence of spectroscopic data on such species contrasts markedly with the wealth of data assembled regarding heme and nonheme high-valent Fe^IV^=O complexes.[10] This scarcity of spectroscopic data limits our ability to identify the formation of such species under biologically relevant conditions and evaluate their kinetic competence in halogenation reactions.

Herein, a combined experimental and computational study is reported that identifies for the first time the formation of a biomimetic nonheme iron(III) hypohalite complex, that is, [Fe^III^(OX)(MeN4Py)]^2+^ (MeN4Py= 1,1-di(pyridin-2-yl)-*N*,*N*-bis(pyridin-2-ylmethyl)ethanamine; X=Cl or Br). We show that iron(III) hypohalite complexes are formed readily in water at low pH values from [Fe^II^(OH_2_)(MeN4Py)]^2+^ (Scheme 1)[11, 12] upon reaction with NaOCl or NaOBr at room temperature.[13] The Fe^III^–OX species was found to undergo subsequent slower conversion into a relatively stable Fe^IV^=O analogue within several minutes.

**Scheme 1:**
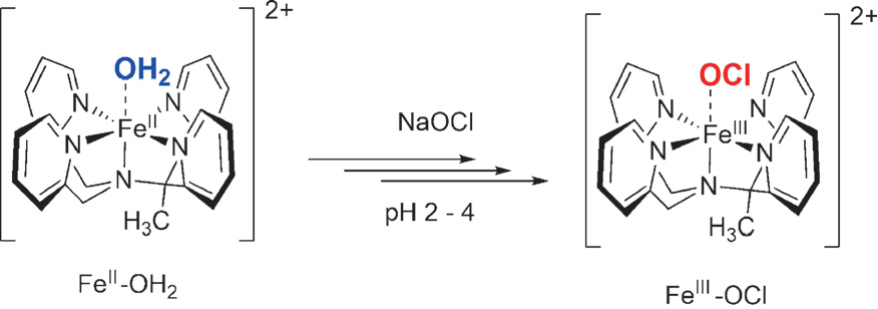
Formation of the complex [Fe^III^(OCl)(MeN4Py)]^2+^ from [Fe^II^(OH_2_)(MeN4Py)]^2+^.

Between pH 1 and 6, the UV/Vis absorption spectrum of [Fe^II^(OH_2_)(MeN4Py)]^2+^ exhibits moderately strong visible absorption bands at circa *λ*=387 and 490 nm (Figure 1 a) that can be attributed to singlet metal-to-ligand charge-transfer transitions (^1^MLCT).[11b] Addition of 0.5 equiv of the two-electron oxidant NaOCl (at pH 2.9) resulted in a near complete loss of absorption in the visible region of the spectrum, with an isosbestic point obtained at approximately *λ*=330 nm within 120 s at room temperature (Figure 1 a). No significant concomitant increase in absorption was detected at longer wavelengths (Figure 1 b). These changes are consistent with an overall one-electron oxidation to form the corresponding Fe^III^-OH complex.[11b,c] Indeed, a weak absorption band at circa *λ*=480 nm remained and the characteristic EPR spectrum of a low-spin Fe^III^ complex was detected (Figure 1 a, inset). This spectrum corresponds to the spectra of [Fe^III^(OH)(MeN4Py)]^2+^.[11c]

**Figure 1:**
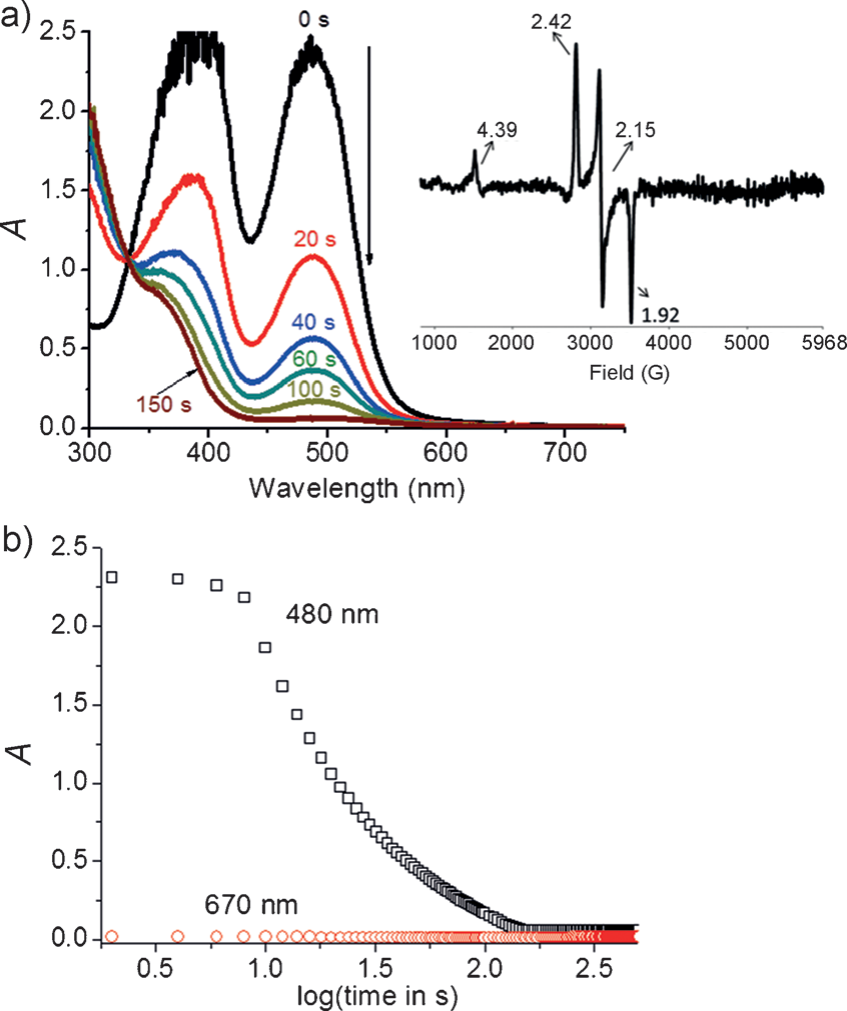
a) UV/Vis absorption spectrum of [Fe^II^(OH_2_)(MeN4Py)]^2+^ (0.5 mm) at pH 2.9 at given time intervals after addition of NaOCl (0.5 equiv) at room temperature. Inset in (a): EPR spectrum obtained from a flash-frozen (to 77 K) sample taken 300 s after addition of NaOCl. b) Change in absorbance at *λ*=480 and 670 nm plotted against log(time).

Addition of a further 0.5 equiv of NaOCl (Figure 2 a) resulted in an initial increase in absorbance at *λ*=480 nm (*ε*>500 m^−1^ cm^−1^).[14] After it had reached a maximum, the absorbance at *λ*=480 nm then decreased concomitant with an increase in absorbance at *λ*=670 nm (Figure 2 b). Similar changes were observed upon addition of 1 or 2 equiv of NaOCl to a solution of [Fe^III^(Cl)(MeN4Py)]^2+^ or [Fe^III^(OMe)(N4Py)]^2+^ (Figure S2–7), both at pH 2.2 and also at pH 3.6. Indeed the only differences observed were in the rates and the extent of the individual processes.

**Figure 2:**
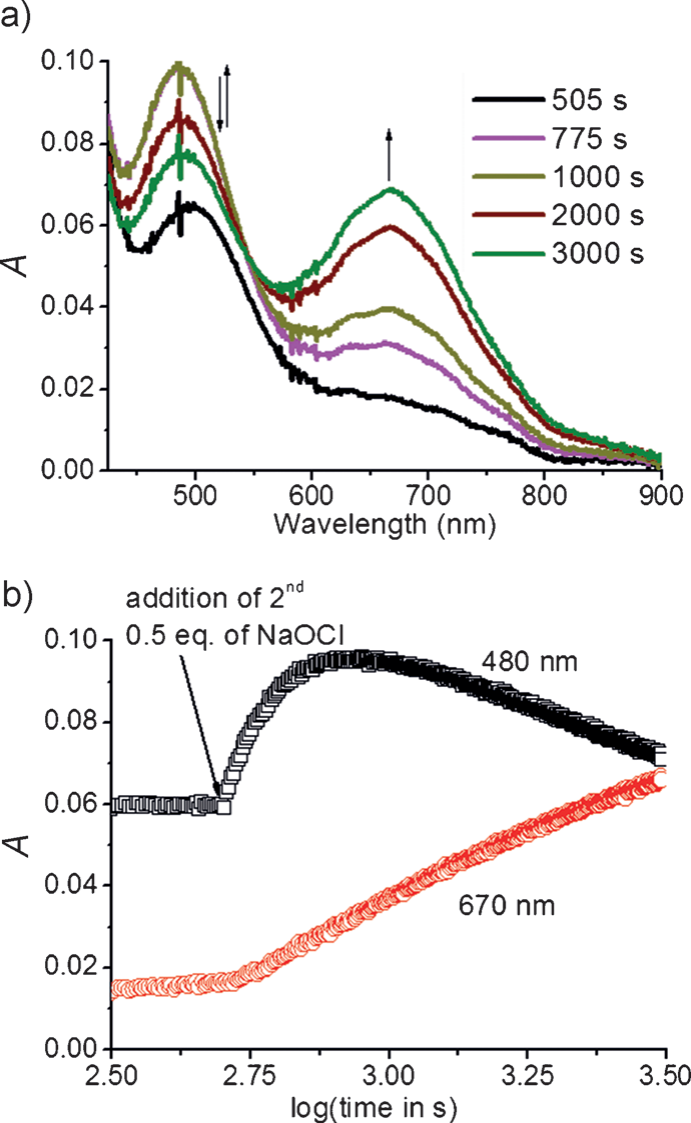
a) UV/Vis absorption spectrum of [Fe^II^(OH_2_)(MeN4Py)]^2+^ (0.5 mm) at pH 2.9 upon addition of a second 0.5 equiv of NaOCl at room temperature. b) Change in absorbance at *λ*=480 and 670 nm plotted against log(time).

The difference in the time dependence of the change in absorbance at *λ*=480 and 670 nm confirms that the two absorption bands correspond to distinct species. The absorbance at 670 nm is attributed to an Fe^IV^=O complex. This assignment was confirmed by comparison of electrospray ionization mass spectrometry (ESIMS), ^1^H NMR (Figure S8) and resonance Raman (rR) spectroscopy (Fe^IV^=O; ν_s_=843 cm^−1^),[15] and other data with an independently prepared sample of [Fe^IV^(O)(MeN4Py)]^2+^. The formation of the Fe^IV^=O species, furthermore, is consistent with the earlier observations by Banse and co-workers of the reaction of related Fe^II^ complexes with NaOCl.[9]

It is apparent from the changes in the UV/Vis absorption spectrum that the intermediate species, which absorbs at *λ*=480 nm, does not form directly upon oxidation of [Fe^II^(OH_2_)(MeN4Py)]^2+^. Instead the intermediate species is formed from a relatively slow ligand exchange between the Fe^III^-OH complex and NaOCl to form [Fe^III^(OCl)(MeN4Py)]^2+^. Thus, in further experiments 2 equiv of NaOCl were employed to achieve a maximum concentration of the Fe^III^-OCl species. The formation of the Fe^III^-OCl species is followed by a slower reaction in which homolysis of the O–Cl bond occurs to yield a relatively stable Fe^IV^=O species. The formation and reactivity of this intermediate Fe^III^-OCl species was studied using a combination of cryo-ESIMS, EPR and rR spectroscopies, and DFT calculations.

Cryo-ESIMS of [Fe^II^(OH_2_)(MeN4Py)]^2+^ (0.5 mm, pH 2.2) recorded after addition of 2 equiv of NaOCl showed ion peaks assigned to [Fe^III^(OH)(MeN4Py)]^2+^ (**C**: *m*/*z* 227.066, 553.080 (+ClO_4_^−^)), [{Fe^III^(OCl)(MeN4Py)}(ClO_4_)]^+^ (**D**: *m*/*z* 587.043), [Fe^IV^(O)(MeN4Py)]^2+^ (**E**: *m*/*z* 226.563, 552.075 (+ClO_4_^−^)). Addition of a further equivalent of NaOCl resulted in an increase in the intensity of the signal at *m*/*z* 552.075 concomitant with a decrease in the intensity of signals attributed to other ions and the complete disappearance of the signal at *m*/*z* 587.043 over time (Figures S9, S10).

EPR spectroscopy (at 77 K) of an aqueous solution of [Fe^II^(OH_2_)(MeN4Py)]^2+^ that was flash frozen 3 min after addition of 2 equiv of NaOCl at room temperature, shows a minor contribution from a high-spin iron(III) species at *g*=4.39 and the expected signals of the low-spin complex [Fe^III^(OH)(MeN4Py)]^2+^ at *g*=2.42, 2.15, and 1.92.[11c] An additional set of signals of similar intensity to those of the low-spin complex were detected at *g*=2.26, 2.15, and 1.97 (Figure 3 a). These values compare well with those reported recently by Cong et al. (*g*=2.256, 2.137, and 1.964) for a putative [Fe^III^(OCl)_2_] heme complex[7] and are characteristic of low-spin iron(III) complexes. The intensity of all signals decreases over time (Figure 3 b) concomitant with the formation of complex [Fe^IV^(O)(MeN4Py)]^2+^, which was confirmed by ^1^H NMR (Figure S8) and rR spectroscopy (Figure 4). Notably, although the Fe^III^-OH and Fe^III^-OCl species are present in approximately equal amounts full conversion into [Fe^IV^(O)(MeN4Py)]^2+^ is observed, suggesting that the Fe^III^-OH and Fe^III^-OCl species are in equilibrium (see below).

**Figure 3:**
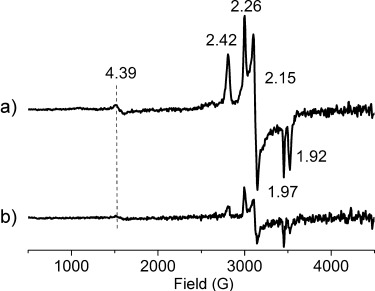
EPR spectroscopy (9.46 GHz) of [Fe^II^(OH_2_)(MeN4Py)]^2+^ flash frozen to 77 K at a) 3 min and b) 10 min after addition of NaOCl (2 equiv).

**Figure 4:**
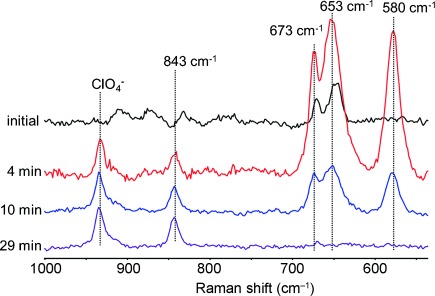
Resonance Raman spectra (*λ*_ex_=473 nm) of [Fe^II^(OH_2_)(MeN4Py)]^2+^ (4 mm) in water at pH 2.2 at selected times after addition of 2 equiv of NaOCl showing the formation of an Fe^III^-OCl complex and subsequently an Fe^IV^=O complex. The band at 934 cm^−1^ is from ClO_4_^−^ because of the acid (HClO_4_) used to adjust the pH value.

Fortuitously, at *λ*_ex_=473 nm the initial [Fe^II^(OH_2_)(MeN4Py)]^2+^,[11b] the intermediate Fe^III^-OCl complex, and the Fe^IV^=O complex formed show resonance enhancement of their Raman scattering and allows the change in concentration of all three species to be followed concurrently by rR spectroscopy (Figure 4). The resonance-enhanced Raman bands of [Fe^II^(OH_2_)(MeN4Py)]^2+^ disappear completely within approximately 1 min of addition of 2 equiv of NaOCl (Figure 4). These bands are replaced in the region below 1000 cm^−1^ by strong bands assigned to the Fe^III^-OCl complex at 580, 653, and 673 cm^−1^ initially. The intensity of these bands subsequently decreases and the characteristic bands of the Fe^IV^=O complex concomitantly appear (at 843 cm^−1^). The time dependence of these spectral changes is consistent with that detected by UV/Vis absorption spectroscopy (Figure S7).

^18^O labelling (i.e. Na^18^OCl in H_2_^18^O; Figures S12 and 13) results in an expected shift of the Fe^IV^=O stretching mode of [Fe^IV^(O)(MeN4Py)]^2+^ from 843 to 807 cm^−1^. The observed shift by 36 cm^−1^ of the band at 843 cm^−1^ is in good agreement with the calculated shift (37 cm^−1^) using the two-atom approximation for an Fe–O stretching vibration. A shift of the bands of [Fe^III^(OCl)(MeN4Py)]^2+^ from 653 and 580 cm^−1^ to 628 and 562 cm^−1^, respectively, was also observed as expected. The band at 653 cm^−1^ (which shifts to 628 cm^−1^) is characteristic of an Fe–O stretching mode and the band at 580 cm^−1^ is typical of an O–Cl stretching mode.[7] The detected shifts of 25 and 18 cm^−1^, respectively, are close to those expected for an Fe–O bond (29 cm^−1^) and O–Cl (23 cm^−1^) modes. Notably, the band at 673 cm^−1^ is unperturbed by the use of Na^18^OCl but undergoes a shift (from 673 to 676 cm^−1^) when NaOBr was used[16] (Figure S15), which is consistent with the assignment of the band as a symmetric Fe-O-Cl bending mode. The resonance Raman spectrum of [Fe^III^(OCl)(MeN4Py)]^2+^ was calculated by DFT methods (Figure S16 and S17). The calculated spectrum shows that although these vibrations involve a larger part of the complex than the Fe-O-Cl core, the assignment of bands to Fe-O-Cl stretching and bending vibrations is, to a first approximation, reasonable.

Taken together, the spectroscopic data confirms the formation of the complex [Fe^III^(OCl)(MeN4Py)]^2+^ and, ultimately, [Fe^IV^(O)(MeN4Py)]^2+^ from [Fe^II^(OH_2_)(MeN4Py)]^2+^. The overall reaction mechanism by which these species form is summarized in Scheme 2. However, although consistent with the data available, the initial step, that is, the reaction between [Fe^II^(OH_2_)(MeN4Py)]^2+^ and OCl^−^, is too fast to detect the intermediate formation of an Fe^IV^=O species. The rate of comproportionation of this Fe^IV^=O species with [Fe^II^(OH_2_)(MeN4Py)]^2+^ is likely to be diffusion limited (Figure S18). DFT calculations were carried out using models and methods benchmarked and calibrated earlier[17] to provide insight into free-energy changes (relative to [Fe^II^(OH_2_)(MeN4Py)]^2+^; labelled ^**5**^**A** in Scheme 2) for each of the proposed steps in the mechanism that leads to the formation of the Fe^III^-OCl (^**6**^**D**) and, ultimately, the Fe^IV^=O (^**3**^**E**) complexes.

**Scheme 2:**
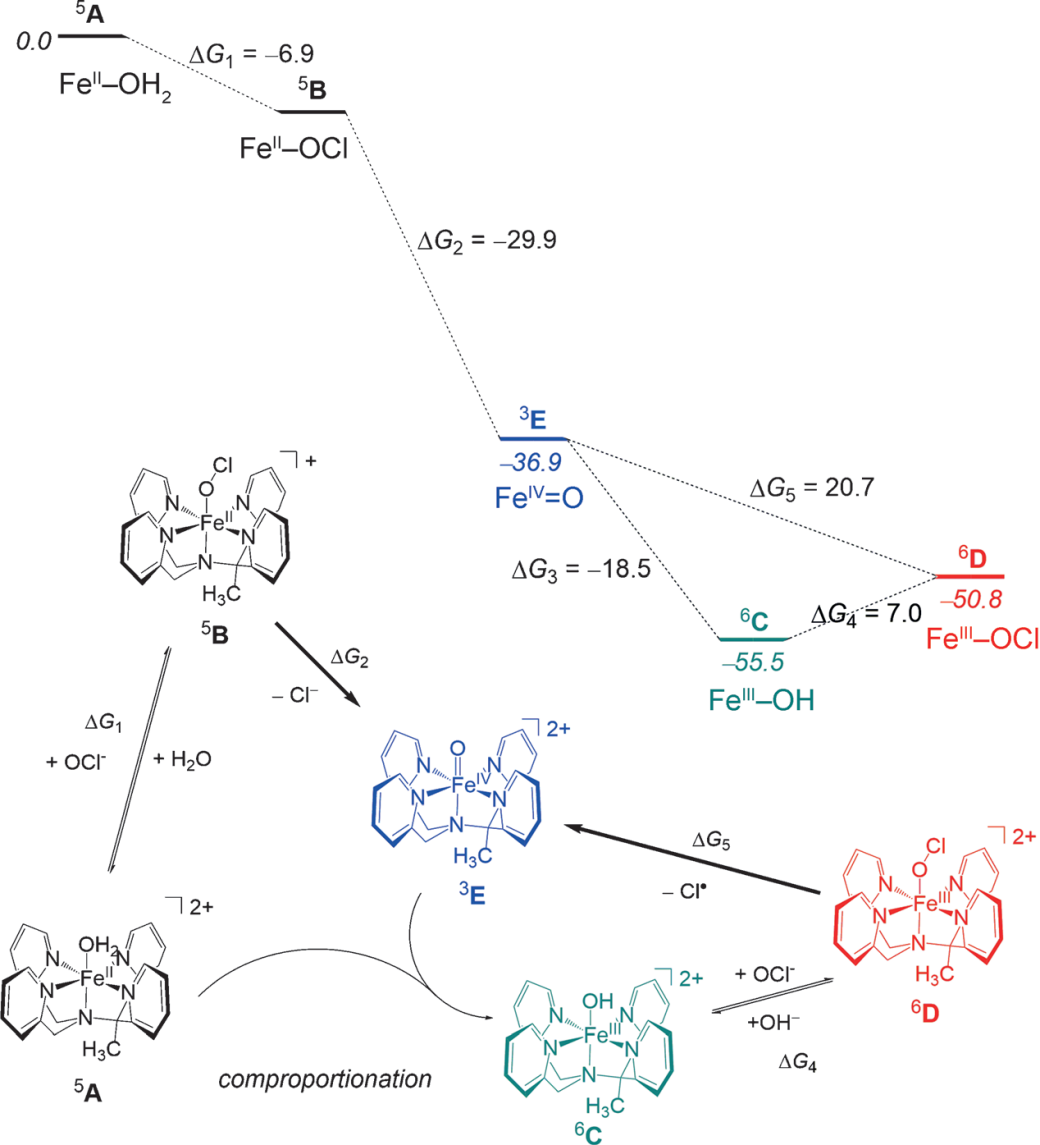
The reaction mechanism for the formation of [Fe^III^(OCl)(MeN4Py)]^2+^ and [Fe^IV^(O)(MeN4Py)]^2+^ with the free energies (Δ*G*) calculated for each step. Data includes dispersion, entropic, and solvent corrections and is given in kcal mol^−1^.

Substitution of the H_2_O ligand bound to [Fe^II^(OH_2_)(MeN4Py)]^2+^ by OCl^−^ to form ^**5**^**B** (Fe^II^-OCl) is exergonic (−6.9 kcal mol^−1^). The subsequent heterolysis of the O-Cl bond to form the Fe^IV^=O complex ^**3**^**E** is exergonic also. Although the activation barrier to this latter step has not been calculated, the reaction is highly exergonic (−29.9 kcal mol^−1^). The exergonicity calculated is consistent with the failure to detect species ^**5**^**B** spectroscopically. The absence of characteristic spectral features of the Fe^IV^=O complex when 0.5 equiv of NaOCl was added to [Fe^II^(OH_2_)(MeN4Py)]^2+^ (Figure 1 a) is expected as only 50 % conversion of [Fe^II^(OH_2_)(MeN4Py)]^2+^ into [Fe^IV^(O)(MeN4Py)]^2+^ can occur. Additionally, the comproportionation of the Fe^II^-OH_2_ and Fe^IV^=O species to form Fe^III^-OH is rapid and essentially complete (Figure S18).

The reaction between the Fe^III^-OH complex ^**6**^**C** and NaOCl was calculated to be endergonic (7.0 kcal mol^−1^), which, together with the endergonicity (20.7 kcal mol^−1^) of the reaction that converts Fe^III^-OCl ^**6**^**D** into the Fe^IV^=O species ^**3**^**E**, is consistent with the observation of a mixture of Fe^III^-OH ^**6**^**C** and Fe^III^-OCl ^**6**^**D** species (Figure 3). Indeed it is the build-up of ^**6**^**D** that allows, for the first time, the observation of a nonheme Fe^III^-OCl species in solution under biologically relevant conditions by rR and EPR spectroscopies and ESIMS (see above).

The absence of detectable amounts of Fe^III^-OCl above pH 5 (i.e. where the concentration of OH^−^ is significant) is in agreement with the conclusion that Fe^III^-OH (^**6**^**C**) and Fe^III^-OCl (^**6**^**D**) are in equilibrium with each other. Indeed, reaction of the Fe^III^-OH complex ^6^**C**, prepared independently, with NaOCl shows that the Fe^III^-OCl species forms rapidly but reaches an equilibrium with Fe^III^-OH and then converts over time into [Fe^IV^(O)(MeN4Py)]^2+^ (Figures S4 and S5).

In conclusion, herein we identify for the first time a nonheme iron(III) hypochlorite complex at room temperature in acidic aqueous media. Importantly, we show that nonheme Fe^IV^=O species form from Fe^III^-OCl complexes by homolytic cleavage of the O-Cl bond and it is likely that they can also form directly from Fe^II^-OCl species. The formation of nonheme Fe^IV^=O species from Fe^III^-OCl is an endergonic reaction whereas their formation from the corresponding Fe^II^-OCl species is highly exergonic. This reactivity is notable as the reverse reaction, that is, the formation of hypohalites by reaction of Fe^IV^=O species with halides, is generally viewed as a key step in the halogenation of organic substrates.[1] In the case of iron halogenases the formation of intermediate Cl-Fe^IV^=O species have been proposed in which the Fe^IV^=O moiety abstracts a hydrogen atom followed by attack of the carbon radical formed on the bound chlorido ligand.[2] Thus, the observation of an Fe^III^-OCl species and of a pathway to a potentially C–H-abstracting Fe^IV^=O that involves concomitant formation of a chloride radical holds implications in regard to our understanding of the mechanisms by which iron haloperoxidases and halogenases operate. Furthermore, although Fe^III^-OCl species can be detected, it is clear that such species are at best transient and may be regarded as spectroscopically analogous to the corresponding Fe^III^-OH and Fe^III^-OOH complexes. The key differences between the UV/Vis absorption, rR, and EPR spectra of these species reported herein provides an important basis of spectral characteristics with which to identify such species if present as components in the biosynthesis of halogenated natural products.

## Supporting Information

As a service to our authors and readers, this journal provides supporting information supplied by the authors. Such materials are peer reviewed and may be re-organized for online delivery, but are not copy-edited or typeset. Technical support issues arising from supporting information (other than missing files) should be addressed to the authors.

miscellaneous_information
