# Supplementary material for: Identification and Spectroscopic Characterization of Nonheme Iron(III) Hypochlorite Intermediates
Source: Angew Chem Int Ed Engl. 2015 Feb 6;54(14):4357–61. doi: 10.1002/anie.201411995 (PMC4670478; doi:10.1002/anie.201411995)
Supplement: Supplementary file 1 — miscellaneous_information [file anie0054-4357-SD1.pdf]

Angewandte Chemie
Eine Zeitschrift der Gesellschaft Deutscher Chemiker

Supporting Information

Identification and Spectroscopic Characterization of Nonheme Iron(III) Hypochlorite Intermediates**

Apparao Draksharapu, Davide Angelone, Matthew G. Quesne, Sandeep K. Padamati, Laura Gómez, Ronald Hage, Miquel Costas, Wesley R. Browne, and Sam P. de Visser**

anie_201411995_sm_miscellaneous_information.pdf

Contents

1. Experimental section ... 2
2. Physical methods ... 2
3. UV/Vis absorption spectroscopy ... 2
4. NMR spectroscopy ... 8
5. ESI-MS ... 9
6. EPR spectroscopy ... 11
7. Raman spectroscopy ... 12
8. Comproportionation between Fe(II)OH_2 and Fe(IV)=O ... 18
9. Computational Details ... 19

1. Experimental section

The ligands 1,1-di(pyridin-2-yl)-N,N-bis(pyridin-2-ylmethyl)methanamine (N4Py) and 1,1 di(pyridin-2-yl)-N,N-bis(pyridin-2-ylmethyl)ethanamine (MeN4Py) and $[(N4Py)Fe^{II}(CH_3CN)](ClO_4)_2$ and $[(N4Py)Fe^{III}(OMe)](ClO_4)_2$ were prepared by literature methods.[1,2] Commercially available chemicals were purchased and used without further purification. $Ca(OCl)_2$ and aqueous NaOCl (10 - 15 %) were purchased from Sigma Aldrich. $Na^{18}OCl$ was prepared by dissolving aqueous NaOCl in $^{18}OH_2$ with 1:7 v/v. Similarly $Ca(^{18}OCl)_2$ was prepared by dissolving solid $Ca(OCl)_2$ in $^{18}OH_2$. All the experiments were carried out at room temperature. Samples were prepared using Milli-Q water and pH was adjusted using dilute aqueous $HClO_4$ or H_2SO_4 and NaOH solutions. Buffers such as phosphate changes the chemistry of these complexes in water, hence, buffers are not employed to control the pH of the reaction mixture in this study.

$[(MeN4Py)Fe^{II}(Cl)](Cl)$. To a solution of MeN4Py (45.2 mg, 0.12 mmol) in methanol (1.5 mL) was added to $FeCl_2{\cdot}4H_2O$ (23.6 mg, 0.12 mmol). After stirring for 2 h, the solution was placed in an ethyl acetate bath and after two days $[(MeN4Py)Fe^{II}(Cl)](Cl)$ (56 mg, 0.11 mmol, 93%) was obtained as orange powder. UV/Vis absorption (in CH_3CN) λ_{max} at 382 nm and 479 nm. ^1H NMR (CD_3CN) δ -10.7, 29.2, 32.1, 35.1, 48.7, 51.8, 53.5, 55.7, 100.6, 145.8 and 166.4. Elem. Anal. % (calc%): C 51.8 (52.1), H 4.72 (5.06), 12.5 (12.7).

2. Physical methods

^1H NMR spectra (400 MHz) were recorded on a Varian Mercury Plus. Chemical shifts are denoted relative to the residual solvent peak (^1H NMR spectra D_2O, 4.79 ppm). Elemental analyses were performed with a Foss-Heraeus CHN Rapid or a EuroVector Euro EA elemental analyzer. UV/Vis absorption spectra were recorded with a HP8453 spectrophotometer or a Specord600 (AnalytikJena) in 10 mm path length quartz cuvettes. EPR spectra (X-band, 9.46 GHz) were recorded on a Bruker ECS106 spectrometer in liquid nitrogen (77 K). Samples for measurement (300 μL) were transferred to EPR tubes, which were frozen in liquid nitrogen immediately. High resolution mass spectra (HRMS) were recorded on a Bruker MicrOTOF-Q II^{TM} Instrumental at Serveis Tècnics of the University of Girona. A cryospray attachment was used for CSI-MS (cryospray mass spectrometry). The temperature of the nebulizing and drying gasses was set at 5 and 0 ºC, respectively. Samples were introduced into the mass spectrometer ion source by direct infusion using a syringe pump and were externally calibrated using sodium formate. The instrument was operated in the positive ion mode. Raman spectra were recorded at λ_{exc} 785 nm using a Perkin Elmer Raman Station at room temperature. Raman spectra at 473 nm (50 mW at source, Cobolt Lasers) were obtained in a 155° backscattering arrangement with Raman scattering collected by a 2.5 cm diameter plano-convex lens (f = 7.5 cm). The collimated Raman scattering passed through an appropriate long pass edge filter (Semrock) and was focused by a second 2.5 cm diameter plano convex lens (f = 10 cm) into a Shamrock300i spectrograph (Andor Technology) with a 1200 L/mm grating blazed at 500 nm and acquired with an DU970N-BV CCD camera (Andor Technology). The spectral slit width was set to 60 μm. Data were recorded and processed using Solis (Andor Technology) with spectral calibration performed using the Raman spectrum of acetonitrile/toluene 50:50 (v:v).[3] Samples were held in quartz 10 mm path length cuvettes. Baseline correction was performed for all spectra.

3. UV/Vis absorption spectroscopy

Direct addition of 1 equiv of NaOCl to $[(MeN4Py)Fe^{II}(Cl)](Cl)$ at pH 2.2 led to a near complete loss in absorbance at 490 nm within 15 s, indicating a complete loss of the Fe(II) complex, after which a new band grew at 480 nm and reached a maximum absorbance within ca. 2 min. Subsequently a decrease but not complete loss in absorbance at 480 nm, concomitant with an increase in absorbance at 670 nm, was observed (Figure S 1). Upon addition of a second equivalent of NaOCl, the band at 480 nm increased

initially with no change in absorbance at 670 nm. After the absorbance at 480 nm reached a maximum, the absorbance at 670 nm began to increase with a concomitant decrease in absorbance at 480 nm. The absorption band in the NIR region (i.e. at 670 nm) is typical of an Fe(IV)=O species.[4] Under acidic conditions the absorption at 670 nm is persistent but disappears rapidly upon an increase in pH (Figure S 1e).

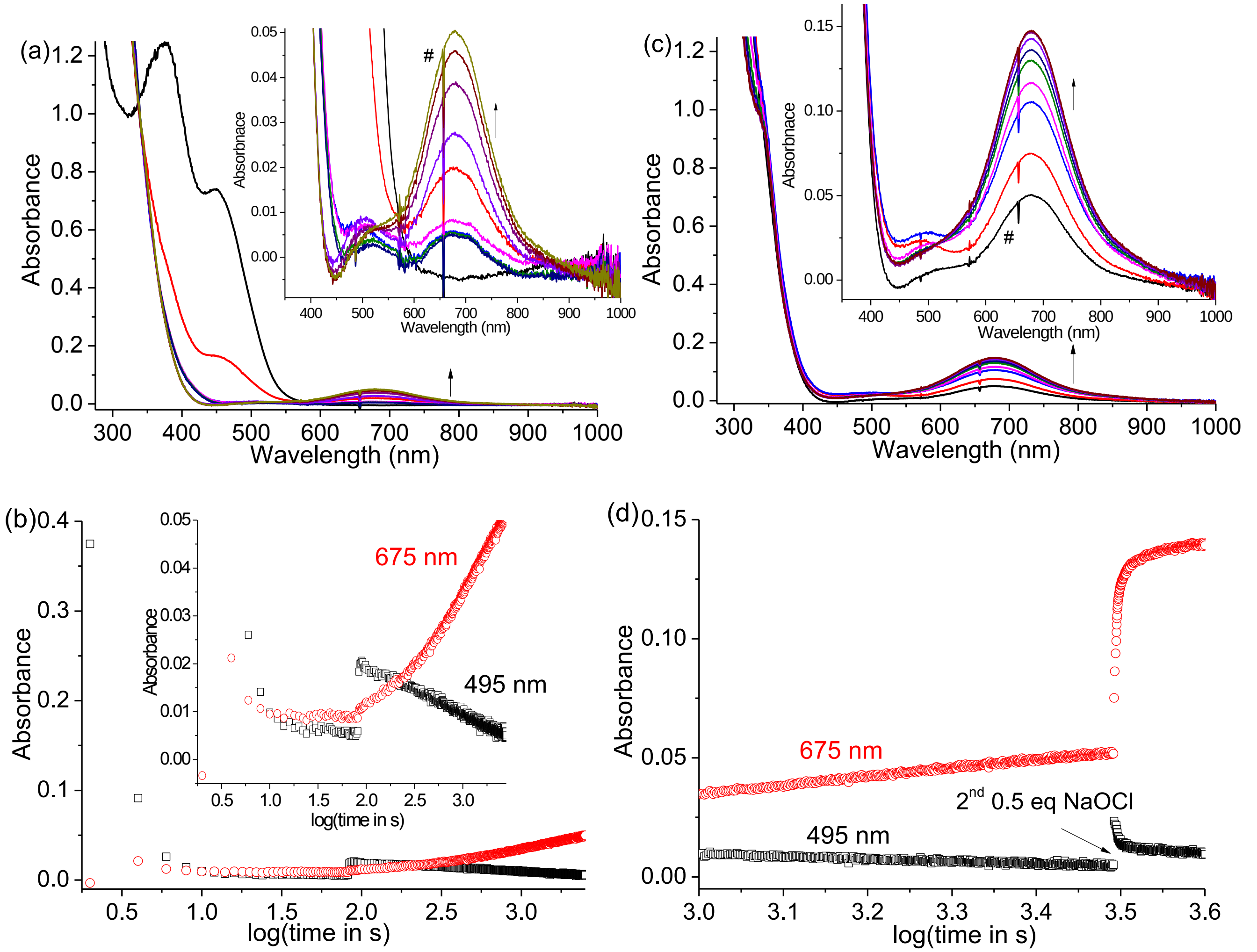

Figure S 1 Changes in UV/Vis absorption (a) after addition of 0.5 equiv of NaOCl to an aqueous solution of $[(N4Py)Fe(CH_3CN)](ClO_4)_2$ (0.5 mM, pH 3.3) and (b) corresponding time dependence of the absorbance at 495 and at 675 nm over time, (c) after addition of a second 0.5 equiv of NaOCl and (d) corresponding time dependence of the absorbance at 495 and at 675 nm overtime. # the sharp spike is instrumental artefact.

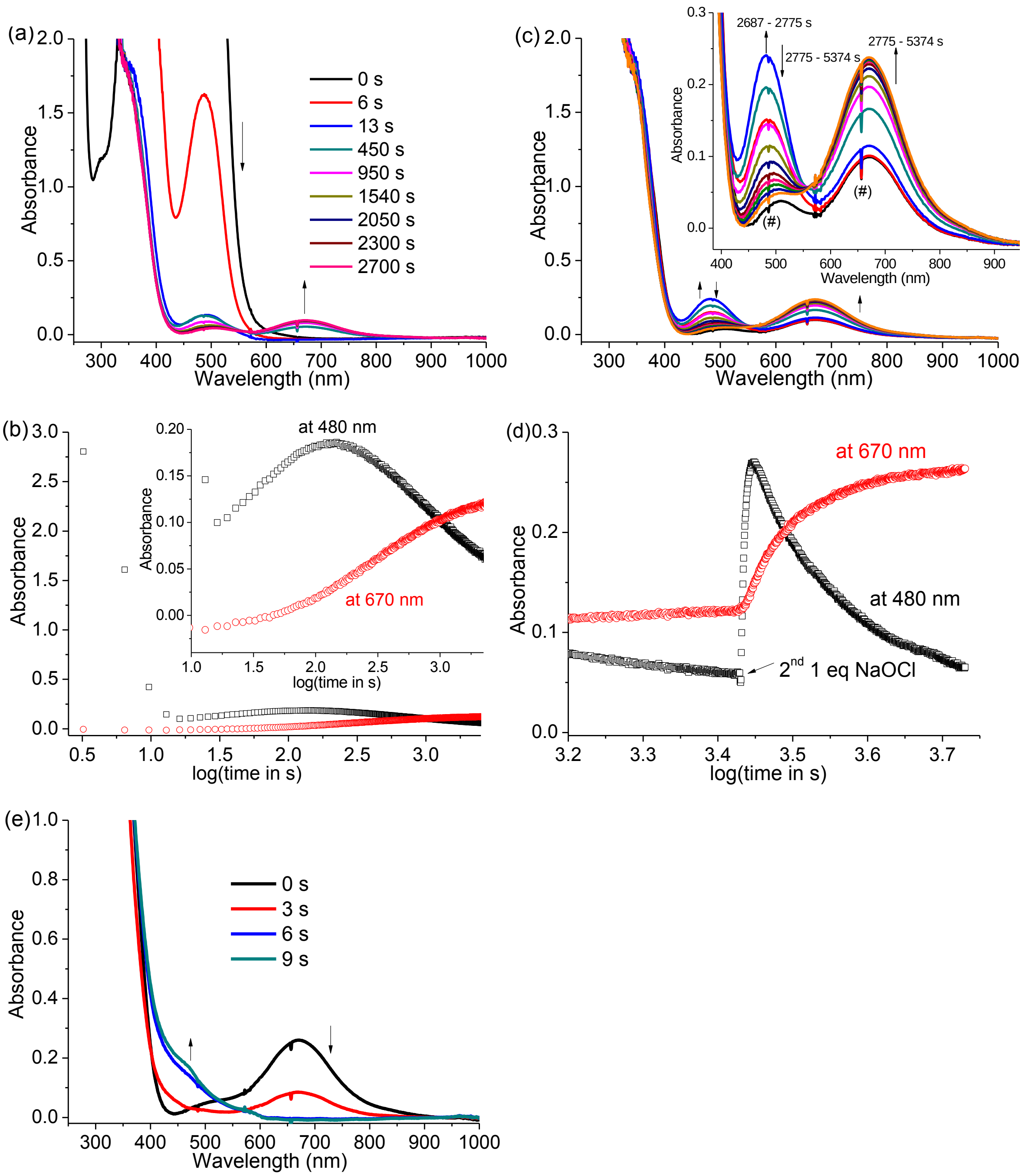

Figure S 2 Changes in UV/Vis absorption (a) after addition of 1 equiv of NaOCl to an aqueous solution of [(MeN4Py)Fe^{II}(Cl)](Cl) (0.5 mM, pH 2.2) and (b) corresponding time dependence of the absorbance at 480 nm and at 670 nm, (c) after addition of a second 1 equiv of NaOCl, (d) corresponding time dependence of the absorbance at 480 nm and at 670 nm and (e) the effect of NaOH on the spectrum of Fe^{IV}=O species. # the sharp spike is an instrumental artefact.

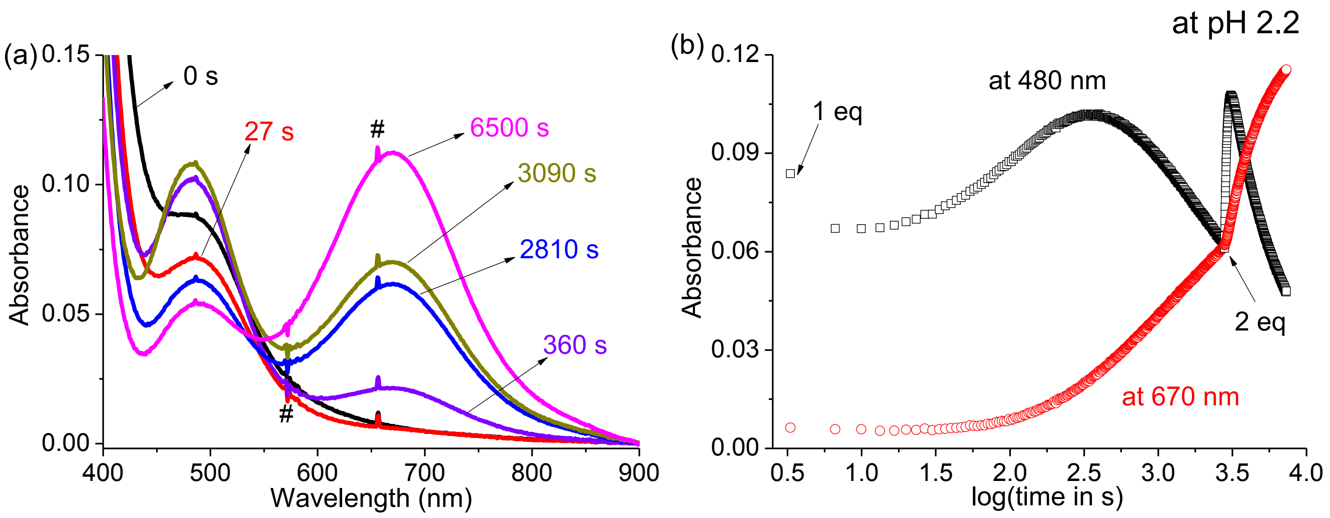

Figure S 3 Changes in UV/Vis absorption (a) after addition of 1 and 2 equiv of NaOCl to an aqueous solution of $[(MeN4Py)Fe^{III}(Cl)](ClO_4)_2$ (0.5 mM, pH 2.2) and (b) corresponding time dependence of the absorbance at 480 and at 670 nm over time. # spike is instrumental artefact.

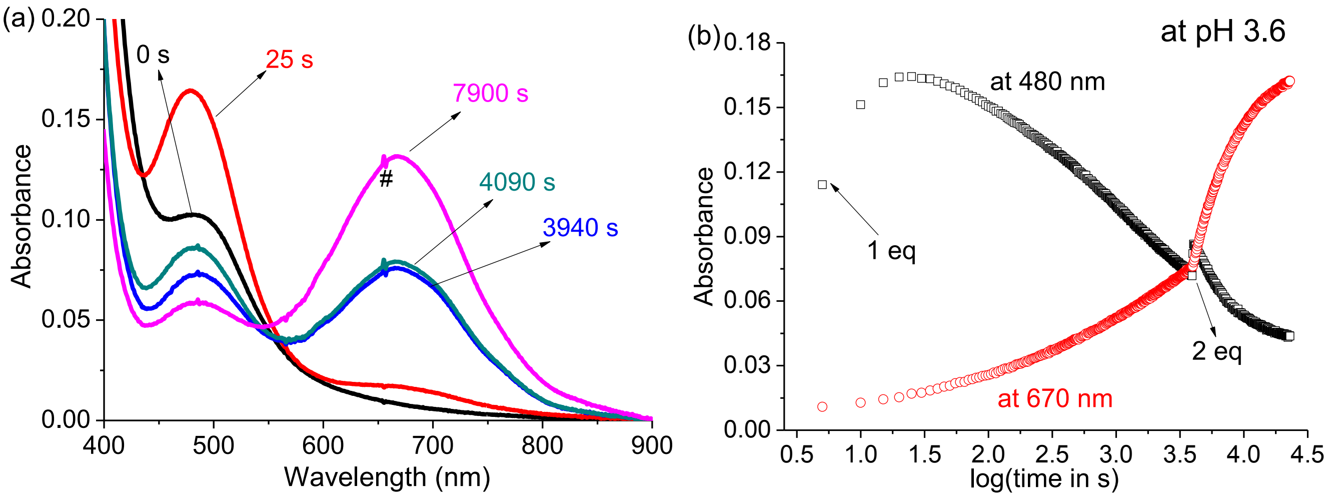

Figure S 4 Changes in UV/Vis absorption (a) after addition of 1 and 2 equiv of NaOCl to an aqueous solution of $[(MeN4Py)Fe^{III}(Cl)](ClO_4)_2$ (0.5 mM, pH 3.6) and (b) corresponding time dependence of the absorbance at 480 and at 670 nm over time. # spike is instrumental artefact.

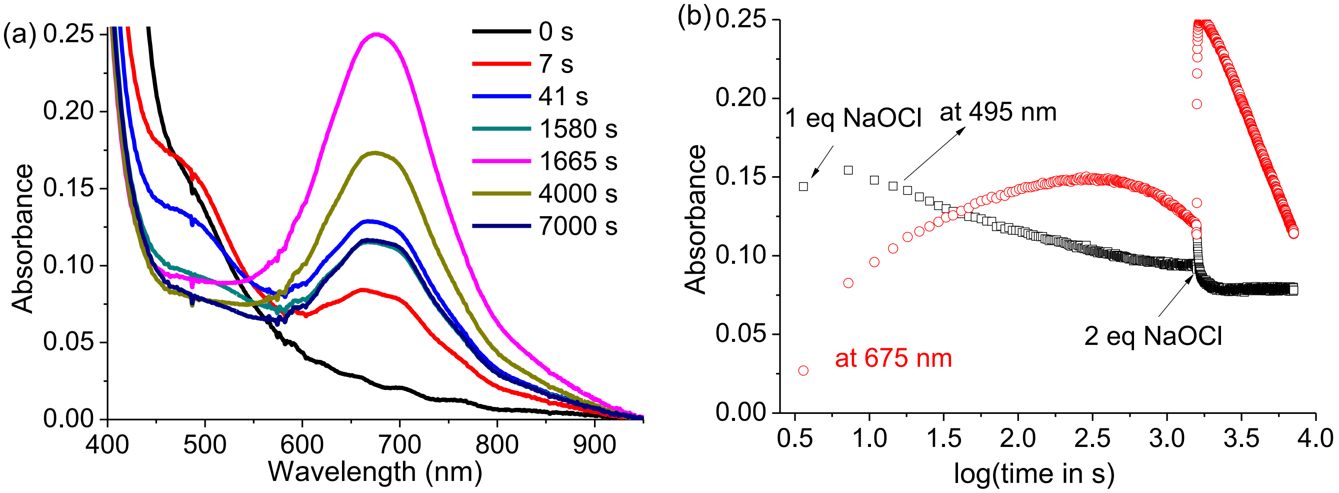

Figure S 5 Changes in UV/Vis absorption (a) after addition of 1 and 2 equiv of NaOCl to an aqueous solution of $[(N4Py)Fe^{III}(OMe)](ClO_4)_2$ (0.5 mM, pH 4.1) and (b) corresponding time dependence of the absorbance at 495 and at 675 nm over time.

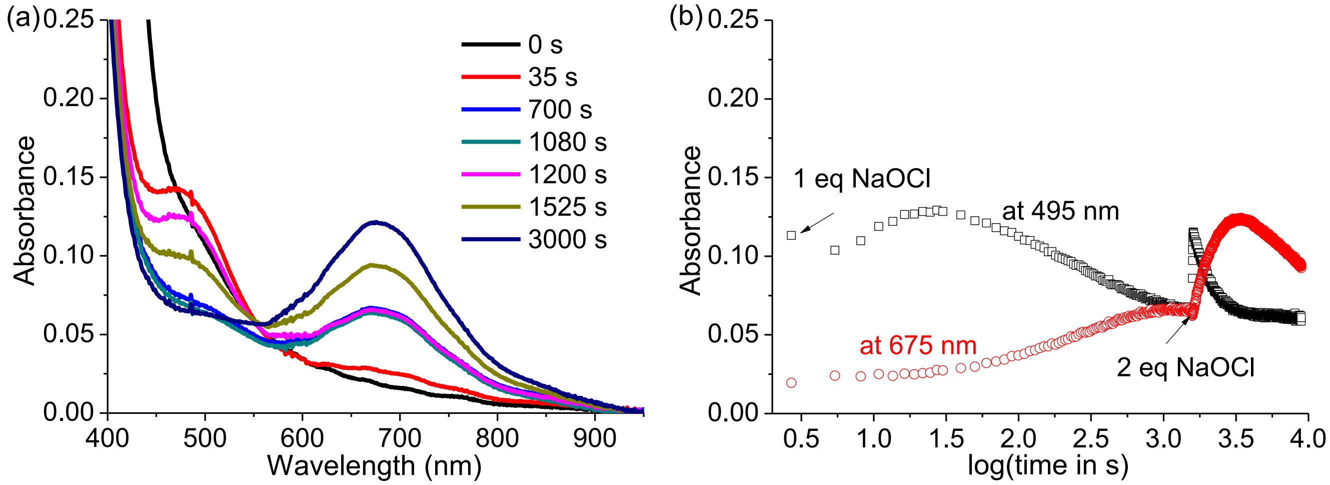

Figure S 6 Changes in UV/Vis absorption (a) after addition of 1 and 2 equiv of NaOCl to an aqueous solution of $[(N4Py)Fe^{III}(OMe)](ClO_4)_2$ (0.5 mM, pH 2.1) and (b) corresponding time dependence of the absorbance at 495 and at 675 nm over time.

Parallel experiments were carried out on the related complex $[(N4Py)Fe^{II}(CH_3CN)](ClO_4)_2$. In contrast to $[(MeN4Py)Fe^{II}(Cl)](Cl)$, addition of 0.5 equiv of NaOCl to an aqueous solution of the complex (at pH = 3.3)[5,6] generates $[(N4Py)Fe^{IV}(O)]^{2+}$ (λ_{max} at 675 nm)[7] immediately with a weaker absorption at 495 nm. Addition of a second 0.5 equiv of NaOCl resulted in a further increase in absorption at 675 nm (i.e. $[(N4Py)Fe^{IV}(O)]^{2+}$, Figure S 2).

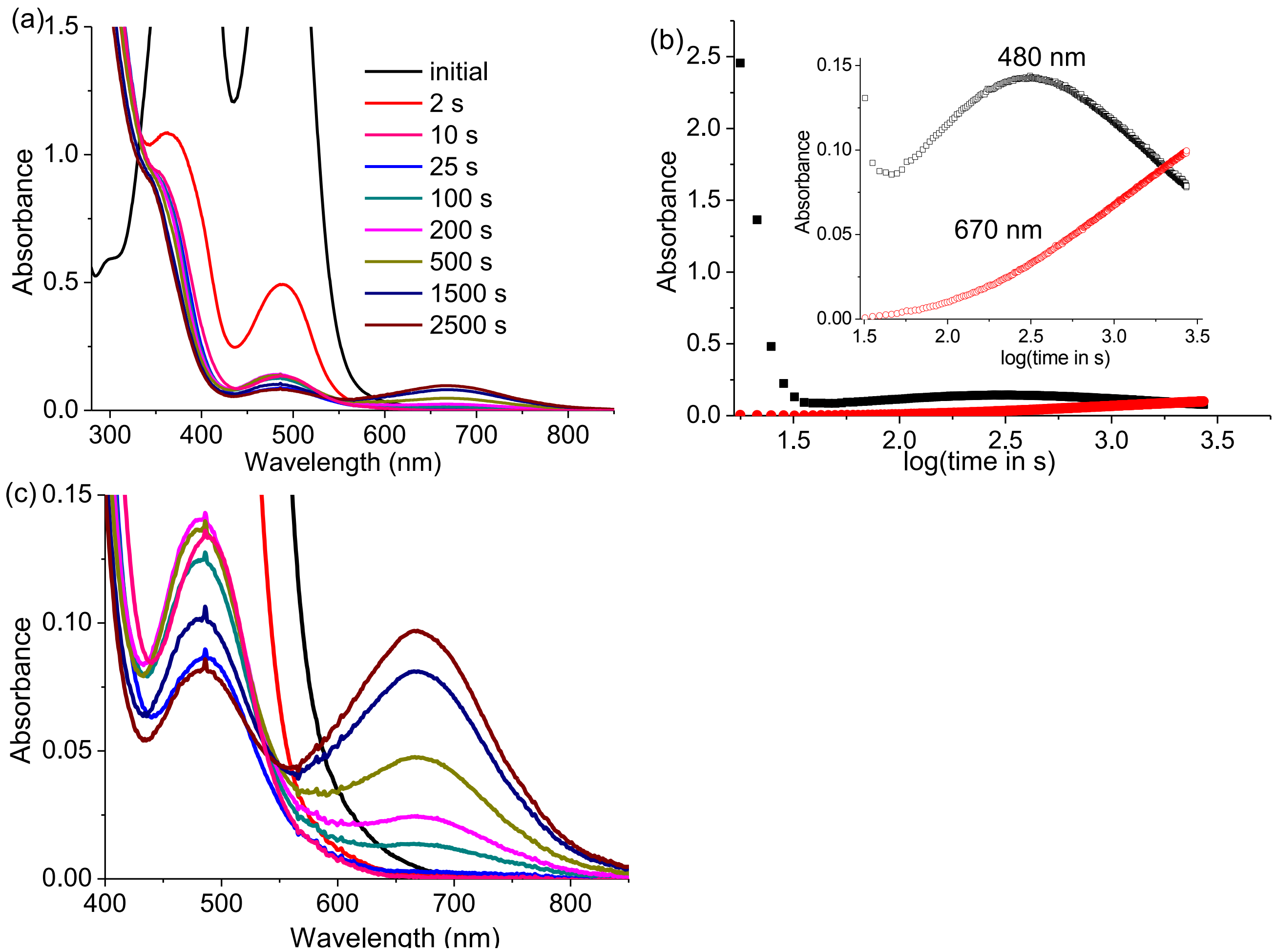

Figure S 7 Changes in UV/Vis absorption (a) after addition of 2 equiv of NaOCl to an aqueous solution of [(MeN4Py)Fe^{II}(Cl)](Cl) (0.5 mM, pH 2.2), (b) corresponding time dependence of the absorbance at 480 nm and at 670 nm, and (c) same as ‘a’ but between 400 to 850 nm.

4. NMR spectroscopy

^1H NMR spectroscopy was employed to characterize the species formed in the reaction of [(MeN4Py)Fe^{II}(Cl)](Cl) with NaOCl in the neutral and low pH. Addition of 0.5 equiv of NaOCl to an aqueous solution of [(MeN4Py)Fe^{II}(Cl)](Cl) (at pH 2.2) resulted in a loss of all signals in the ^1H NMR spectrum. The ^1H NMR spectrum obtained after the appearance of the blue species generated with 2 equiv of NaOCl in water matched that of [(MeN4Py)Fe^{IV}(O)]$^{2+}$generated with CAN/[(MeN4Py)Fe^{II}(Cl)](Cl) in water (Figure S 6).

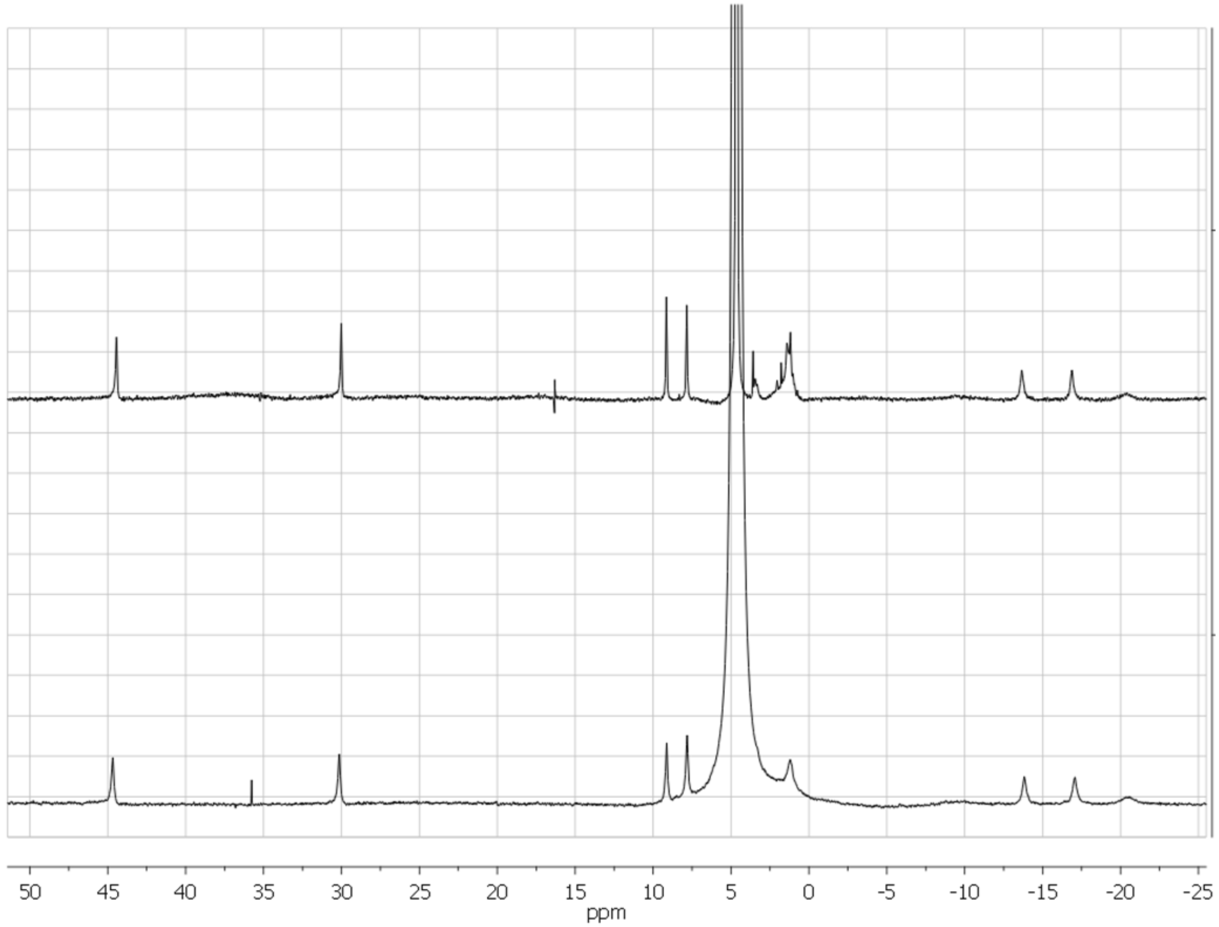

Figure S 8 ^1H NMR spectra of [(MeN4Py)Fe^{IV}(O)]$^{2+}$ generated with CAN from [(MeN4Py)Fe^{II}(Cl)](Cl) in D_2O (top) and blue species generated with [(MeN4Py)Fe^{II}(Cl)](Cl) (5 mM) in D_2O at pH 2.2 (bottom) with 2 equiv of NaOCl.

5. ESI-MS

Addition of 1 equiv of NaOCl to an aqueous solution of [(MeN4Py)Fe^{II}(Cl)]Cl (0.5 mM, pH 2.2), resulted in the appearance of several major ions [(MeN4Py)Fe^{III}(OH)$]^{2+}$ (m/z 227.066), [(MeN4Py)Fe^{IV}(O)$]^{2+}$ (m/z 226.563), [(MeN4Py)Fe^{III}(OCl)(ClO_4)$]^+$ (m/z 587.043), [(MeN4Py)Fe^{III}(OH)(ClO_4)$]^+$ (m/z 553.080), [(MeN4Py)Fe^{IV}(O)(ClO_4)$]^+$ (m/z 552.075) and [(MeN4Py)Fe^{II}(ClO_4)$]^+$ (m/z 536.079). The only ion in the Fe(II) oxidation state observed was [(MeN4Py)Fe^{II}(ClO_4)$]^+$, which decreases in intensity over time. Due to small differences in their m/z values, the ions [(MeN4Py)Fe^{III}(OH)$]^{2+}$ & [(MeN4Py)Fe^{IV}(O)$]^{2+}$ and [(MeN4Py)Fe^{III}(OH)(ClO_4)$]^+$ & [(MeN4Py)Fe^{III}(O)(ClO_4)$]^+$ (m/z 552.075) overlap. Addition of a second equiv of NaOCl increases the intensity of the [(MeN4Py)Fe^{IV}(O)(ClO_4)$]^+$ (m/z 552.075) signal concomitantly with a decrease in intensity of other mono cationic ions. Eventually [(MeN4Py)Fe^{III}(OCl)(ClO_4)$]^+$ is not observed, and only [(MeN4Py)Fe^{IV}(O)(ClO_4)$]^+$ (m/z 552.075) was observed. With $Na^{18}OCl$ instead of $Na^{16}OCl$ all mono cationic ions except for [(MeN4Py)Fe^{II}(ClO_4)$]^+$ (m/z 536.079), were shifted by two m/z units and dicationic ions shifted by one m/z unit. This supports their assignment as [(MeN4Py)Fe^{III}(^{18}OH)$]^{2+}$ (m/z 228.070), [(MeN4Py)Fe^{IV}(^{18}O)$]^{2+}$ (m/z 227.564), [(MeN4Py)Fe^{III}(^{18}OCl)(ClO_4)$]^+$ (m/z 589.047), [(MeN4Py)Fe^{III}(^{18}OH)(ClO_4)$]^+$ (m/z 555.084) and [(MeN4Py)Fe^{IV}(^{18}O)(ClO_4)$]^+$ (m/z 554.078) (Figure S 8).[8]

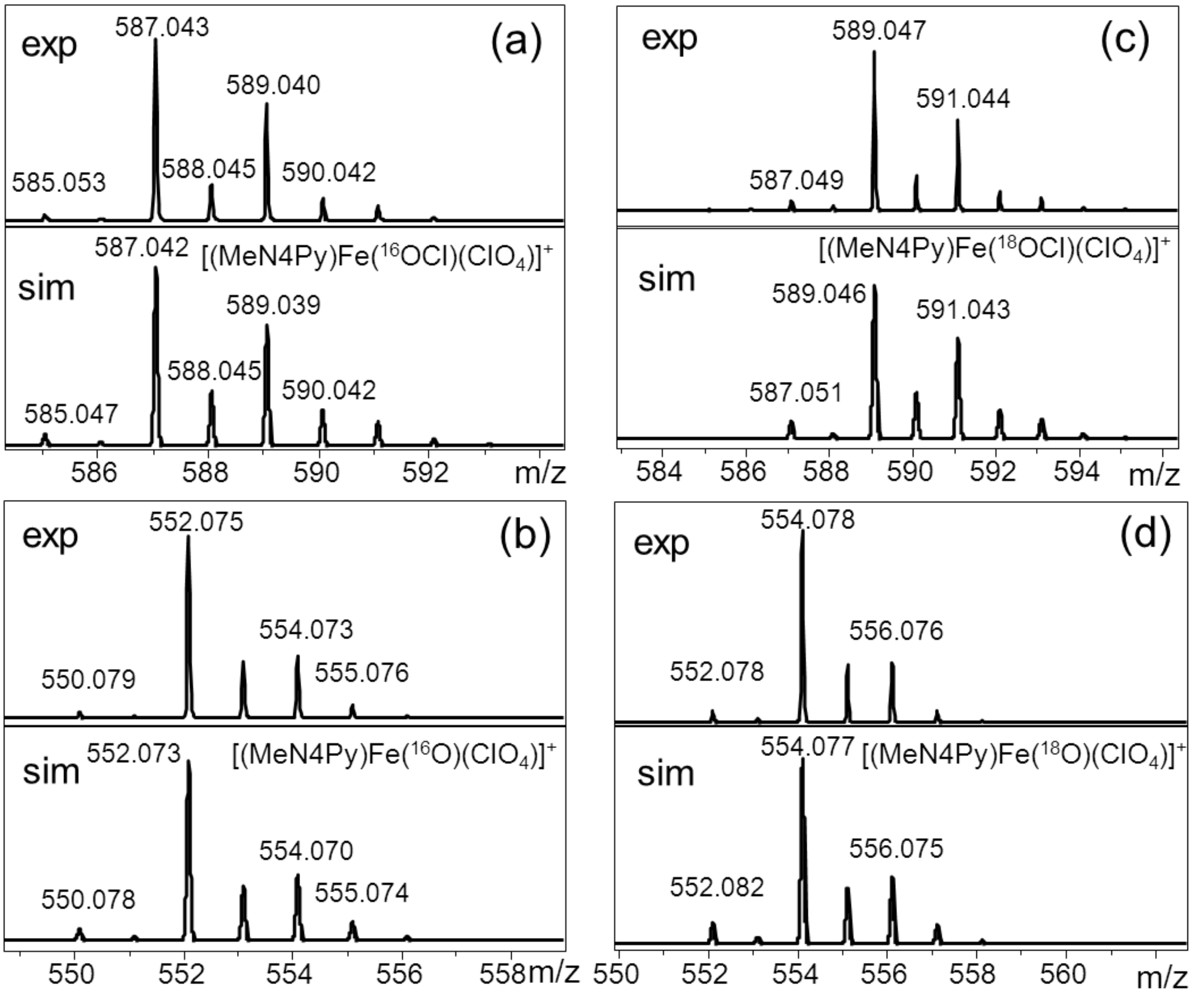

Figure S 9 Experimental and simulated Cryo ESI-MS spectra obtained from the reaction mixture containing NaOCl and [(MeN4Py)Fe^{II}(Cl)](Cl) in H_2O^{16} and H_2O^{18} at pH 2.5 (a) [(MeN4Py)Fe^{III}(OCl)(ClO_4)$]^+$ (m/z 587.04) (b) [(MeN4Py)Fe^{IV}(O)(ClO_4)$]^+$ (m/z 552.07) generated with

Na^{16}OCl and (c) [(MeN4Py)Fe^{III}(^{18}OCl)(ClO_4)]$^+$ (m/z 587.04) and (d) [(MeN4Py)Fe^{IV}(^{18}O)(ClO_4)]$^+$ (m/z 552.07) generated with Na^{18}OCl.

We carried out similar experiments using Ca($OCl)_2$ (as solid). As with NaOCl, addition of 2 or 4 equiv of Ca($OCl)_2$ to an aqueous solution of [(MeN4Py)Fe^{II}(Cl)]Cl (0.5 mM, pH 2.5), shows mono cationic ions [(MeN4Py)Fe^{III}(OCl)(ClO_4)]$^+$ (m/z 587.042), [(MeN4Py)Fe^{III}(OH)(ClO_4)]$^+$ (m/z 553.080), [(MeN4Py)Fe^{IV}(O)(ClO_4)]$^+$ (m/z 552.073) and [(MeN4Py)Fe^{II}(ClO4)]$^+$ (m/z 536.079) and dicationic ions [(MeN4Py)Fe^{III}(OH)]$^{2+}$ (m/z 227.066), [(MeN4Py)Fe^{IV}(O)]$^{2+}$ (m/z 226.563). With labelled Ca(^{18}OCl)$_2$ mono cationic ions except [(MeN4Py)Fe^{II}(ClO_4)]$^+$ (m/z 536.079), increased by two m/z units and dicationic ions were increased by one m/z unit (Figure S 9).

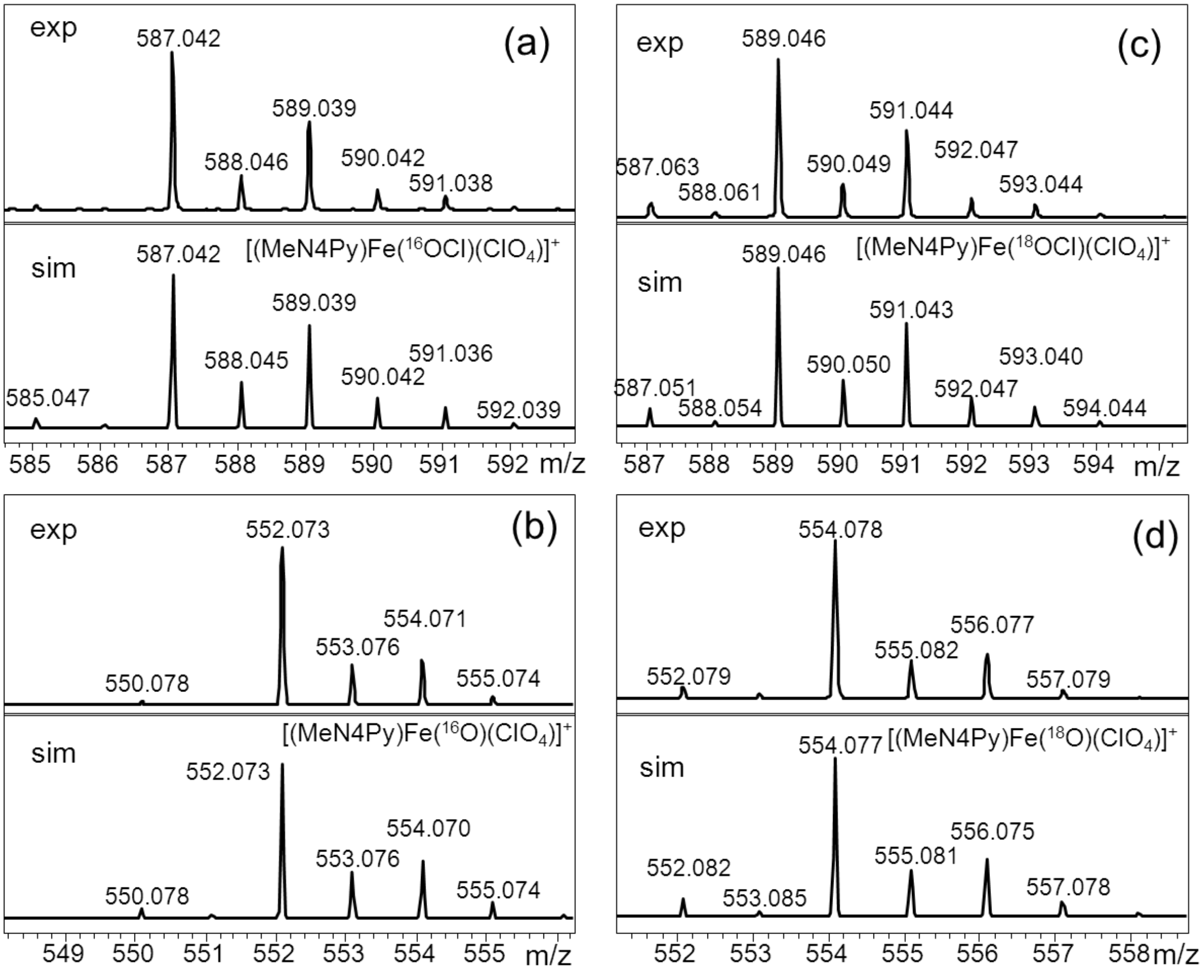

Figure S 10 Experimental and simulated Cryo ESI-MS spectra obtained from the reaction mixture contains Ca($OCl)_2$ and [(MeN4Py)Fe^{II}(Cl)]Cl in H_2O^{16} and H_2O^{18} at pH 2.5 (a) [(MeN4Py)Fe^{III}(OCl)(ClO_4)]$^+$ (m/z 587.04) (b) [(MeN4Py)Fe^{IV}(O)(ClO_4)]$^+$ (m/z 552.07) generated with Ca(^{16}OCl)$_2$ and (c) [(MeN4Py)Fe^{III}(^{18}OCl)(ClO_4)]$^+$ (m/z 587.04) and (d) [(MeN4Py)Fe^{IV}(^{18}O)(ClO_4)]$^+$ (m/z 552.07) generated with Ca(^{18}OCl)$_2$.

6. EPR spectroscopy

The EPR spectrum obtained from flash frozen samples when the absorbance at 495 nm was at a maximum (generated by adding 2 equiv of NaOCl to the aqueous solution of [(N4Py)Fe(CH_3CN)](ClO_4)$_2$, 1 mM, pH 2.8) shows signals at g = 2.42, 2.26, 2.16, 1.97 and 1.92 characteristic for low spin Fe(III) complexes. In contrast to [(MeN4Py)Fe^{II}(Cl)](Cl), the contribution of the high spin Fe(III) signal was higher (Figure S 7a). At medium pH (6.5), a signal related to a high spin Fe(III) species was observed (Figure S 7b).

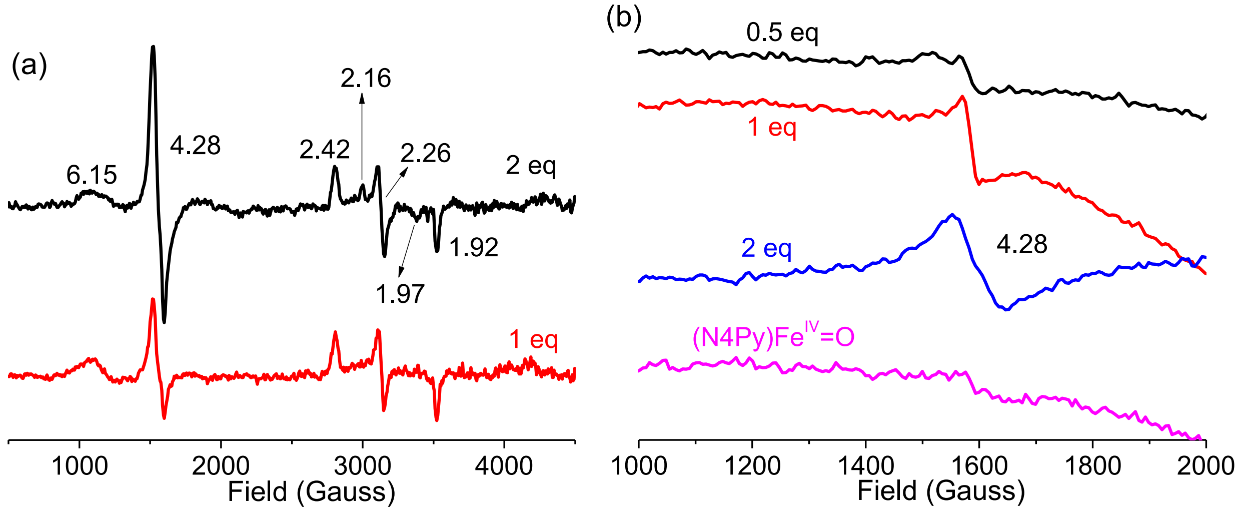

Figure S 11 (a) EPR spectra at 77 K of [(N4Py)Fe^{III}(OCl)]$^{2+}$ generated with 1 equiv (top) and 2 equiv (bottom) of NaOCl to [(N4Py)Fe(CH_3CN)](ClO_4)$_2$ in water (1 mM, pH 2.8) and (b) EPR spectra at 77 K of [(MeN4Py)Fe^{III}(OCl)]$^{2+}$ generated with 0.5 - 2 equiv of NaOCl to [(N4Py)Fe(CH_3CN)](ClO_4)$_2$ in water (1 mM, pH 6.5) and a solution of [(N4Py)Fe^{IV}(O)]$^{2+}$.

7. Raman spectroscopy

Addition of 2 equiv of Na^{18}OCl to a solution of [(MeN4Py)Fe^{II}(Cl)](Cl) (1 mM in ^{18}OH$_2$ at pH 2.2) results in the appearance of the bands at 673, 628 and 562 cm^{-1}. Again, these bands decrease in intensity over time with a concomitant appearance of a new band at 807 cm^{-1} (Figures S 10 and 11). Three bands are shifted when compared with the non ^{18}O labelled samples. The band at 673 cm^{-1} was not sensitive to ^{18}O labelling. The bands at 843, 653 and 580 cm^{-1} shift to 807, 628 and 562 cm^{-1}. The observed shift of 36 cm^{-1} of the band at 843 cm^{-1} is in good agreement with the calculated shift (for a two atom approximation) for a Fe-O bond (37 cm^{-1}). This is in agreement with the assignment of the species formed later in the reaction with an absorption at 670 nm as the [(MeN4Py)Fe^{IV}(O)]$^{2+}$ complex.[7] The bands 653 and 580 cm^{-1} were shifted by 25 and 18 cm^{-1}, respectively, these shifts are close to those expected for an Fe-O bond (29 cm^{-1} for the band at 653 cm^{-1} and 26 cm^{-1} for the band at 580 cm^{-1}) and O-Cl (26 cm^{-1} for the band at 653 cm^{-1} and 23 cm^{-1} for the band at 580 cm^{-1}) modes. Hence definitive assignment of the mode cannot be made on the basis of isotope shift.

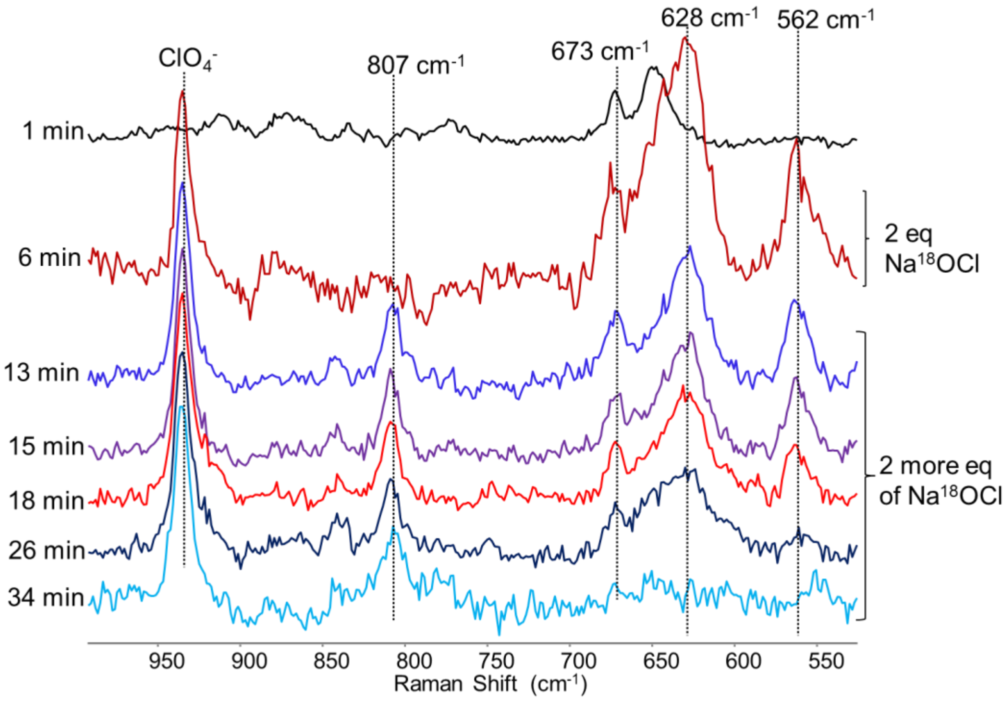

Figure S 12 Reaction between [(MeN4Py)Fe^{II}(Cl)]Cl (4 mM in ^{18}OH$_2$ at pH 2.2) and Na^{18}OCl (two additions of two equiv in ^{18}OH$_2$) followed by Raman spectroscopy at λ_{exc} 473 nm. Spectra were normalized to the ClO_4^- band at 934 cm^{-1} except for the initial spectrum. The legend is time in minutes after addition of Na^{18}OCl.

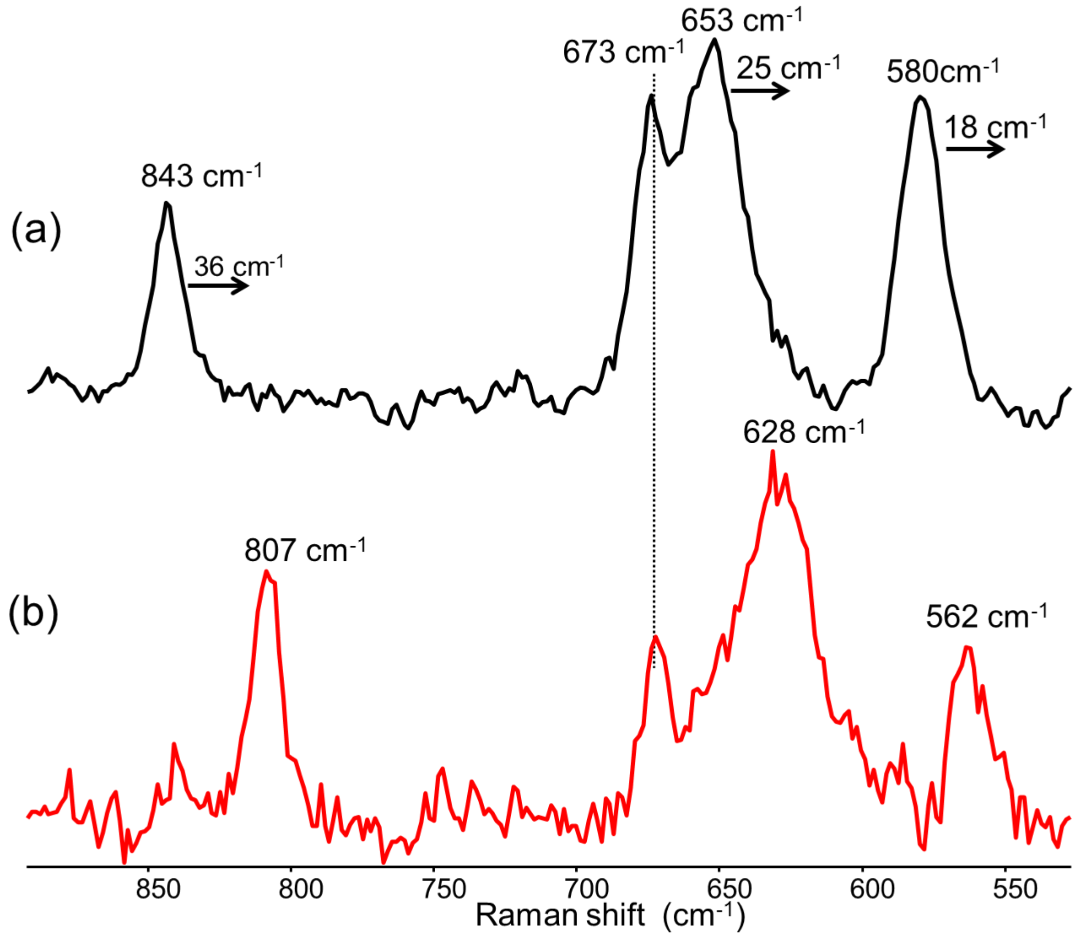

Figure S 13 Intermediates generated upon the reaction of [(MeN4Py)Fe^{II}(Cl)]Cl (4 mM at pH 2.2) with (a) $Na^{16}OCl$ in $^{16}OH_2$ and (b) $Na^{18}OCl$ in $^{18}OH_2$ followed by Raman spectroscopy at λ_{exc} 473 nm

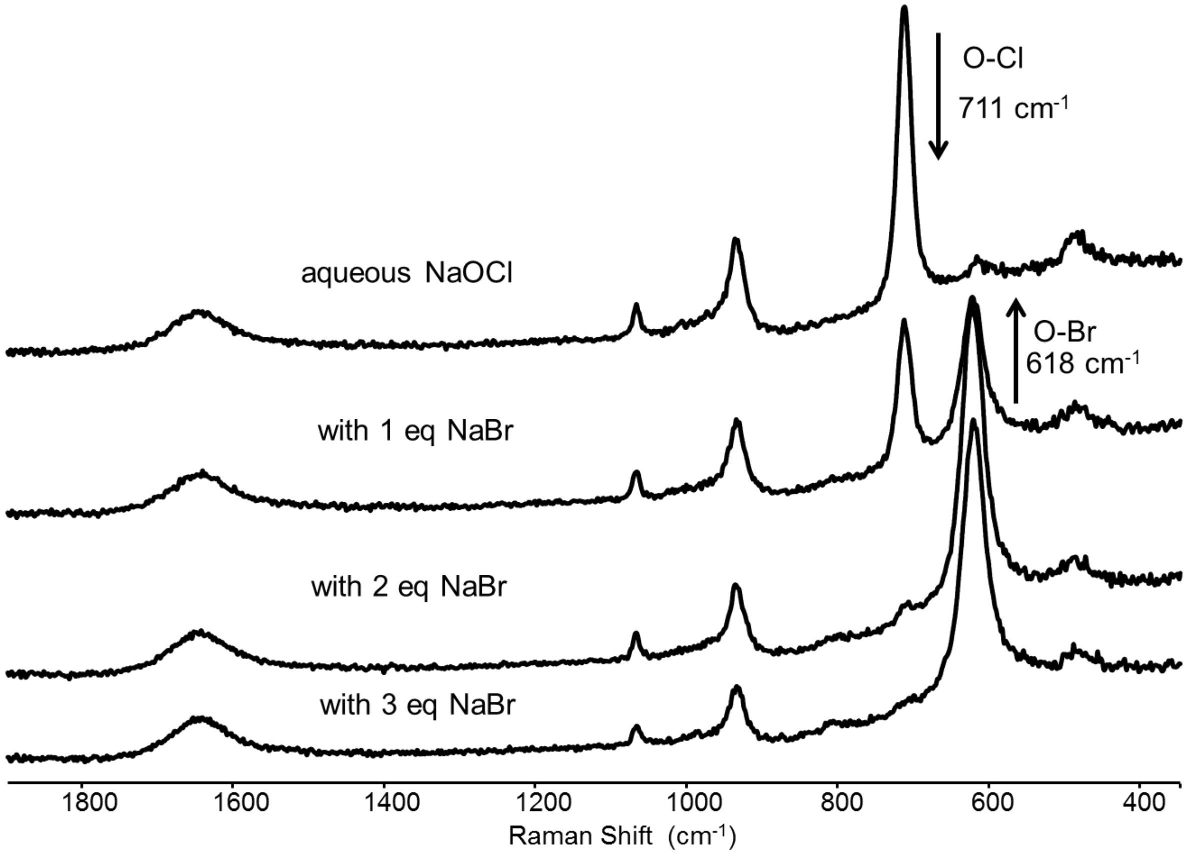

Figure S 14 Raman Spectra of aqueous NaOCl before and after addition of NaBr at λ_{exc} 785 nm. Spectra were normalized to the water band at ca. 1650 cm^{-1}.

NaOBr was employed to facilitate band assignments. Addition of 2 equiv of NaOBr[9] to an aqueous solution of [(MeN4Py)Fe^{II}(Cl)](Cl) (1 mM in H_2O at pH 2.2), results in substantial interference from fluorescence and, hence, it is not possible to obtain Raman spectra under the conditions employed. The solvent system was changed to water/acetonitrile (1:1) to circumvent this problem. As with water, addition of 2 equiv of NaOCl to a solution of [(MeN4Py)Fe^{II}(Cl)](Cl) (1:1 water/acetonitrile at pH 2.2) shows bands at 580, 656, 676 and 843 cm^{-1} (Figure S 13). Addition of 2 equiv of NaOBr instead of NaOCl shows the bands 843, 673 and 629 cm^{-1}. The band at 843 cm^{-1} was not affected. The band at 676 was moderately shifted (3 cm^{-1}) by replacement of Cl with Br, this band is also oxygen insensitive indicating that it is a Fe-N mode. The 656 cm^{-1} band was shifted to 629 cm^{-1}, the observed shift of 27 cm^{-1} is too small to be assign to be due to an O-Br vibrational mode. Interestingly, the band at 580 cm^{-1} was not observed. Even though Cong et al.[10] assigned the 786 cm^{-1} band in the heme (Fe^{III}-OCl) system as an O-Cl vibrational mode, ^{18}O labelling and bromine labelling data support the assignment of the 580 cm^{-1} band as the O-Cl vibrational mode. The other oxygen sensitive mode at 656 cm^{-1} was tentatively assigned to an Fe^{III}-O stretch. The shift of 27 cm^{-1} might be due to the effect of bromine on the Fe-O stretch (i.e. a change in force constant).

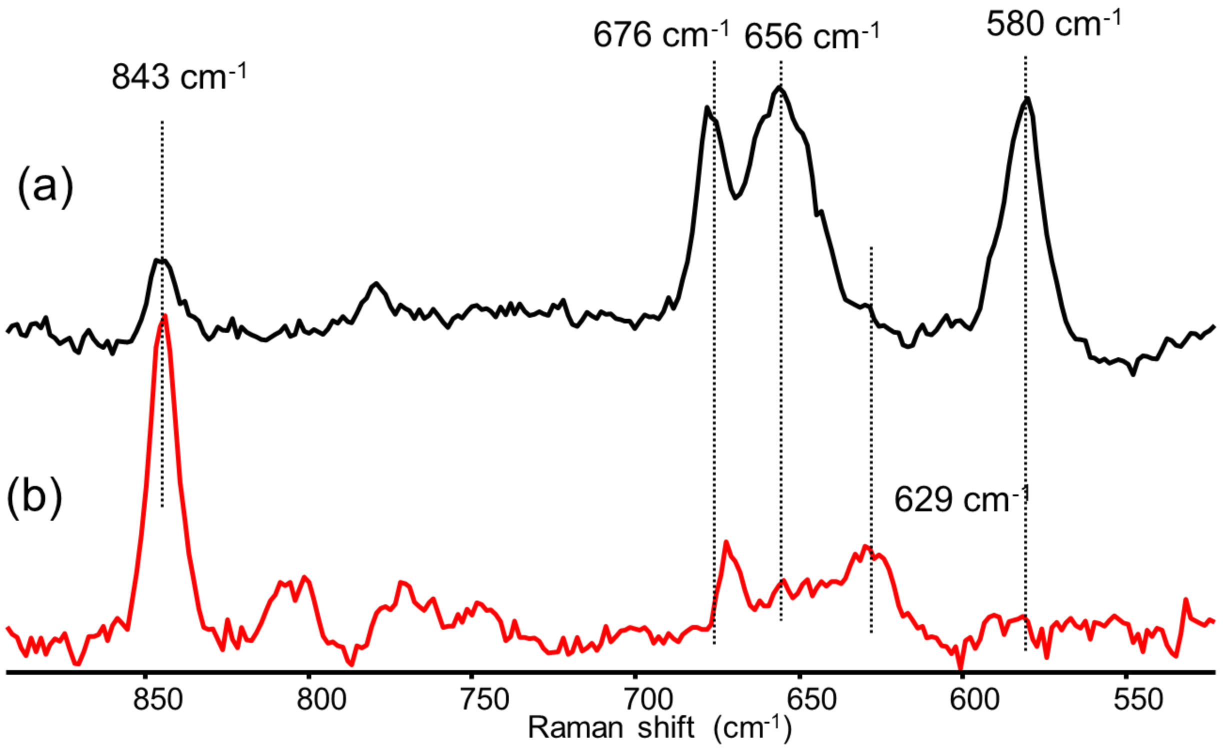

Figure S 15 Reaction of [(MeN4Py)Fe^{II}(Cl)]Cl (4 mM in H_2O at pH 2.2) with (a) NaOCl and (b) NaOBr followed by Raman spectroscopy at λ_{exc} 473 nm.

Resonance Raman spectra were calculated for the Fe(III)-OCl (doublet) and the Fe(IV)=O (triplet) species using ORCA (version 3.0.2) and the IMDHO method as implemented in the orca_asa program.[11] The procedure used was as follows:

The geometry optimization and the Hessian matrix were calculated with the Becke89[12] and Parr86[13] (BP86) DFT functional using Def2-TZVP basis set.[14] The Hessian matrix was used in the TD-DFT [15] calculation, which was performed using the BH and HLYP functional with the TZVPP[14a] basis set for Iron and SV(P)[14a,16] basis set for C, H, N, O, Cl. ^{18}O isotope shifts were calculated with Gaussian.

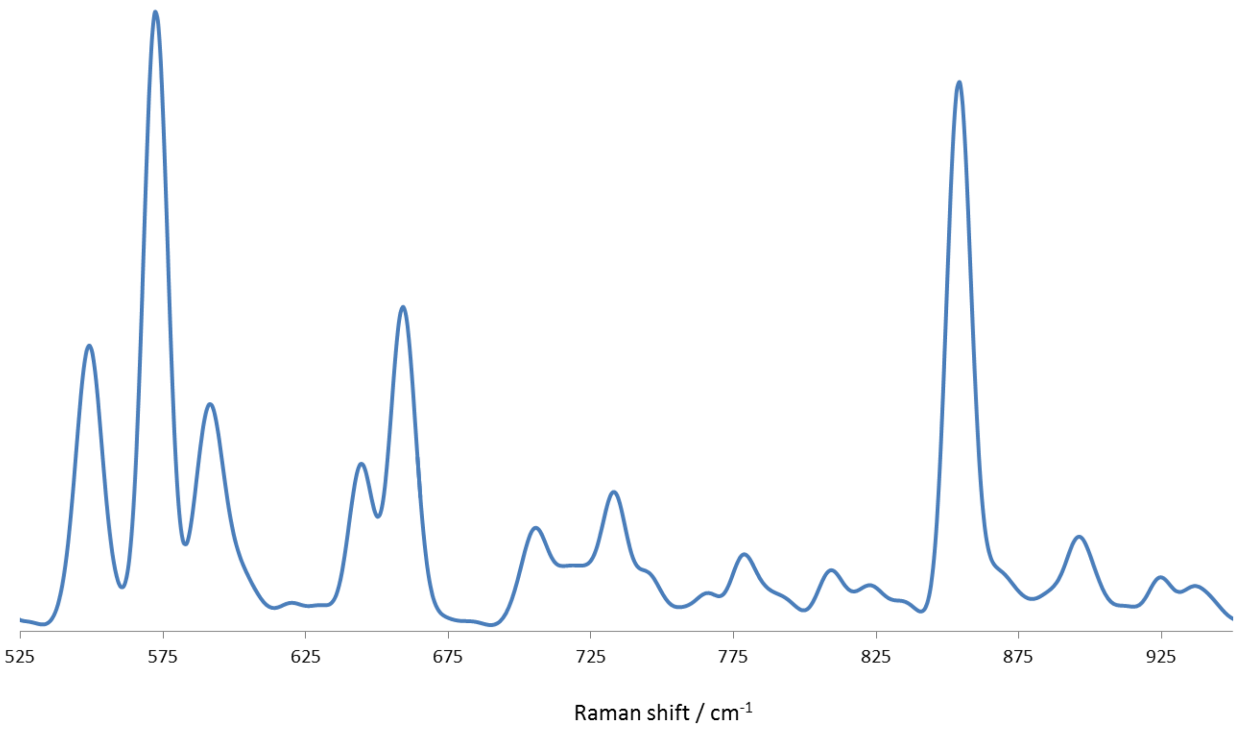

Figure S16 Simulation of the rR spectrum of the Fe(III)-OCl species (excitation at 502 nm). The modes that show the greatest degree of enhancement are: 549 cm^{-1} (O-Cl stretching mode), 572 cm^{-1} (N_{amino}-Fe stretching mode), 592 cm^{-1} (Fe-O-Cl Bending mode), 644 cm^{-1} (symmetric Py-Fe breathing), 658 cm^{-1} (Py ring breathing with minor influence on OCl), 853 cm-1 (N_{amino} bending mode). See the PPS file for vibrational modes.

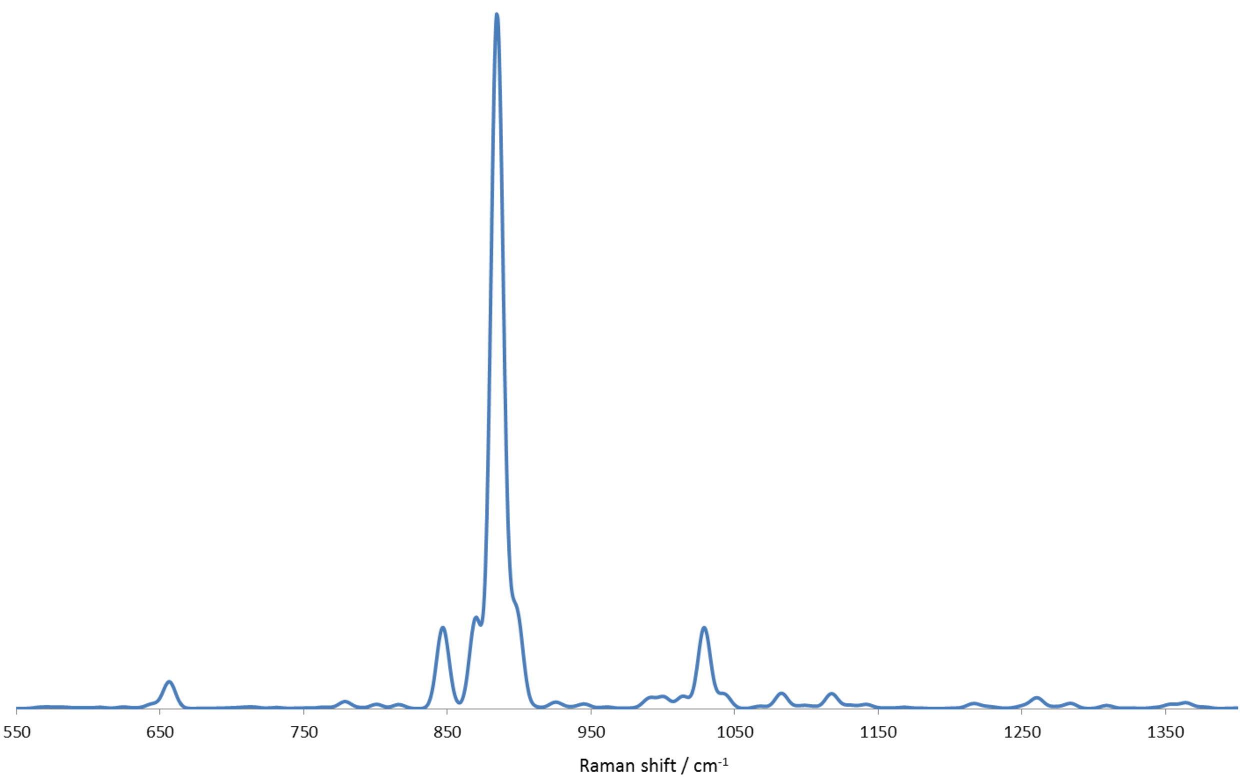

Figure S19 Simulation of the rR spectrum of the Fe(IV)=O species (excited at 911 nm). The mode that shows the greatest degree of enhancement is at 884 cm^{-1} with a mode enhanced to a lesser extent at 845 cm^{-1} . The former mode involves primarily an Fe-O stretching displacement. See the PPS file for vibrational modes.

8. Comproportionation between Fe(II)OH$_2$ and Fe(IV)=O

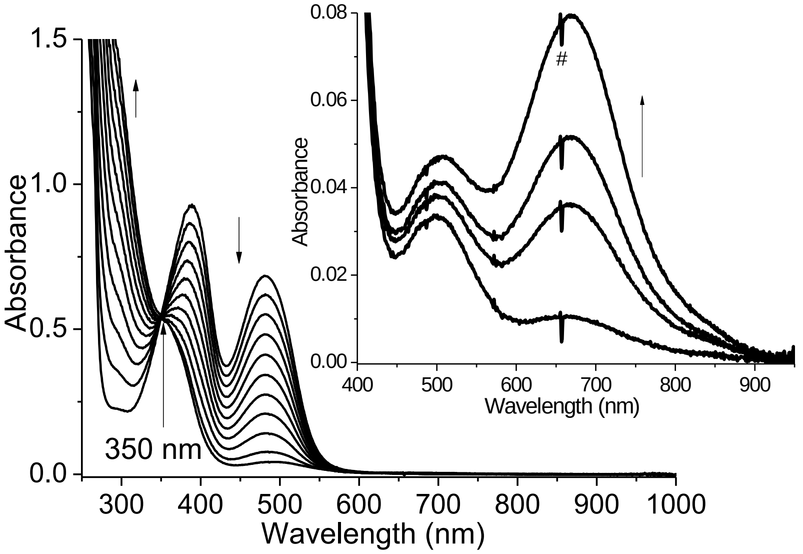

Figure S 18 Addition of an aqueous solution of [(MeN4Py)Fe^{IV}(O)](PF$_6$)$_2$ (2 mM, 10 μL per addition) to an aqueous solution of [(MeN4Py)Fe^{II}(CH$_3$CN)](ClO$_4$)$_2$ (0.25 mM, 1 mL, with stirring) in water (inset: after addition of 1 equiv of [(MeN4Py)Fe^{IV}(O)](PF$_6$)$_2$ complete disappearance of the absorption bands of the iron(II) complex, further addition of [(MeN4Py)Fe^{IV}(O)](PF$_6$)$_2$ leads to its characteristic absorption at 670 nm). Spectra are corrected for dilution.

9. Computational Details

Computational studies were performed to gain insight into the electronic features of the complexes and the reaction mechanism. Each structure described here was found through a full geometry optimization and characterized as a local minimum with a frequency calculation. To assist with the interpretation of experimental resonance Raman spectra, we also calculated Raman vibrations from the frequencies. All calculations mentioned here were performed using Density Functional Theory (DFT) with the unrestricted B3LYP method[17] as implemented in the Gaussian 09c program package.[18] Single point calculations with B3LYP-D3[19] were calculated using Jaguar V3.0 Rev 2.[20] All calculations utilized a triple-ζ quality basis set that includes LANL2TZ+(f) on iron and 6-311+G(d,p) on the rest of the atoms, basis set BS1.[21] Free energies (ΔG) reported here use UB3LYP-D3 energies corrected with ZPE, thermal and entropic corrections from the frequency calculation at 298 K. The effect of solvent was tested through single point calculations using water as a solvent with the SMD solvation model as implemented in Gaussian.

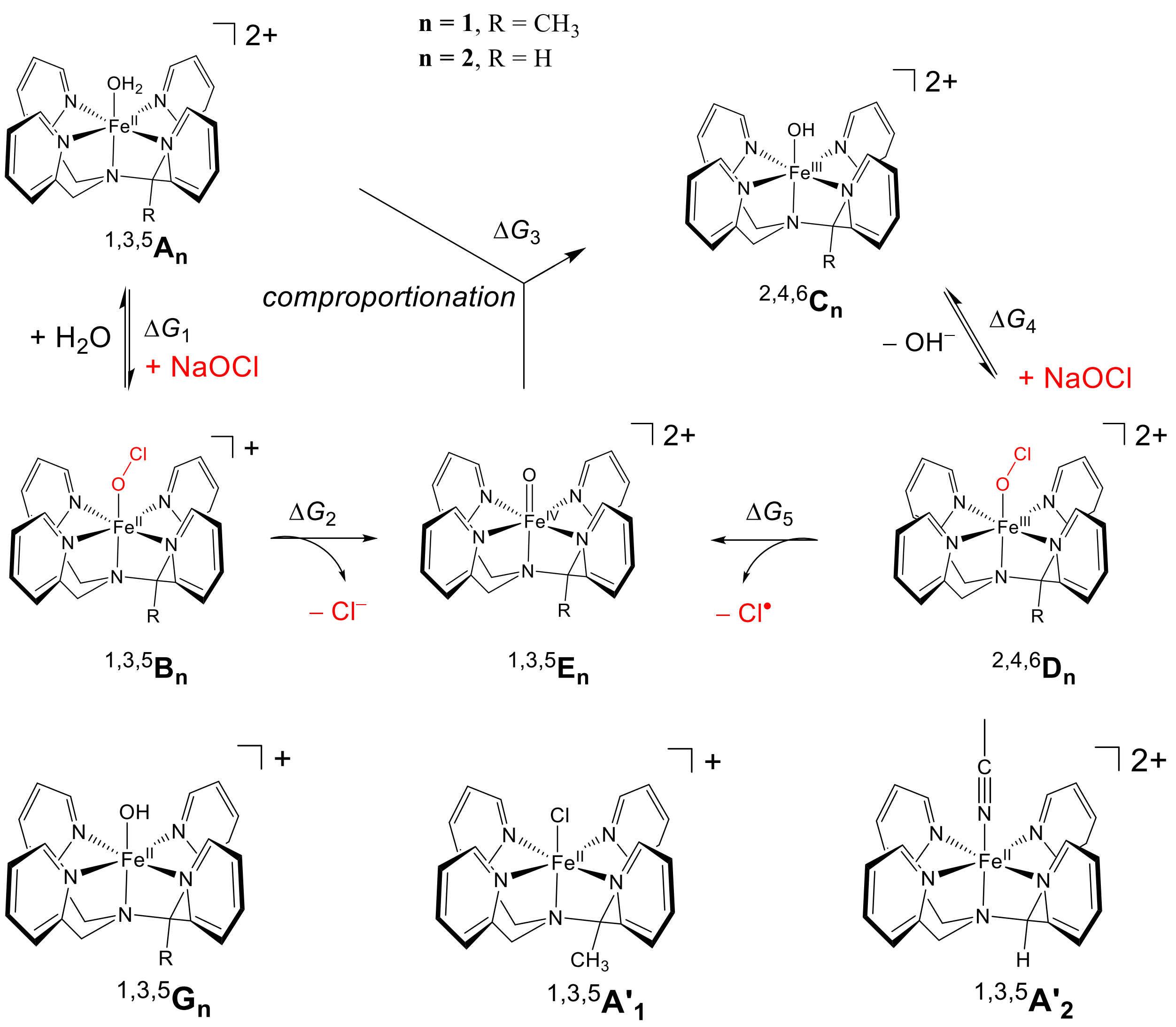

Figure S 19 Scheme of the species studied. The number in superscript refers to the possible spin state of the molecule.

Part 1: Spin densities and Charges for N4Py-H ligand.

Table S 1: Group spin densities of UB3LYP/BS1 optimized geometries of $^{1,3,5}\mathbf{A_2}$ in Gaussian.

	Spin densities			Charges		
	ρ_{Fe}	ρ_{H2O}	ρ_{N4Py}	Q_{Fe}	Q_{H2O}	Q_{N4Py}
$^1\mathbf{A_2}$	0.00	0.00	0.00	-0.49	0.09	2.40
$^3\mathbf{A_2}$	2.16	0.01	-0.17	-0.17	-0.03	2.20
$^5\mathbf{A_2}$	3.27	0.03	0.70	-0.34	0.17	2.17

Table S 2: Group spin densities of UB3LYP/BS1 optimized geometries of $^{1,3,5}\mathbf{B_2}$ in Gaussian.

	Spin densities			Charges		
	ρ_{Fe}	ρ_{OCl}	ρ_{N4Py}	Q_{Fe}	Q_{OCl}	Q_{N4Py}
$^1\mathbf{B_2}$	0.00	0.00	0.00	-0.91	-0.46	2.37
$^3\mathbf{B_2}$	1.99	0.06	-0.05	-2.25	-0.14	3.39
$^5\mathbf{B_2}$	3.82	0.12	0.06	-1.71	-0.18	2.88

Table S 3: Group spin densities of UB3LYP/BS1 optimized geometries of $^{1,3,5}\mathbf{E_2}$ in Gaussian.

	Spin densities			Charges		
	ρ_{Fe}	ρ_{O}	ρ_{N4Py}	Q_{Fe}	Q_{O}	Q_{N4Py}
$^1\mathbf{E_2}$	0.00	0.00	0.00	-0.62	-0.70	3.32
$^3\mathbf{E_2}$	1.37	0.85	-0.22	-0.59	-0.70	3.29
$^5\mathbf{E_2}$	2.51	0.98	0.52	-1.07	-0.47	3.55

Table S 4: Group spin densities of UB3LYP/BS1 optimized geometries of $^{2,4,6}\mathbf{C_2}$ in Gaussian.

	Spin densities			Charges		
	ρ_{Fe}	ρ_{OH}	ρ_{N4Py}	Q_{Fe}	Q_{OH}	Q_{N4Py}
$^2\mathbf{C_2}$	1.04	0.13	-0.17	-0.84	-0.79	3.64
$^4\mathbf{C_2}$	2.83	0.30	-0.13	-0.59	-0.49	3.08
$^6\mathbf{C_2}$	4.23	0.35	0.41	-0.52	-0.41	2.93

Table S 5: Group spin densities of UB3LYP/BS1 optimized geometries of $^{2,4,6}\mathbf{D_2}$ in Gaussian.

	Spin densities			Charges		
	ρ_{Fe}	ρ_{OCl}	ρ_{N4Py}	Q_{Fe}	Q_{OCl}	Q_{N4Py}
$^2\mathbf{D_2}$	1.03	0.12	-0.15	-3.00	0.01	4.98
$^4\mathbf{D_2}$	2.90	0.29	-0.19	-3.19	0.02	5.18
$^6\mathbf{D_2}$	4.25	0.37	0.38	-2.66	0.13	4.53

Table S 6: Group spin densities of UB3LYP/BS1 optimized geometries of $^{1,3,5}\mathbf{A'_2}$ in Gaussian.

	Spin densities			Charges		
	ρ_{Fe}	ρ_{NCMe}	ρ_{N4Py}	Q_{Fe}	Q_{NCMe}	Q_{N4Py}
$^1\mathbf{A_2}$	0.00	0.00	0.00	-0.67	0.07	2.60
$^3\mathbf{A_2}$	2.05	0.01	-0.06	-0.73	0.19	2.54
$^5\mathbf{A_2}$	3.76	0.06	0.18	0.35	0.04	1.62

Table S 7: Group spin densities of UB3LYP/BS1 optimized geometries of $^{1,3,5}\mathbf{G_2}$ in Gaussian.

	Spin densities			Charges		
	ρ_{Fe}	ρ_{OH}	ρ_{N4Py}	Q_{Fe}	Q_{OH}	Q_{N4Py}
$^1\mathbf{G_2}$	0.00	0.00	0.00	-0.61	-0.53	2.14
$^3\mathbf{G_2}$	1.99	0.08	-0.07	-0.23	-0.46	1.69
$^5\mathbf{G_2}$	3.80	0.15	0.03	-0.40	-0.49	2.03

Part 2: Spin Densities and Charges for N4Py-Me ligand:

Table S 8: Group spin densities of UB3LYP/BS1 optimized geometries of $^{1,3,5}\mathbf{A_1}$ in Gaussian.

	Spin densities			Charges		
	ρ_{Fe}	ρ_{H2O}	ρ_{N4Py}	Q_{Fe}	Q_{H2O}	Q_{N4Py}
$^1\mathbf{A_1}$	0.00	0.00	0.00	-0.51	0.09	2.42
$^3\mathbf{A_1}$	1.21	0.00	0.79	-0.45	0.15	2.30
$^5\mathbf{A_1}$	3.24	0.03	0.72	-0.35	0.16	2.18

Table S 9: Group spin densities of UB3LYP/BS1 optimized geometries of $^{1,3,5}\mathbf{B_1}$ in Gaussian.

	Spin densities			Charges		
	ρ_{Fe}	ρ_{OCl}	ρ_{N4Py}	Q_{Fe}	Q_{OCl}	Q_{N4Py}
$^1\mathbf{B_1}$	0.00	0.00	0.00	-0.95	-0.46	2.41
$^3\mathbf{B_1}$	2.01	0.06	-0.06	-2.47	-0.14	3.61
$^5\mathbf{B_1}$	3.82	0.12	0.06	-1.87	-0.17	3.04

Table S 10: Group spin densities of UB3LYP/BS1 optimized geometries of $^{1,3,5}\mathbf{E_1}$ in Gaussian.

	Spin densities			Charges		
	ρ_{Fe}	ρ_{O}	ρ_{N4Py}	Q_{Fe}	Q_{O}	Q_{N4Py}
$^1\mathbf{E_1}$	0.00	0.00	0.00	-0.62	-0.70	3.32
$^3\mathbf{E_1}$	1.39	0.85	-0.23	-0.58	-0.71	3.29
$^5\mathbf{E_1}$	3.23	1.07	-0.30	-0.68	-0.83	3.51

Table S 11: Group spin densities of UB3LYP/BS1 optimized geometries of $^{2,4,6}\mathbf{E_1}$ in Gaussian.

	Spin densities			Charges		
	ρ_{Fe}	ρ_{OH}	ρ_{N4Py}	Q_{Fe}	Q_{OH}	Q_{N4Py}
$^2\mathbf{E_1}$	1.05	0.12	-0.17	-0.86	-0.50	3.36
$^4\mathbf{E_1}$	2.83	0.29	-0.12	-0.51	-0.49	3.00
$^6\mathbf{E_1}$	4.23	0.35	0.43	-0.47	-0.41	2.87

Table S 12: Group spin densities of UB3LYP/BS1 optimized geometries of $^{2,4,6}\mathbf{D_1}$ in Gaussian.

	Spin densities			Charges		
	ρ_{Fe}	ρ_{OCl}	ρ_{N4Py}	Q_{Fe}	Q_{OCl}	Q_{N4Py}
$^2\mathbf{D_1}$	1.01	0.12	-0.13	-1.55	-0.13	3.68
$^4\mathbf{D_1}$	2.89	0.28	-0.17	-3.40	0.00	5.40
$^6\mathbf{D_1}$	4.24	0.36	0.40	-2.79	0.12	4.67

Table S 13: Group spin densities of UB3LYP/BS1 optimized geometries of $^{1,3,5}\mathbf{A'_1}$ in Gaussian.

	Spin densities			Charges		
	ρ_{Fe}	ρ_{Cl}	ρ_{N4Py}	Q_{Fe}	Q_{Cl}	Q_{N4Py}
$^1\mathbf{A'_1}$	0.00	0.00	0.00	-0.80	-0.51	2.31
$^3\mathbf{A'_1}$	2.25	0.07	-0.32	-0.49	-1.02	2.50
$^5\mathbf{A'_1}$	3.71	0.17	0.12	0.07	-0.92	1.85

Table S 14: Group spin densities of UB3LYP/BS1 optimized geometries of $^{1,3,5}\mathbf{G_1}$ in Gaussian.

	Spin densities			Charges		
	ρ_{Fe}	ρ_{OH}	ρ_{N4Py}	Q_{Fe}	Q_{OH}	Q_{N4Py}
$^1\mathbf{G_1}$	0.00	0.00	0.00	-0.62	-0.75	2.38
$^3\mathbf{G_1}$	2.01	0.06	-0.07	-0.10	-0.68	1.78

^{5}**G$_1$**	3.83	0.13	0.04	-0.17	-0.58	1.75

Part 3: Absolute energies for N4Py-H ligand.

Table S 15: Absolute energy of UB3LYP/BS1 optimized geometry of $^{1,3,5}\mathbf{A_2}$ in Gaussian.

	E, au	ZPE, au	G, au	E_{disp}, kcal	E_{solv}, kcal
$^{1}\mathbf{A_2}$	-1362.683	0.429	-1362.306	-46.21	-142.57
$^{3}\mathbf{A_2}$	-1362.668	0.427	-1362.297	-47.15	-140.13
$^{5}\mathbf{A_2}$	-1362.689	0.426	-1362.322	-45.06	-140.55

Table S 16: Absolute energy of UB3LYP/BS1 optimized geometry of $^{1,3,5}\mathbf{B_2}$ in Gaussian.

	E, au	ZPE, au	G, au	E_{disp}, kcal	E_{solv}, kcal
$^{1}\mathbf{B_2}$	-1821.932	0.407	-1821.578	-48.13	-46.82
$^{3}\mathbf{B_2}$	-1821.919	0.406	-1821.571	-47.30	-60.17
$^{5}\mathbf{B_2}$	-1821.943	0.404	-1821.599	-46.32	-44.03

Table S 17: Absolute energy of UB3LYP/BS1 optimized geometry of $^{1,3,5}\mathbf{E_2}$ in Gaussian.

	E, au	ZPE, au	G, au	E_{disp}, kcal	E_{solv}, kcal
$^{1}\mathbf{E_2}$	-1361.361	0.408	-1361.004	-44.91	-141.05
$^{3}\mathbf{E_2}$	-1361.411	0.408	-1361.054	-44.90	-141.33
$^{5}\mathbf{E_2}$	-1361.398	0.406	-1361.046	-42.92	-140.10

Table S 18: Absolute energy of UB3LYP/BS1 optimized geometry of $^{1,3,5}\mathbf{C_2}$ in Gaussian.

	E, au	ZPE, au	G, au	E_{disp}, kcal	E_{solv}, Kcal
$^{2}\mathbf{C}_2$	-1362.063	0.419	-1361.696	-45.81	-140.82
$^{4}\mathbf{C}_2$	-1362.044	0.416	-1361.682	-45.15	-139.23
$^{6}\mathbf{C}_2$	-1362.067	0.415	-1361.707	-45.42	-137.71

Table S 19: Absolute energy of UB3LYP/BS1 optimized geometry of $^{1,3,5}\mathbf{D_2}$ in Gaussian.

	E, au	ZPE, au	G, au	E_{disp}, kcal	E_{solv}, Kcal
$^{2}\mathbf{D}_2$	-1821.624	0.410	-1821.268	-48.49	-139.15
$^{4}\mathbf{D}_2$	-1821.601	0.407	-1821.250	-47.22	-136.33
$^{6}\mathbf{D}_2$	-1821.619	0.407	-1821.273	-46.74	-135.29

Table S 20: Absolute energy of UB3LYP/BS1 optimized geometry of $^{1,3,5}\mathbf{A''_2}$ in Gaussian.

	E, au	ZPE, au	G, au	E_{disp}, kcal	E_{solv}, kcal
$^{1}\mathbf{A''}_2$	-1419.045	0.452	-1418.650	-47.46	-132.77
$^{3}\mathbf{A''}_2$	-1419.022	0.449	-1418.633	-45.60	-131.25
$^{5}\mathbf{A''}_2$	-1419.043	0.448	-1418.658	-45.75	-131.00

Table S 21: Absolute energy of UB3LYP/BS1 optimized geometry of ^{1,3,5}**G$_2$** in Gaussian.

	E, au	ZPE, au	G, au	E_{disp}, kcal	E_{solv}, kcal
^{1}**G**$_2$	-1362.360	0.417	-1361.994	-43.65	-49.46
^{3}**G**$_2$	-1362.347	0.415	-1361.987	-43.53	-51.14
^{5}**G**$_2$	-1362.381	0.412	-1362.026	-42.99	-49.42

Part 4: Absolute energies for N4Py-Me ligand.

Table S 22: Absolute energy of UB3LYP/BS1 optimized geometry of ^{1,3,5}**A$_1$** in Gaussian.

	E, au	ZPE, au	G, au	E_{disp}, kcal	E_{solv}, Kcal
^{1}**A**$_1$	-1402.009	0.457	-1401.605	-51.014	-141.06
^{3}**A**$_1$	-1401.993	0.455	-1401.594	-51.900	-138.48
^{5}**A**$_1$	-1402.011	0.454	-1401.616	-49.946	-140.09

Table S 23: Absolute energy of UB3LYP/BS1 optimized geometry of ^{1,3,5}**B$_1$** in Gaussian.

	E, au	ZPE, au	G, au	E_{disp}, kcal	E_{solv}, Kcal
^{1}**B**$_1$	-1861.256	0.435	-1860.876	-52.914	-45.50
^{3}**B**$_1$	-1861.241	0.433	-1860.866	-50.327	-43.34
^{5}**B**$_1$	-1861.264	0.432	-1860.893	-51.308	-43.52

Table S 24: Absolute energy of UB3LYP/BS1 optimized geometry of ^{1,3,5}**E$_1$** in Gaussian.

	E, au	ZPE, au	G, au	E_{disp}, kcal	E_{solv}, Kcal
^{1}**E**$_1$	-1400.689	0.435	-1400.305	-47.453	-138.81
^{3}**E**$_1$	-1400.738	0.436	-1400.354	-47.428	-139.28
^{5}**E**$_1$	-1400.724	0.434	-1400.345	-45.603	-138.35

Table S 25: Absolute energy of UB3LYP/BS1 optimized geometry of ^{2,4,6}**C$_1$** in Gaussian.

	E, au	ZPE, au	G, au	E_{disp}, kcal	E_{solv}, Kcal
^{2}**C**$_1$	-1401.391	0.447	-1400.996	-50.253	-139.05
^{4}**C**$_1$	-1401.370	0.444	-1400.981	-49.860	-137.30
^{6}**C**$_1$	-1401.391	0.443	-1401.004	-50.587	-135.21

Table S 26: Absolute energy of UB3LYP/BS1 optimized geometry of ^{2,4,6}**F$_1$** in Gaussian.

	E, au	ZPE, au	G, au	E_{disp}, kcal	E_{solv}, Kcal
^{2}**D**$_1$	-1860.951	0.437	-1860.568	-52.381	-137.29
^{4}**D**$_1$	-1860.926	0.435	-1860.549	-51.640	-134.87

**^{6}D$_1$**	-1860.944	0.434	-1860.570	-53.284	-133.91

Table S 27: Absolute energy of UB3LYP/BS1 optimized geometry of **^{1,3,5}A'$_1$** in Gaussian.

	E, au	ZPE, au	G, au	E_{disp}, kcal	E_{solv}, Kcal
**^{1}A'$_1$**	-1786.103	0.433	-1785.723	-51.674	-48.98
**^{3}A'$_1$**	-1786.082	0.431	-1785.706	-50.487	-49.41
**^{5}A'$_1$**	-1786.115	0.430	-1785.743	-50.671	-45.56

Table S 28: Absolute energy of UB3LYP/BS1 optimized geometry of **^{1,3,5}G$_1$** in Gaussian.

	E, au	ZPE, au	G, au	E_{disp}, kcal	E_{solv}, Kcal
**^{1}G$_1$**	-1401.685	0.444	-1401.293	-51.014	-45.52
**^{3}G$_1$**	-1401.669	0.442	-1401.282	-51.900	-52.69
**^{5}G$_1$**	-1401.701	0.441	-1401.318	-49.946	-43.13

Table S 29: Absolute energy of UB3LYP/BS1 optimized geometry of various reactants in Gaussian.

	E, au	ZPE, au	G, au	E_{disp}, kcal	E_{solv}, Kcal
Cl^*	-460.167	0.000	-460.183	0.000	1.78
Cl^-	-460.304	0.000	-460.319	0.000	-76.14
HClO	-536.007	0.013	-536.017	-0.107	-4.74
ClO^-	-535.433	0.001	-535.453	-0.010	-82.23
H_3O^+	-76.731	0.034	-76.716	0.000	-111.84
H_2O	-76.458	0.021	-76.455	0.000	-9.12
OH^-	-75.827	0.009	-75.835	0.000	-108.70
Cl-Cl	-920.410	0.001	-920.430	-0.018	1.88
NCMe	-132.796157	0.045149	-132.775024	-0.627	-5.3041

Part 5: Cartesian coordinates N4Py-H species:

Table S 30: Optimized Cartesian xyz coordinates of $^{1}A''_{2}$ calculated using UB3LYP/BS1 in Gaussian.

C	1.747897000	2.403098000	1.054864000
C	2.786481000	3.278525000	0.755179000
C	3.498084000	3.101388000	-0.427098000
C	3.148763000	2.051530000	-1.277334000
C	2.105605000	1.218205000	-0.902815000
N	1.414766000	1.386797000	0.246662000
C	1.638988000	0.000001000	-1.683579000
C	2.105603000	-1.218205000	-0.902817000
C	3.148762000	-2.051528000	-1.277336000
C	3.498082000	-3.101387000	-0.427103000
C	2.786479000	-3.278527000	0.755174000
C	1.747894000	-2.403102000	1.054860000
N	1.414763000	-1.386799000	0.246659000
N	0.127850000	0.000002000	-1.619494000
C	-0.498306000	-1.242493000	-2.172961000
C	-0.498304000	1.242499000	-2.172959000
C	-1.558474000	1.786472000	-1.241005000
C	-1.558474000	-1.786468000	-1.241007000
C	-2.532777000	-2.675798000	-1.681993000
C	-3.437213000	-3.200293000	-0.764035000
C	-3.341501000	-2.811142000	0.569127000
C	-2.351406000	-1.909461000	0.931729000
N	-1.469765000	-1.403732000	0.050745000
N	-1.469762000	1.403737000	0.050747000
C	-2.351403000	1.909463000	0.931732000
C	-3.341502000	2.811142000	0.569130000
C	-3.437216000	3.200291000	-0.764031000
C	-2.532780000	2.675798000	-1.681991000
Fe	-0.061530000	0.000001000	0.380302000
N	-0.139653000	-0.000001000	2.340351000
C	-0.107236000	-0.000004000	3.493137000
C	-0.062017000	-0.000008000	4.945627000
H	1.163645000	2.511433000	1.959564000
H	3.025501000	4.083494000	1.438236000
H	4.308323000	3.771162000	-0.689001000
H	3.675527000	1.889704000	-2.209951000
H	2.022228000	0.000002000	-2.707679000
H	3.675527000	-1.889700000	-2.209953000
H	4.308322000	-3.771161000	-0.689006000
H	3.025498000	-4.083498000	1.438229000
H	1.163641000	-2.511439000	1.959558000
H	-0.911910000	-1.056244000	-3.166632000
H	0.276946000	-2.002032000	-2.295201000
H	0.276948000	2.002037000	-2.295196000
H	-0.911907000	1.056252000	-3.166631000
H	-2.580177000	-2.954478000	-2.727985000
H	-4.203595000	-3.895728000	-1.084377000
H	-4.025016000	-3.191417000	1.317283000
H	-2.251519000	-1.571686000	1.954601000

H	-2.251514000	1.571689000	1.954604000
H	-4.025017000	3.191415000	1.317286000
H	-4.203601000	3.895724000	-1.084374000
H	-2.580182000	2.954478000	-2.727983000
H	-0.562740000	-0.888277000	5.337762000
H	-0.562677000	0.888295000	5.337765000
H	0.975943000	-0.000044000	5.286576000

Table S 31: Optimized Cartesian xyz coordinates of ^{3}**A'$_2$** calculated using UB3LYP/BS1 in Gaussian.

C	1.596366000	-2.340376000	-1.464266000
C	2.655152000	-3.221976000	-1.279321000
C	3.462283000	-3.076495000	-0.156161000
C	3.183312000	-2.051592000	0.747923000
C	2.112568000	-1.206854000	0.493353000
N	1.328165000	-1.347696000	-0.602088000
C	1.745591000	-0.015863000	1.374767000
C	2.159545000	1.236455000	0.610634000
C	3.235494000	2.039565000	0.962896000
C	3.546496000	3.129520000	0.149106000
C	2.766895000	3.378402000	-0.976108000
C	1.699433000	2.529688000	-1.251752000
N	1.405006000	1.476624000	-0.478165000
N	0.256698000	-0.009703000	1.528995000
C	-0.345336000	1.234519000	2.068528000
C	-0.298938000	-1.240878000	2.136187000
C	-1.485559000	-1.796991000	1.365631000
C	-1.486119000	1.761220000	1.210468000
C	-2.414461000	2.650465000	1.744762000
C	-3.410020000	3.176477000	0.928889000
C	-3.454678000	2.789571000	-0.407612000
C	-2.506754000	1.890482000	-0.871029000
N	-1.536270000	1.382667000	-0.087678000
N	-1.575609000	-1.478668000	0.062941000
C	-2.569882000	-2.011345000	-0.668369000
C	-3.507545000	-2.884108000	-0.136167000
C	-3.422465000	-3.207493000	1.215997000
C	-2.398918000	-2.652387000	1.976817000
H	0.940151000	-2.422614000	-2.321145000
H	2.835291000	-4.005859000	-2.003642000
H	4.291930000	-3.750759000	0.019244000
H	3.786944000	-1.915097000	1.636997000
H	2.273529000	-0.070120000	2.332297000
H	3.816964000	1.823368000	1.851079000
H	4.380972000	3.775228000	0.395390000
H	2.974980000	4.215797000	-1.629716000
H	1.061170000	2.692524000	-2.112257000
H	-0.697868000	1.080524000	3.092301000
H	0.421903000	2.010219000	2.124341000
H	0.478614000	-2.008664000	2.151288000
H	-0.578631000	-1.067016000	3.179414000
H	-2.354290000	2.927512000	2.790627000

H	-4.138792000	3.870698000	1.329671000
H	-4.212850000	3.169076000	-1.080359000
H	-2.511552000	1.553501000	-1.899120000
H	-2.607419000	-1.720547000	-1.711716000
H	-4.288580000	-3.291148000	-0.765343000
H	-4.142755000	-3.877077000	1.670610000
H	-2.306261000	-2.881879000	3.031922000
Fe	-0.177553000	-0.002525000	-0.616482000
N	-0.501949000	0.012724000	-2.741231000
C	-0.576011000	0.031047000	-3.891560000
C	-0.669317000	0.053266000	-5.340040000
H	-1.162825000	0.970339000	-5.669714000
H	-1.248919000	-0.805296000	-5.687373000
H	0.330138000	0.007957000	-5.778665000

Table S 32: Optimized Cartesian xyz coordinates of ^{5}**A''$_2$** calculated using UB3LYP/BS1 in Gaussian.

C	1.943459000	-2.500869000	-1.130425000
C	3.009321000	-3.322538000	-0.779945000
C	3.681571000	-3.068847000	0.411280000
C	3.266916000	-2.003171000	1.209914000
C	2.196635000	-1.226556000	0.783856000
N	1.544975000	-1.471535000	-0.369870000
C	1.710967000	0.003029000	1.550065000
C	2.194230000	1.231999000	0.781349000
C	3.262977000	2.011580000	1.205839000
C	3.675557000	3.076435000	0.405037000
C	3.002835000	3.326372000	-0.786715000
C	1.938596000	2.501897000	-1.135533000
N	1.542114000	1.473339000	-0.372886000
N	0.215824000	0.001591000	1.578966000
C	-0.381323000	1.236558000	2.147169000
C	-0.378906000	-1.233384000	2.149680000
C	-1.544162000	-1.782686000	1.342066000
C	-1.547619000	1.781961000	1.338418000
C	-2.515250000	2.578849000	1.945325000
C	-3.517255000	3.146035000	1.164206000
C	-3.528366000	2.895625000	-0.205742000
C	-2.539664000	2.079578000	-0.734946000
N	-1.566431000	1.532295000	0.014992000
N	-1.563461000	-1.535783000	0.018128000
C	-2.535647000	-2.086478000	-0.730669000
C	-3.522778000	-2.903329000	-0.199774000
C	-3.511178000	-3.150893000	1.170686000
C	-2.510258000	-2.580177000	1.950624000
H	1.388506000	-2.668555000	-2.046358000
H	3.298796000	-4.141009000	-1.426467000
H	4.513007000	-3.691770000	0.719105000
H	3.766400000	-1.784555000	2.146130000
H	2.145879000	0.004479000	2.555470000
H	3.762869000	1.795857000	2.142507000
H	4.505763000	3.701615000	0.711604000

H	3.290719000	4.144086000	-1.434904000
H	1.383336000	2.666622000	-2.051818000
H	-0.695346000	1.073799000	3.182497000
H	0.385725000	2.014085000	2.179165000
H	0.389653000	-2.009351000	2.183221000
H	-0.693218000	-1.069148000	3.184688000
H	-2.481659000	2.755803000	3.013864000
H	-4.277772000	3.770561000	1.617706000
H	-4.289444000	3.316127000	-0.850271000
H	-2.516475000	1.847377000	-1.793287000
H	-2.512910000	-1.856415000	-1.789487000
H	-4.283051000	-3.326618000	-0.843428000
H	-4.270492000	-3.775942000	1.625481000
H	-2.476320000	-2.754864000	3.019525000
Fe	-0.134456000	-0.001015000	-0.634060000
N	-0.376636000	-0.003419000	-2.762228000
C	-0.438008000	-0.004652000	-3.913264000
C	-0.513974000	-0.006203000	-5.362011000
H	-1.049343000	0.881738000	-5.706422000
H	-1.047718000	-0.895822000	-5.704608000
H	0.491306000	-0.005718000	-5.789425000

Table S 33: Optimized Cartesian xyz coordinates of $^{1}\mathbf{G_2}$ calculated using UB3LYP/BS1 in Gaussian.

C	-1.713680000	-2.356666000	1.338849000
C	-2.748724000	-3.244294000	1.060990000
C	-3.466327000	-3.099080000	-0.123039000
C	-3.131501000	-2.065171000	-0.998982000
C	-2.092077000	-1.216077000	-0.648606000
N	-1.395471000	-1.360588000	0.500917000
C	-1.637148000	0.000001000	-1.447273000
C	-2.092075000	1.216080000	-0.648606000
C	-3.131497000	2.065177000	-0.998982000
C	-3.466319000	3.099087000	-0.123040000
C	-2.748716000	3.244300000	1.060989000
C	-1.713675000	2.356668000	1.338849000
N	-1.395469000	1.360589000	0.500917000
N	-0.125730000	0.000000000	-1.420913000
C	0.513042000	1.242690000	-1.948281000
C	0.513040000	-1.242692000	-1.948281000
C	1.560794000	-1.784566000	-0.989842000
C	1.560798000	1.784562000	-0.989842000
C	2.546454000	2.681535000	-1.385629000
C	3.415208000	3.191926000	-0.422649000
C	3.271911000	2.788513000	0.902065000
C	2.271700000	1.878285000	1.223678000
N	1.438549000	1.389683000	0.291769000
N	1.438547000	-1.389684000	0.291769000
C	2.271698000	-1.878287000	1.223678000
C	3.271906000	-2.788517000	0.902066000
C	3.415200000	-3.191934000	-0.422647000
C	2.546447000	-2.681543000	-1.385627000

Fe	0.053677000	0.000000000	0.605962000
O	0.382738000	0.000001000	2.515125000
H	-1.116095000	-2.417691000	2.240399000
H	-2.982254000	-4.034010000	1.763659000
H	-4.272266000	-3.781026000	-0.366009000
H	-3.666184000	-1.926194000	-1.930944000
H	-3.666179000	1.926201000	-1.930945000
H	-4.272256000	3.781036000	-0.366011000
H	-2.982244000	4.034017000	1.763657000
H	-1.116090000	2.417692000	2.240399000
H	0.945990000	1.067771000	-2.937238000
H	-0.259424000	2.004599000	-2.075820000
H	-0.259427000	-2.004598000	-2.075821000
H	0.945988000	-1.067773000	-2.937238000
H	2.632033000	2.977613000	-2.424604000
H	4.192114000	3.892232000	-0.705678000
H	3.927023000	3.165209000	1.677290000
H	2.085605000	1.502728000	2.224916000
H	2.085604000	-1.502727000	2.224915000
H	3.927018000	-3.165213000	1.677291000
H	4.192104000	-3.892243000	-0.705675000
H	2.632025000	-2.977624000	-2.424602000
H	-0.394276000	-0.000002000	3.080240000
H	-2.058041000	0.000001000	-2.458423000

Table S 34: Optimized Cartesian xyz coordinates of $^{3}\mathbf{G_2}$ calculated using UB3LYP/BS1 in Gaussian.

C	-1.458794000	-2.489728000	1.533591000
C	-2.350584000	-3.521462000	1.257400000
C	-2.991387000	-3.550767000	0.022248000
C	-2.737812000	-2.534398000	-0.900708000
C	-1.843685000	-1.532195000	-0.552844000
N	-1.204679000	-1.525592000	0.638279000
C	-1.558297000	-0.295562000	-1.408028000
C	-2.355525000	0.847704000	-0.791707000
C	-3.534136000	1.342357000	-1.338984000
C	-4.225388000	2.327733000	-0.634112000
C	-3.713879000	2.777530000	0.579166000
C	-2.518097000	2.232661000	1.042831000
N	-1.853014000	1.291084000	0.368786000
N	-0.085916000	-0.003067000	-1.299741000
C	0.346815000	1.355708000	-1.731382000
C	0.727737000	-1.078166000	-1.931818000
C	1.966932000	-1.438003000	-1.134753000
C	1.303621000	2.004031000	-0.743905000
C	2.144752000	3.039049000	-1.140306000
C	2.958935000	3.658091000	-0.196618000
C	2.914297000	3.210929000	1.120689000
C	2.062408000	2.163893000	1.441283000
N	1.263065000	1.569958000	0.537158000
N	1.855064000	-1.296531000	0.190165000
C	2.859629000	-1.697016000	0.978284000

C	4.026672000	-2.256505000	0.467679000
C	4.154195000	-2.385767000	-0.912924000
C	3.107483000	-1.968608000	-1.732256000
Fe	0.076677000	-0.018556000	0.827991000
O	0.144679000	-0.255445000	2.680815000
H	-0.929467000	-2.385519000	2.473980000
H	-2.534438000	-4.285952000	2.001501000
H	-3.682580000	-4.348588000	-0.221784000
H	-3.230322000	-2.521772000	-1.865736000
H	-3.905956000	0.968513000	-2.285750000
H	-5.148581000	2.735734000	-1.028363000
H	-4.225373000	3.536389000	1.157678000
H	-2.078579000	2.551978000	1.981435000
H	0.806569000	1.318609000	-2.723764000
H	-0.532184000	1.996426000	-1.819341000
H	0.108992000	-1.977060000	-1.990845000
H	0.989317000	-0.807068000	-2.959760000
H	2.159647000	3.355503000	-2.176681000
H	3.618731000	4.467034000	-0.486161000
H	3.533114000	3.655824000	1.889373000
H	2.010082000	1.768370000	2.447412000
H	2.702677000	-1.559332000	2.042775000
H	4.815484000	-2.575346000	1.137290000
H	5.054227000	-2.805252000	-1.346853000
H	3.173795000	-2.061055000	-2.810253000
H	0.477778000	0.439342000	3.254936000
H	-1.870944000	-0.460793000	-2.444860000

Table S 35: Optimized Cartesian xyz coordinates of $^{5}\mathbf{G_2}$ calculated using UB3LYP/BS1 in Gaussian.

C	1.843799000	2.476334000	1.483111000
C	2.902481000	3.306909000	1.128663000
C	3.562035000	3.070737000	-0.073673000
C	3.143653000	2.011823000	-0.878041000
C	2.081629000	1.225317000	-0.445013000
N	1.442951000	1.456181000	0.714605000
C	1.587904000	-0.000294000	-1.212163000
C	2.081003000	-1.226335000	-0.445297000
C	3.142630000	-2.013280000	-0.878501000
C	3.560467000	-3.072594000	-0.074377000
C	2.900785000	-3.308711000	1.127900000
C	1.842523000	-2.477680000	1.482534000
N	1.442199000	-1.457144000	0.714262000
N	0.100518000	0.000088000	-1.232996000
C	-0.503323000	-1.230022000	-1.788347000
C	-0.502696000	1.230637000	-1.788055000
C	-1.675226000	1.756288000	-0.973665000
C	-1.676116000	-1.755278000	-0.974079000
C	-2.668443000	-2.537967000	-1.560903000
C	-3.668078000	-3.074100000	-0.754065000
C	-3.649807000	-2.806251000	0.612362000
C	-2.635328000	-2.002894000	1.118740000

N	-1.668921000	-1.492120000	0.339850000
N	-1.668156000	1.492819000	0.340203000
C	-2.634305000	2.003890000	1.119218000
C	-3.648389000	2.807868000	0.613032000
C	-3.666534000	3.076044000	-0.753333000
C	-2.667169000	2.539605000	-1.560301000
Fe	-0.231928000	-0.000110000	1.139725000
O	-0.794846000	-0.000173000	2.928805000
H	1.293187000	2.623472000	2.405632000
H	3.195343000	4.119538000	1.781191000
H	4.385980000	3.701987000	-0.384674000
H	3.631620000	1.803491000	-1.822883000
H	3.630710000	-1.804976000	-1.823291000
H	4.384093000	-3.704191000	-0.385519000
H	3.193228000	-4.121641000	1.780240000
H	1.291829000	-2.624752000	2.405017000
H	-0.813886000	-1.076962000	-2.828280000
H	0.256364000	-2.015133000	-1.808188000
H	0.257389000	2.015367000	-1.807701000
H	-0.813329000	1.077985000	-2.828027000
H	-2.656607000	-2.725754000	-2.628427000
H	-4.450217000	-3.686981000	-1.186534000
H	-4.409557000	-3.202145000	1.274224000
H	-2.570085000	-1.736668000	2.168479000
H	-2.569188000	1.737386000	2.168894000
H	-4.407940000	3.203984000	1.274989000
H	-4.448372000	3.689413000	-1.185655000
H	-2.655244000	2.727635000	-2.627781000
H	-0.293057000	-0.000408000	3.745074000
H	2.024232000	-0.000288000	-2.219148000

Table S 36: Optimized Cartesian xyz coordinates of $^{1}\mathbf{A_2}$ calculated using UB3LYP/BS1 in Gaussian.

C	-1.757246000	-2.408765000	1.312254000
C	-2.805797000	-3.274094000	1.018932000
C	-3.517828000	-3.092954000	-0.162660000
C	-3.157761000	-2.050447000	-1.017622000
C	-2.106607000	-1.225127000	-0.647745000
N	-1.415549000	-1.395133000	0.502537000
C	-1.628835000	-0.016521000	-1.437825000
C	-2.086702000	1.213033000	-0.669162000
C	-3.115773000	2.057661000	-1.057137000
C	-3.452730000	3.123354000	-0.221469000
C	-2.737839000	3.310339000	0.957594000
C	-1.710721000	2.426122000	1.269728000
N	-1.397069000	1.386265000	0.481184000
N	-0.115347000	-0.028910000	-1.373204000
C	0.529055000	1.201854000	-1.937104000
C	0.499381000	-1.285798000	-1.907262000
C	1.565322000	-1.814461000	-0.972696000
C	1.565990000	1.769442000	-0.990119000
C	2.535596000	2.668734000	-1.420197000

C	3.406905000	3.222901000	-0.486931000
C	3.281032000	2.857932000	0.850729000
C	2.298491000	1.943687000	1.202963000
N	1.455300000	1.403665000	0.304757000
N	1.486960000	-1.408262000	0.313505000
C	2.387948000	-1.890114000	1.188234000
C	3.377706000	-2.794943000	0.834829000
C	3.456063000	-3.212612000	-0.490948000
C	2.538751000	-2.708113000	-1.407158000
Fe	0.064976000	-0.009043000	0.609285000
O	0.114637000	0.078766000	2.710346000
H	-1.170507000	-2.529033000	2.214728000
H	-3.050889000	-4.074634000	1.705064000
H	-4.335513000	-3.755054000	-0.420909000
H	-3.683213000	-1.888170000	-1.950943000
H	-3.641310000	1.893048000	-1.989995000
H	-4.253446000	3.800323000	-0.493964000
H	-2.962781000	4.131098000	1.626525000
H	-1.113895000	2.554972000	2.164988000
H	0.968259000	0.991357000	-2.914566000
H	-0.241924000	1.958654000	-2.098337000
H	-0.280529000	-2.043507000	-2.007787000
H	0.906341000	-1.121833000	-2.907513000
H	2.604449000	2.934577000	-2.468368000
H	4.169254000	3.926549000	-0.798904000
H	3.934039000	3.267614000	1.610517000
H	2.166196000	1.631189000	2.230848000
H	2.321274000	-1.529683000	2.207582000
H	4.074094000	-3.153133000	1.581956000
H	4.220918000	-3.911722000	-0.806821000
H	2.576217000	-3.004973000	-2.448546000
H	0.640140000	-0.490255000	3.285237000
H	-0.729242000	0.209884000	3.160456000
H	-2.010874000	-0.022309000	-2.462237000

Table S 37: Optimized Cartesian xyz coordinates of $^3\mathbf{A_2}$ calculated using UB3LYP/BS1 in Gaussian.

C	-1.575797000	-2.508826000	1.326549000
C	-2.522870000	-3.473906000	1.004509000
C	-3.207868000	-3.366020000	-0.201533000
C	-2.921103000	-2.296256000	-1.049779000
C	-1.968442000	-1.367022000	-0.656956000
N	-1.309429000	-1.467767000	0.521458000
C	-1.604515000	-0.126983000	-1.470650000
C	-2.171956000	1.066572000	-0.708301000
C	-3.254144000	1.820017000	-1.140361000
C	-3.715339000	2.856019000	-0.327372000
C	-3.071747000	3.106242000	0.880578000
C	-1.987044000	2.313386000	1.238910000
N	-1.550912000	1.307467000	0.465902000
N	-0.114000000	-0.007180000	-1.491171000
C	0.445635000	1.283128000	-1.948357000

C	0.623677000	-1.188763000	-1.984025000
C	1.705653000	-1.665881000	-1.022809000
C	1.437703000	1.888286000	-0.962299000
C	2.346876000	2.853924000	-1.385716000
C	3.195291000	3.452539000	-0.460829000
C	3.115966000	3.061498000	0.873088000
C	2.197605000	2.084159000	1.222548000
N	1.370648000	1.503214000	0.332738000
N	1.592511000	-1.324458000	0.276443000
C	2.493160000	-1.801786000	1.155926000
C	3.539119000	-2.631190000	0.782377000
C	3.668715000	-2.973998000	-0.561339000
C	2.740448000	-2.481964000	-1.472431000
Fe	0.067945000	0.007237000	0.708069000
O	0.011855000	0.024320000	3.010165000
H	-1.008234000	-2.565351000	2.247558000
H	-2.711007000	-4.292311000	1.687492000
H	-3.947716000	-4.105380000	-0.483959000
H	-3.427352000	-2.190509000	-2.001630000
H	-3.726578000	1.606201000	-2.091497000
H	-4.559375000	3.460310000	-0.637780000
H	-3.397297000	3.903106000	1.536865000
H	-1.449892000	2.481019000	2.164699000
H	0.925065000	1.173474000	-2.925527000
H	-0.367984000	1.998908000	-2.089065000
H	-0.078852000	-2.012801000	-2.132517000
H	1.067278000	-0.987874000	-2.963666000
H	2.385407000	3.134245000	-2.431778000
H	3.906446000	4.206874000	-0.775379000
H	3.756083000	3.496890000	1.629443000
H	2.113921000	1.747288000	2.247892000
H	2.360816000	-1.505692000	2.189412000
H	4.236742000	-2.991308000	1.527422000
H	4.479005000	-3.611810000	-0.893625000
H	2.813379000	-2.730941000	-2.524648000
H	0.657870000	0.231976000	3.696740000
H	-2.047277000	-0.184512000	-2.470415000
H	-0.817725000	-0.142515000	3.474889000

Table S 38: Optimized Cartesian xyz coordinates of $^{5}\mathbf{A_2}$ calculated using UB3LYP/BS1 in Gaussian.

C	-1.993118000	-2.510708000	1.251294000
C	-3.026820000	-3.337283000	0.824863000
C	-3.616279000	-3.082924000	-0.409341000
C	-3.152109000	-2.013210000	-1.174958000
C	-2.119939000	-1.230464000	-0.673299000
N	-1.551062000	-1.473149000	0.525067000
C	-1.585297000	-0.000013000	-1.405564000
C	-2.119952000	1.230434000	-0.673302000
C	-3.152124000	2.013172000	-1.174971000
C	-3.616308000	3.082885000	-0.409361000
C	-3.026860000	3.337252000	0.824846000

C	-1.993155000	2.510684000	1.251287000
N	-1.551085000	1.473126000	0.525068000
N	-0.089049000	-0.000007000	-1.334810000
C	0.547802000	1.236035000	-1.857099000
C	0.547815000	-1.236044000	-1.857094000
C	1.644327000	-1.790521000	-0.960145000
C	1.644294000	1.790540000	-0.960141000
C	2.646066000	2.597261000	-1.494210000
C	3.578306000	3.180712000	-0.642082000
C	3.485602000	2.938918000	0.726373000
C	2.469612000	2.112616000	1.181632000
N	1.565120000	1.544472000	0.362987000
N	1.565154000	-1.544461000	0.362985000
C	2.469668000	-2.112585000	1.181620000
C	3.485679000	-2.938856000	0.726351000
C	3.578380000	-3.180640000	-0.642106000
C	2.646119000	-2.597211000	-1.494224000
Fe	0.100204000	-0.000006000	0.872791000
O	0.085582000	-0.000007000	3.071689000
H	-1.498610000	-2.684443000	2.200434000
H	-3.354354000	-4.160502000	1.446761000
H	-4.420486000	-3.709622000	-0.776215000
H	-3.584719000	-1.796711000	-2.144366000
H	-3.584726000	1.796667000	-2.144382000
H	-4.420516000	3.709575000	-0.776242000
H	-3.354404000	4.160470000	1.446740000
H	-1.498654000	2.684428000	2.200430000
H	0.944317000	1.070754000	-2.863041000
H	-0.217053000	2.010449000	-1.953689000
H	-0.217030000	-2.010470000	-1.953664000
H	0.944312000	-1.070769000	-2.863044000
H	2.691694000	2.770360000	-2.562933000
H	4.363612000	3.812828000	-1.039308000
H	4.186801000	3.374781000	1.426236000
H	2.367077000	1.896591000	2.238811000
H	2.367136000	-1.896568000	2.238801000
H	4.186894000	-3.374703000	1.426208000
H	4.363701000	-3.812733000	-1.039340000
H	2.691745000	-2.770304000	-2.562948000
H	0.811266000	-0.000072000	3.707972000
H	-0.730798000	0.000043000	3.587266000
H	-1.950957000	-0.000016000	-2.437867000

Table S 39: Optimized Cartesian xyz coordinates of $^{1}\mathbf{B_2}$ calculated using UB3LYP/BS1 in Gaussian.

C	1.759158000	2.362619000	0.967138000
C	2.769793000	3.245881000	0.598141000
C	3.383485000	3.095079000	-0.641065000
C	2.971985000	2.059366000	-1.481410000
C	1.962226000	1.217196000	-1.040901000
N	1.363763000	1.367913000	0.161455000
C	1.444464000	-0.000004000	-1.796856000

C	1.962222000	-1.217205000	-1.040899000
C	2.971979000	-2.059379000	-1.481406000
C	3.383475000	-3.095091000	-0.641058000
C	2.769783000	-3.245888000	0.598149000
C	1.759150000	-2.362623000	0.967143000
N	1.363758000	-1.367918000	0.161457000
N	-0.058214000	-0.000001000	-1.648476000
C	-0.732724000	-1.241939000	-2.130395000
C	-0.732720000	1.241939000	-2.130396000
C	-1.705943000	1.786286000	-1.100232000
C	-1.705950000	-1.786282000	-1.100232000
C	-2.715930000	-2.682842000	-1.430624000
C	-3.512149000	-3.203953000	-0.413044000
C	-3.273629000	-2.810803000	0.900687000
C	-2.255392000	-1.900097000	1.155192000
N	-1.490757000	-1.398454000	0.171831000
N	-1.490752000	1.398457000	0.171831000
C	-2.255385000	1.900104000	1.155193000
C	-3.273618000	2.810814000	0.900687000
C	-3.512136000	3.203965000	-0.413044000
C	-2.715919000	2.682851000	-1.430624000
1	1.778649000	-0.000006000	-2.839606000
Fe	-0.082056000	-0.000001000	0.384406000
O	-0.339481000	-0.000005000	2.299351000
Cl	1.027229000	0.000007000	3.364228000
1	1.258344000	2.425090000	1.924280000
1	3.066941000	4.033344000	1.278758000
1	4.169967000	3.771737000	-0.953004000
1	3.426649000	1.912665000	-2.453717000
1	3.426643000	-1.912681000	-2.453713000
1	4.169956000	-3.771752000	-0.952994000
1	3.066929000	-4.033350000	1.278768000
1	1.258336000	-2.425090000	1.924285000
1	-1.236545000	-1.065265000	-3.084690000
1	0.028976000	-2.002994000	-2.314904000
1	0.028983000	2.002991000	-2.314906000
1	-1.236542000	1.065266000	-3.084690000
1	-2.874318000	-2.970829000	-2.463260000
1	-4.305506000	-3.904531000	-0.644961000
1	-3.868187000	-3.195431000	1.719459000
1	-2.008428000	-1.538310000	2.147427000
1	-2.008422000	1.538314000	2.147427000
1	-3.868175000	3.195445000	1.719460000
1	-4.305490000	3.904547000	-0.644961000
1	-2.874306000	2.970839000	-2.463260000

Table S 40: Optimized Cartesian xyz coordinates of $^{3}\mathbf{B_2}$ calculated using UB3LYP/BS1 in Gaussian.

C	-1.569245000	-2.562312000	1.030398000
C	-2.460206000	-3.549463000	0.622533000
C	-3.052760000	-3.449851000	-0.631125000
C	-2.733317000	-2.361172000	-1.441971000

C	-1.839538000	-1.412499000	-0.965716000
N	-1.264265000	-1.508536000	0.257182000
C	-1.462048000	-0.152313000	-1.740304000
C	-2.215579000	1.005262000	-1.095468000
C	-3.313409000	1.632237000	-1.672707000
C	-3.966452000	2.622660000	-0.937906000
C	-3.499227000	2.945313000	0.332391000
C	-2.383948000	2.271719000	0.826820000
N	-1.758395000	1.324291000	0.123069000
N	0.017342000	0.049398000	-1.560995000
C	0.540889000	1.386230000	-1.961396000
C	0.812973000	-1.062229000	-2.152247000
C	1.999600000	-1.463981000	-1.295115000
C	1.470094000	1.979798000	-0.916872000
C	2.370711000	2.990880000	-1.234611000
C	3.132689000	3.563003000	-0.219114000
C	2.971556000	3.103581000	1.084712000
C	2.064692000	2.080632000	1.328358000
N	1.331212000	1.528377000	0.346883000
N	1.833480000	-1.320316000	0.025191000
C	2.798228000	-1.734724000	0.855838000
C	3.973783000	-2.319292000	0.395704000
C	4.154236000	-2.459705000	-0.977533000
C	3.152351000	-2.022033000	-1.841126000
1	-1.744579000	-0.250959000	-2.794139000
Fe	0.067592000	-0.013982000	0.576127000
O	0.314152000	0.100815000	2.416915000
Cl	-0.801749000	-0.495740000	3.609558000
1	-1.091940000	-2.591164000	2.000762000
1	-2.678664000	-4.377897000	1.284025000
1	-3.747105000	-4.205944000	-0.977447000
1	-3.170191000	-2.252601000	-2.427407000
1	-3.652644000	1.357060000	-2.664450000
1	-4.826991000	3.132957000	-1.354123000
1	-3.985035000	3.703532000	0.933353000
1	-1.979058000	2.483766000	1.810193000
1	1.047179000	1.328371000	-2.929594000
1	-0.301050000	2.069539000	-2.089993000
1	0.162014000	-1.935149000	-2.241140000
1	1.136429000	-0.805770000	-3.166084000
1	2.469890000	3.327594000	-2.259852000
1	3.839320000	4.352864000	-0.444443000
1	3.541468000	3.522594000	1.904066000
1	1.885459000	1.667629000	2.314759000
1	2.607988000	-1.580901000	1.912549000
1	4.729071000	-2.646709000	1.098869000
1	5.062136000	-2.900227000	-1.372397000
1	3.263350000	-2.116917000	-2.915176000

Table S 41: Optimized Cartesian xyz coordinates of $^{5}\mathbf{B_2}$ calculated using UB3LYP/BS1 in Gaussian.

C	-2.044392000	-2.453737000	0.897347000

C	-3.057208000	-3.281331000	0.421219000
C	-3.556767000	-3.057956000	-0.857644000
C	-3.034587000	-2.010614000	-1.616106000
C	-2.030626000	-1.224904000	-1.062296000
N	-1.541205000	-1.448939000	0.169677000
C	-1.447211000	0.000024000	-1.766506000
C	-2.030614000	1.224945000	-1.062276000
C	-3.034567000	2.010675000	-1.616072000
C	-3.556737000	3.058009000	-0.857591000
C	-3.057176000	3.281356000	0.421275000
C	-2.044369000	2.453743000	0.897390000
N	-1.541192000	1.448953000	0.169702000
N	0.034406000	0.000015000	-1.606480000
C	0.700569000	1.231767000	-2.085304000
C	0.700557000	-1.231737000	-2.085322000
C	1.761338000	-1.762448000	-1.132566000
C	1.761357000	1.762453000	-1.132541000
C	2.809116000	2.557404000	-1.592436000
C	3.697050000	3.102159000	-0.669237000
C	3.515186000	2.831556000	0.684498000
C	2.456359000	2.015322000	1.062360000
N	1.598054000	1.494406000	0.170940000
N	1.598038000	-1.494418000	0.170919000
C	2.456339000	-2.015352000	1.062330000
C	3.515159000	-2.831590000	0.684457000
C	3.697021000	-3.102176000	-0.669281000
C	2.809091000	-2.557400000	-1.592473000
1	-1.755785000	0.000034000	-2.819086000
Fe	0.068971000	-0.000002000	0.747147000
O	0.441488000	0.000010000	2.632248000
Cl	-0.874459000	-0.000080000	3.735771000
1	-1.623493000	-2.585668000	1.887730000
1	-3.439087000	-4.080711000	1.043251000
1	-4.340010000	-3.688154000	-1.261975000
1	-3.401460000	-1.809715000	-2.615703000
1	-3.401442000	1.809798000	-2.615673000
1	-4.339973000	3.688222000	-1.261911000
1	-3.439047000	4.080729000	1.043322000
1	-1.623468000	2.585653000	1.887775000
1	1.138052000	1.076846000	-3.077837000
1	-0.053382000	2.014201000	-2.200862000
1	-0.053402000	-2.014162000	-2.200892000
1	1.138042000	-1.076806000	-3.077852000
1	2.924581000	2.749110000	-2.653004000
1	4.518770000	3.725103000	-1.002431000
1	4.182740000	3.235333000	1.434952000
1	2.268485000	1.752928000	2.098142000
1	2.268469000	-1.752970000	2.098116000
1	4.182711000	-3.235382000	1.434905000
1	4.518736000	-3.725121000	-1.002485000
1	2.924554000	-2.749092000	-2.653044000

Table S 42: Optimized Cartesian xyz coordinates of $^{1}\mathbf{E_2}$ calculated using UB3LYP/BS1 in Gaussian.

C	-2.740747000	3.213708000	-1.090058000
C	-3.449171000	3.078385000	0.099414000
C	-3.104734000	2.061249000	0.994485000
C	-2.058878000	1.218206000	0.658639000
N	-1.367927000	1.367053000	-0.493500000
C	-1.601250000	0.000000000	1.451683000
C	-2.058879000	-1.218206000	0.658639000
C	-3.104734000	-2.061249000	0.994485000
C	-3.449171000	-3.078385000	0.099414000
C	-2.740748000	-3.213708000	-1.090058000
C	-1.694867000	-2.335602000	-1.358478000
N	-1.367928000	-1.367053000	-0.493500000
N	-0.087674000	0.000000000	1.410560000
C	0.542546000	-1.246651000	1.947521000
C	0.542546000	1.246651000	1.947521000
C	1.552058000	1.815529000	0.973118000
C	1.552058000	-1.815529000	0.973118000
C	2.510221000	-2.746833000	1.354923000
C	3.351957000	-3.288650000	0.386989000
C	3.211269000	-2.884129000	-0.938191000
C	2.243993000	-1.941618000	-1.252267000
N	1.434652000	-1.418670000	-0.311327000
N	1.434652000	1.418670000	-0.311327000
C	2.243993000	1.941618000	-1.252267000
C	3.211269000	2.884129000	-0.938191000
C	3.351957000	3.288650000	0.386989000
C	2.510222000	2.746833000	1.354923000
C	-1.694867000	2.335602000	-1.358478000
H	-1.996752000	0.000000000	2.470231000
H	-2.989396000	3.984974000	-1.807684000
H	-4.264005000	3.753514000	0.332457000
H	-3.642614000	1.930983000	1.925581000
H	-3.642614000	-1.930983000	1.925581000
H	-4.264005000	-3.753513000	0.332457000
H	-2.989396000	-3.984974000	-1.807684000
H	-1.110076000	-2.382663000	-2.268614000
H	1.005098000	-1.054977000	2.917845000
H	-0.236483000	-1.993896000	2.115851000
H	-0.236482000	1.993896000	2.115851000
H	1.005098000	1.054977000	2.917845000
H	2.593089000	-3.045851000	2.393003000
H	4.105282000	-4.016326000	0.664341000
H	3.841661000	-3.285771000	-1.721049000
H	2.083474000	-1.587798000	-2.262557000
H	2.083475000	1.587798000	-2.262557000
H	3.841662000	3.285771000	-1.721049000
H	4.105283000	4.016325000	0.664341000
H	2.593089000	3.045850000	2.393003000
H	-1.110076000	2.382663000	-2.268614000

Fe	0.086150000	0.000000000	-0.668188000
O	0.238512000	0.000000000	-2.288161000

Table S 43: Optimized Cartesian xyz coordinates of $^{3}\mathbf{E_2}$ calculated using UB3LYP/BS1 in Gaussian.

C	2.774322000	-3.197873000	-1.088168000
C	3.469297000	-3.069894000	0.109984000
C	3.113389000	-2.058646000	1.006915000
C	2.069665000	-1.215414000	0.664896000
N	1.389553000	-1.357485000	-0.494677000
C	1.607230000	0.000000000	1.459087000
C	2.069664000	1.215414000	0.664896000
C	3.113388000	2.058646000	1.006915000
C	3.469296000	3.069895000	0.109984000
C	2.774321000	3.197874000	-1.088168000
C	1.729714000	2.320023000	-1.361658000
N	1.389553000	1.357485000	-0.494677000
N	0.096436000	0.000000000	1.409014000
C	-0.542868000	1.245979000	1.938896000
C	-0.542868000	-1.245980000	1.938896000
C	-1.567838000	-1.794267000	0.968404000
C	-1.567838000	1.794267000	0.968404000
C	-2.539186000	2.710863000	1.352170000
C	-3.394159000	3.235997000	0.386464000
C	-3.255147000	2.827795000	-0.937666000
C	-2.274334000	1.899495000	-1.253374000
N	-1.450856000	1.394966000	-0.315807000
N	-1.450856000	-1.394966000	-0.315807000
C	-2.274334000	-1.899495000	-1.253373000
C	-3.255148000	-2.827795000	-0.937666000
C	-3.394160000	-3.235997000	0.386464000
C	-2.539186000	-2.710863000	1.352171000
C	1.729715000	-2.320023000	-1.361658000
H	2.000605000	0.000000000	2.478407000
H	3.033236000	-3.962398000	-1.809323000
H	4.282879000	-3.744992000	0.347434000
H	3.641025000	-1.932384000	1.944419000
H	3.641024000	1.932384000	1.944419000
H	4.282878000	3.744993000	0.347434000
H	3.033235000	3.962399000	-1.809322000
H	1.158908000	2.363181000	-2.280511000
H	-0.993646000	1.058991000	2.915715000
H	0.230858000	2.001988000	2.091363000
H	0.230858000	-2.001988000	2.091362000
H	-0.993646000	-1.058992000	2.915715000
H	-2.621636000	3.012374000	2.389561000
H	-4.157220000	3.952952000	0.665108000
H	-3.897588000	3.214697000	-1.718088000
H	-2.119517000	1.537969000	-2.261805000
H	-2.119517000	-1.537969000	-2.261805000
H	-3.897589000	-3.214696000	-1.718088000
H	-4.157220000	-3.952951000	0.665108000

H	-2.621636000	-3.012374000	2.389561000
H	1.158909000	-2.363181000	-2.280511000
Fe	-0.075362000	0.000000000	-0.671313000
O	-0.201278000	0.000000000	-2.288153000

Table S 44: Optimized Cartesian xyz coordinates of $^{5}\mathbf{E_2}$ calculated using UB3LYP/BS1 in Gaussian.

C	2.957265000	-3.228970000	-0.998181000
C	3.586051000	-3.047106000	0.229324000
C	3.155066000	-2.023360000	1.078124000
C	2.105175000	-1.221770000	0.659524000
N	1.489735000	-1.411921000	-0.526212000
C	1.588490000	0.000001000	1.411321000
C	2.105173000	1.221772000	0.659523000
C	3.155064000	2.023364000	1.078123000
C	3.586048000	3.047109000	0.229322000
C	2.957262000	3.228973000	-0.998182000
C	1.903567000	2.388241000	-1.344739000
N	1.489734000	1.411923000	-0.526212000
N	0.077467000	0.000000000	1.304430000
C	-0.552023000	1.245857000	1.860983000
C	-0.552022000	-1.245856000	1.860983000
C	-1.621756000	-1.808169000	0.949552000
C	-1.621759000	1.808168000	0.949553000
C	-2.601570000	2.680183000	1.408113000
C	-3.489393000	3.238581000	0.490329000
C	-3.376474000	2.907919000	-0.858394000
C	-2.385994000	2.017541000	-1.245085000
N	-1.529125000	1.482366000	-0.355932000
N	-1.529122000	-1.482368000	-0.355932000
C	-2.385990000	-2.017543000	-1.245086000
C	-3.376469000	-2.907923000	-0.858396000
C	-3.489388000	-3.238586000	0.490327000
C	-2.601566000	-2.680186000	1.408112000
C	1.903570000	-2.388239000	-1.344738000
H	1.922308000	0.000002000	2.452050000
H	3.272977000	-4.006283000	-1.682140000
H	4.405412000	-3.690688000	0.527084000
H	3.631672000	-1.857591000	2.036732000
H	3.631670000	1.857596000	2.036731000
H	4.405408000	3.690693000	0.527081000
H	3.272973000	4.006285000	-1.682142000
H	1.380167000	2.482651000	-2.288545000
H	-0.953421000	1.052860000	2.857976000
H	0.226852000	2.002758000	1.977624000
H	0.226854000	-2.002757000	1.977625000
H	-0.953421000	-1.052860000	2.857976000
H	-2.667332000	2.924896000	2.461524000
H	-4.259444000	3.923201000	0.825791000
H	-4.046372000	3.324851000	-1.599268000
H	-2.253307000	1.711426000	-2.275595000
H	-2.253304000	-1.711428000	-2.275596000

H	-4.046366000	-3.324856000	-1.599270000
H	-4.259438000	-3.923207000	0.825789000
H	-2.667328000	-2.924900000	2.461523000
H	1.380170000	-2.482650000	-2.288544000
Fe	-0.111904000	0.000000000	-0.803018000
O	-0.271002000	0.000000000	-2.407426000

Table S 45: Optimized Cartesian xyz coordinates of $^2\mathbf{C_2}$ calculated using UB3LYP/BS1 in Gaussian.

C	-1.723515000	2.363058000	-1.336513000
C	-2.758511000	3.249655000	-1.056352000
C	-3.473169000	3.101245000	0.128077000
C	-3.137768000	2.067471000	1.006106000
C	-2.102837000	1.216696000	0.654404000
N	-1.412720000	1.363715000	-0.498933000
C	-1.644240000	0.000000000	1.446012000
C	-2.102838000	-1.216695000	0.654404000
C	-3.137770000	-2.067470000	1.006105000
C	-3.473171000	-3.101243000	0.128076000
C	-2.758513000	-3.249654000	-1.056353000
C	-1.723516000	-2.363057000	-1.336513000
N	-1.412721000	-1.363715000	-0.498933000
N	-0.128664000	-0.000001000	1.391787000
C	0.511537000	-1.246210000	1.933967000
C	0.511538000	1.246208000	1.933968000
C	1.567248000	1.775221000	0.985846000
C	1.567249000	-1.775221000	0.985846000
C	2.550042000	-2.672463000	1.383961000
C	3.427989000	-3.177886000	0.427250000
C	3.299289000	-2.769066000	-0.897518000
C	2.304188000	-1.858686000	-1.225656000
N	1.459798000	-1.375411000	-0.298173000
N	1.459798000	1.375410000	-0.298173000
C	2.304187000	1.858686000	-1.225656000
C	3.299286000	2.769068000	-0.897519000
C	3.427986000	3.177888000	0.427249000
C	2.550040000	2.672464000	1.383960000
Fe	0.059005000	-0.000001000	-0.634961000
O	0.305932000	-0.000001000	-2.420296000
H	-1.128636000	2.440065000	-2.238022000
H	-2.991912000	4.040000000	-1.758155000
H	-4.279534000	3.782541000	0.372240000
H	-3.670972000	1.932528000	1.939262000
H	-3.670974000	-1.932527000	1.939261000
H	-4.279537000	-3.782539000	0.372239000
H	-2.991915000	-4.039999000	-1.758156000
H	-1.128637000	-2.440065000	-2.238022000
H	0.930522000	-1.057667000	2.924249000
H	-0.260176000	-2.008985000	2.058650000
H	-0.260175000	2.008983000	2.058654000
H	0.930526000	1.057663000	2.924249000
H	2.625081000	-2.975051000	2.421574000

H	4.201449000	-3.880122000	0.714546000
H	3.961757000	-3.140794000	-1.668518000
H	2.150776000	-1.490796000	-2.232401000
H	2.150776000	1.490795000	-2.232401000
H	3.961754000	3.140796000	-1.668519000
H	4.201445000	3.880126000	0.714545000
H	2.625078000	2.975053000	2.421573000
H	-0.491354000	0.000001000	-2.964082000
H	-2.033764000	0.000000000	2.466651000

Table S 46: Optimized Cartesian xyz coordinates of $^{4}\mathbf{C_2}$ calculated using UB3LYP/BS1 in Gaussian.

C	-1.731320000	2.388266000	-1.321448000
C	-2.763624000	3.270295000	-1.020823000
C	-3.461522000	3.108126000	0.171698000
C	-3.111643000	2.066384000	1.033585000
C	-2.077361000	1.220018000	0.665525000
N	-1.403770000	1.386803000	-0.492495000
C	-1.625325000	0.000005000	1.462243000
C	-2.077369000	-1.220005000	0.665526000
C	-3.111657000	-2.066364000	1.033588000
C	-3.461542000	-3.108105000	0.171703000
C	-2.763646000	-3.270280000	-1.020818000
C	-1.731336000	-2.388258000	-1.321445000
N	-1.403779000	-1.386796000	-0.492494000
N	-0.131745000	0.000000000	1.483918000
C	0.510155000	-1.239888000	1.977475000
C	0.510162000	1.239884000	1.977476000
C	1.544380000	1.786006000	1.006168000
C	1.544369000	-1.786015000	1.006166000
C	2.512193000	-2.697499000	1.415912000
C	3.391100000	-3.228140000	0.476464000
C	3.283710000	-2.829798000	-0.853960000
C	2.307370000	-1.907816000	-1.198167000
N	1.458111000	-1.401832000	-0.282493000
N	1.458120000	1.401824000	-0.282491000
C	2.307382000	1.907805000	-1.198164000
C	3.283727000	2.829780000	-0.853956000
C	3.391120000	3.228120000	0.476468000
C	2.512210000	2.697483000	1.415915000
Fe	0.077433000	0.000000000	-0.790574000
O	0.290744000	0.000000000	-2.612401000
H	-1.153910000	2.466502000	-2.233911000
H	-3.008976000	4.065013000	-1.713523000
H	-4.267348000	3.783703000	0.433155000
H	-3.633959000	1.919032000	1.971081000
H	-3.633972000	-1.919008000	1.971084000
H	-4.267373000	-3.783676000	0.433161000
H	-3.009003000	-4.064997000	-1.713518000
H	-1.153927000	-2.466500000	-2.233908000
H	0.971746000	-1.074402000	2.954736000
H	-0.253786000	-2.006810000	2.127735000

H	-0.253774000	2.006810000	2.127736000
H	0.971752000	1.074395000	2.954738000
H	2.572289000	-2.989018000	2.457875000
H	4.149656000	-3.940091000	0.779364000
H	3.947442000	-3.217909000	-1.615723000
H	2.173332000	-1.548923000	-2.210872000
H	2.173342000	1.548913000	-2.210869000
H	3.947462000	3.217888000	-1.615719000
H	4.149680000	3.940066000	0.779369000
H	2.572308000	2.989001000	2.457879000
H	-0.185586000	-0.000004000	-3.446263000
H	-2.074353000	0.000007000	2.460104000

Table S 47: Optimized Cartesian xyz coordinates of $^{6}\mathbf{C_2}$ calculated using UB3LYP/BS1 in Gaussian.

C	-1.918170000	-2.439106000	1.300166000
C	-2.960008000	-3.282320000	0.928896000
C	-3.586018000	-3.073620000	-0.295945000
C	-3.156312000	-2.026322000	-1.113861000
C	-2.115127000	-1.224568000	-0.670611000
N	-1.507471000	-1.432639000	0.514414000
C	-1.600313000	0.000000000	-1.420180000
C	-2.115127000	1.224568000	-0.670611000
C	-3.156312000	2.026322000	-1.113861000
C	-3.586017000	3.073621000	-0.295945000
C	-2.960008000	3.282320000	0.928896000
C	-1.918170000	2.439106000	1.300166000
N	-1.507470000	1.432639000	0.514414000
N	-0.102468000	0.000000000	-1.344664000
C	0.525954000	1.238028000	-1.884804000
C	0.525953000	-1.238028000	-1.884804000
C	1.608807000	-1.795924000	-0.981092000
C	1.608807000	1.795924000	-0.981092000
C	2.588213000	2.654359000	-1.469185000
C	3.501551000	3.215238000	-0.580212000
C	3.415124000	2.899206000	0.774074000
C	2.423686000	2.023812000	1.189045000
N	1.538342000	1.485801000	0.327152000
N	1.538342000	-1.485801000	0.327153000
C	2.423686000	-2.023812000	1.189045000
C	3.415124000	-2.899206000	0.774074000
C	3.501551000	-3.215238000	-0.580211000
C	2.588213000	-2.654360000	-1.469185000
Fe	0.109089000	0.000000000	0.921649000
O	0.301787000	0.000001000	2.703100000
H	-1.397345000	-2.561854000	2.242134000
H	-3.268010000	-4.082477000	1.589589000
H	-4.397692000	-3.716560000	-0.615336000
H	-3.624388000	-1.842106000	-2.073332000
H	-3.624387000	1.842107000	-2.073332000
H	-4.397691000	3.716560000	-0.615336000
H	-3.268009000	4.082478000	1.589589000

H	-1.397345000	2.561854000	2.242134000
H	0.926919000	1.060257000	-2.886046000
H	-0.244473000	2.005024000	-1.994051000
H	-0.244474000	-2.005025000	-1.994051000
H	0.926918000	-1.060258000	-2.886046000
H	2.633009000	2.884635000	-2.527037000
H	4.270628000	3.888213000	-0.940532000
H	4.105940000	3.315207000	1.495975000
H	2.314444000	1.729914000	2.225753000
H	2.314444000	-1.729914000	2.225753000
H	4.105941000	-3.315207000	1.495975000
H	4.270628000	-3.888213000	-0.940531000
H	2.633008000	-2.884636000	-2.527037000
H	0.031217000	0.000001000	3.623872000
H	-1.965337000	0.000000000	-2.451636000

Table S 48: Optimized Cartesian xyz coordinates of $^{2}\mathbf{D_2}$ calculated using UB3LYP/BS1 in Gaussian.

C	-1.736788000	-2.381972000	0.965963000
C	-2.742852000	-3.271254000	0.601107000
C	-3.373768000	-3.112742000	-0.627959000
C	-2.981199000	-2.068107000	-1.468734000
C	-1.978010000	-1.218116000	-1.035139000
N	-1.371955000	-1.371425000	0.164058000
C	-1.464607000	0.000001000	-1.786177000
C	-1.978009000	1.218118000	-1.035139000
C	-2.981197000	2.068110000	-1.468734000
C	-3.373765000	3.112745000	-0.627958000
C	-2.742850000	3.271255000	0.601108000
C	-1.736786000	2.381973000	0.965964000
N	-1.371954000	1.371426000	0.164058000
N	0.042439000	0.000000000	-1.617222000
C	0.718512000	1.245713000	-2.114133000
C	0.718511000	-1.245713000	-2.114133000
C	1.704085000	-1.775500000	-1.095584000
C	1.704086000	1.775499000	-1.095584000
C	2.712019000	2.671608000	-1.428438000
C	3.522951000	3.181262000	-0.416878000
C	3.304244000	2.776290000	0.897143000
C	2.289678000	1.866443000	1.158672000
N	1.507594000	1.378996000	0.179277000
N	1.507593000	-1.378998000	0.179276000
C	2.289677000	-1.866444000	1.158672000
C	3.304242000	-2.776292000	0.897142000
C	3.522950000	-3.181264000	-0.416879000
C	2.712017000	-2.671609000	-1.428439000
H	-1.772785000	0.000001000	-2.834152000
26	0.078990000	0.000000000	0.418285000
O	0.269816000	0.000001000	2.206780000
Cl	-1.062911000	-0.000001000	3.287766000
H	-1.219764000	-2.463794000	1.912529000
H	-3.020085000	-4.070330000	1.276513000

H	-4.157782000	-3.794327000	-0.935800000
H	-3.446283000	-1.924605000	-2.436375000
H	-3.446282000	1.924608000	-2.436375000
H	-4.157779000	3.794330000	-0.935799000
H	-3.020081000	4.070331000	1.276514000
H	-1.219762000	2.463794000	1.912530000
H	1.206215000	1.056077000	-3.072171000
H	-0.042873000	2.007952000	-2.293288000
H	-0.042874000	-2.007952000	-2.293288000
H	1.206214000	-1.056078000	-3.072171000
H	2.857091000	2.970139000	-2.459785000
H	4.313806000	3.883110000	-0.653257000
H	3.912000000	3.150056000	1.711027000
H	2.075235000	1.504355000	2.156200000
H	2.075234000	-1.504357000	2.156200000
H	3.911998000	-3.150058000	1.711027000
H	4.313805000	-3.883111000	-0.653257000
H	2.857090000	-2.970140000	-2.459786000

Table S 49: Optimized Cartesian xyz coordinates of $^4\mathbf{D_2}$ calculated using UB3LYP/BS1 in Gaussian.

C	-1.768735000	-2.376708000	0.921038000
C	-2.774747000	-3.256717000	0.535416000
C	-3.363466000	-3.100870000	-0.714836000
C	-2.936140000	-2.065110000	-1.549671000
C	-1.936273000	-1.219417000	-1.097718000
N	-1.365268000	-1.383184000	0.115942000
C	-1.417707000	-0.000001000	-1.854506000
C	-1.936273000	1.219416000	-1.097718000
C	-2.936140000	2.065109000	-1.549672000
C	-3.363465000	3.100869000	-0.714837000
C	-2.774746000	3.256717000	0.535414000
C	-1.768734000	2.376709000	0.921036000
N	-1.365267000	1.383183000	0.115942000
N	0.074046000	-0.000001000	-1.749594000
C	0.757731000	1.241256000	-2.182411000
C	0.757732000	-1.241258000	-2.182410000
C	1.701736000	-1.786876000	-1.122110000
C	1.701736000	1.786875000	-1.122112000
C	2.700527000	2.700245000	-1.443133000
C	3.491472000	3.231775000	-0.428836000
C	3.267182000	2.832666000	0.886751000
C	2.266362000	1.908356000	1.142282000
N	1.502653000	1.400866000	0.154367000
N	1.502652000	-1.400866000	0.154368000
C	2.266362000	-1.908355000	1.142284000
C	3.267181000	-2.832666000	0.886753000
C	3.491471000	-3.231776000	-0.428834000
C	2.700527000	-2.700247000	-1.443130000
H	-1.779578000	-0.000001000	-2.886916000
26	0.081845000	0.000000000	0.511708000
O	0.148402000	0.000000000	2.378694000

Cl	-1.014808000	0.000004000	3.608476000
H	-1.282012000	-2.449646000	1.885258000
H	-3.084623000	-4.044131000	1.210336000
H	-4.145781000	-3.775556000	-1.041830000
H	-3.374802000	-1.921856000	-2.529695000
H	-3.374802000	1.921854000	-2.529696000
H	-4.145780000	3.775556000	-1.041832000
H	-3.084622000	4.044132000	1.210334000
H	-1.282011000	2.449647000	1.885257000
H	1.304020000	1.076608000	-3.114981000
H	0.009456000	2.007902000	-2.398500000
H	0.009456000	-2.007903000	-2.398499000
H	1.304020000	-1.076610000	-3.114979000
H	2.852384000	2.993293000	-2.475287000
H	4.272426000	3.945536000	-0.662719000
H	3.859067000	3.222118000	1.704952000
H	2.046302000	1.552329000	2.141187000
H	2.046301000	-1.552328000	2.141189000
H	3.859065000	-3.222118000	1.704955000
H	4.272425000	-3.945537000	-0.662716000
H	2.852383000	-2.993295000	-2.475284000

Table S 50: Optimized Cartesian xyz coordinates of $^{6}\mathbf{D_2}$ calculated using UB3LYP/BS1 in Gaussian.

C	1.943611000	2.428304000	0.924819000
C	2.965396000	3.270413000	0.498508000
C	3.517971000	3.067863000	-0.761959000
C	3.038967000	2.026217000	-1.560035000
C	2.023066000	1.224668000	-1.062076000
N	1.484241000	1.428947000	0.157049000
C	1.467347000	0.000587000	-1.784022000
C	2.023284000	-1.223700000	-1.062596000
C	3.039332000	-2.024849000	-1.560899000
C	3.518514000	-3.066763000	-0.763281000
C	2.965959000	-3.269971000	0.497087000
C	1.944020000	-2.428231000	0.923761000
N	1.484482000	-1.428610000	0.156436000
N	-0.025440000	0.000413000	-1.626147000
C	-0.685518000	-1.239015000	-2.126024000
C	-0.685752000	1.239932000	-2.125498000
C	-1.714572000	1.797656000	-1.159274000
C	-1.714294000	-1.797274000	-1.160064000
C	-2.717901000	-2.658385000	-1.590428000
C	-3.576419000	-3.222447000	-0.650108000
C	-3.412562000	-2.907508000	0.697294000
C	-2.401942000	-2.028965000	1.054826000
N	-1.570103000	-1.486914000	0.142940000
N	-1.570297000	1.486795000	0.143601000
C	-2.402165000	2.028373000	1.055743000
C	-3.412915000	2.906923000	0.698600000
C	-3.576869000	3.222374000	-0.648671000
C	-2.718313000	2.658805000	-1.589252000

H	1.774350000	0.000837000	-2.834018000
26	-0.114562000	-0.000042000	0.624284000
O	-0.187573000	-0.000353000	2.441863000
Cl	0.675669000	-0.002094000	3.877446000
H	1.482334000	2.546057000	1.898203000
H	3.314806000	4.064520000	1.145751000
H	4.311783000	3.710482000	-1.124051000
H	3.451336000	1.846093000	-2.545497000
H	3.451676000	-1.844215000	-2.546279000
H	4.312444000	-3.709081000	-1.125650000
H	3.315499000	-4.064305000	1.143982000
H	1.482768000	-2.546506000	1.897093000
H	-1.144103000	-1.062285000	-3.102322000
H	0.077720000	-2.005837000	-2.278413000
H	0.077352000	2.006940000	-2.277630000
H	-1.144359000	1.063508000	-3.101840000
H	-2.823198000	-2.889130000	-2.643870000
H	-4.362925000	-3.897740000	-0.965713000
H	-4.058345000	-3.326868000	1.457901000
H	-2.234966000	-1.738057000	2.085000000
H	-2.235094000	1.737081000	2.085793000
H	-4.058722000	3.325898000	1.459399000
H	-4.363479000	3.897688000	-0.963975000
H	-2.823682000	2.889960000	-2.642597000

Part 6: Cartesian coordinates for N4Py-Me species:

Table S 51: Optimized Cartesian xyz coordinates of ^{1}**A'**$_{1}$ calculated using UB3LYP/BS1 in Gaussian.

C	1.574231000	-2.312789000	-1.462132000
C	2.633955000	-3.196450000	-1.283679000
C	3.429436000	-3.073467000	-0.150384000
C	3.152465000	-2.062895000	0.771817000
C	2.081587000	-1.214004000	0.526095000
N	1.304027000	-1.349354000	-0.571198000
C	1.687243000	0.000001000	1.385521000
C	2.081574000	1.214012000	0.526099000
C	3.152442000	2.062916000	0.771824000
C	3.429403000	3.073492000	-0.150376000
C	2.633923000	3.196467000	-1.283672000
C	1.574211000	2.312793000	-1.462128000
N	1.304015000	1.349354000	-0.571196000
N	0.155517000	-0.000007000	1.395793000
C	-0.448486000	1.240322000	1.969075000
C	-0.448473000	-1.240345000	1.969068000
C	-1.574837000	-1.768626000	1.103525000
C	-1.574836000	1.768617000	1.103521000
C	-2.537048000	2.641322000	1.598842000
C	-3.489425000	3.160130000	0.725059000
C	-3.448101000	2.785064000	-0.613944000
C	-2.466376000	1.895923000	-1.034585000
N	-1.546838000	1.395814000	-0.191751000
N	-1.546835000	-1.395832000	-0.191750000

C	-2.466381000	-1.895932000	-1.034580000
C	-3.448118000	-2.785056000	-0.613931000
C	-3.489448000	-3.160112000	0.725074000
C	-2.537061000	-2.641312000	1.598853000
C	2.297593000	0.000000000	2.785509000
H	0.928126000	-2.338622000	-2.330864000
H	2.827028000	-3.959761000	-2.026464000
H	4.259268000	-3.749509000	0.017656000
H	3.764465000	-1.947461000	1.656134000
H	3.764440000	1.947489000	1.656143000
H	4.259226000	3.749544000	0.017667000
H	2.826990000	3.959780000	-2.026456000
H	0.928107000	2.338620000	-2.330862000
H	-0.792997000	1.072991000	2.993121000
H	0.324165000	2.010779000	2.018443000
H	0.324181000	-2.010800000	2.018405000
H	-0.792963000	-1.073033000	2.993124000
H	-2.538130000	2.912866000	2.647922000
H	-4.249235000	3.843028000	1.086022000
H	-4.167096000	3.165977000	-1.327903000
H	-2.382638000	1.558592000	-2.060707000
H	-2.382640000	-1.558609000	-2.060704000
H	-4.167120000	-3.165964000	-1.327887000
H	-4.249269000	-3.842995000	1.086043000
H	-2.538147000	-2.912848000	2.647934000
H	3.387453000	0.000006000	2.746878000
H	1.990321000	0.885007000	3.345651000
H	1.990330000	-0.885013000	3.345646000
Fe	-0.160415000	-0.000007000	-0.590887000
Cl	-0.448771000	-0.000008000	-2.902000000

Table S 52: Optimized Cartesian xyz coordinates of ^{3}**A'$_{1}$** calculated using UB3LYP/BS1 in Gaussian.

C	1.600837000	-2.330761000	-1.470535000
C	2.650444000	-3.220693000	-1.268113000
C	3.418736000	-3.094110000	-0.117292000
C	3.126552000	-2.077265000	0.793071000
C	2.064623000	-1.220785000	0.526728000
N	1.314706000	-1.366935000	-0.586395000
C	1.678729000	-0.000010000	1.397699000
C	2.064638000	1.220765000	0.526736000
C	3.126576000	2.077232000	0.793084000
C	3.418770000	3.094079000	-0.117274000
C	2.650480000	3.220674000	-1.268095000
C	1.600864000	2.330755000	-1.470521000
N	1.314723000	1.366928000	-0.586385000
N	0.176277000	-0.000002000	1.514391000
C	-0.437321000	1.236248000	2.027342000
C	-0.437332000	-1.236245000	2.027345000
C	-1.550987000	-1.785847000	1.141323000
C	-1.550968000	1.785860000	1.141316000
C	-2.493782000	2.671497000	1.656510000

C	-3.444495000	3.224769000	0.804380000
C	-3.427176000	2.873182000	-0.542159000
C	-2.468307000	1.972217000	-0.985235000
N	-1.549405000	1.440216000	-0.159353000
N	-1.549420000	-1.440205000	-0.159347000
C	-2.468329000	-1.972198000	-0.985227000
C	-3.427207000	-2.873153000	-0.542148000
C	-3.444529000	-3.224736000	0.804392000
C	-2.493809000	-2.671472000	1.656520000
C	2.362494000	-0.000017000	2.768436000
H	0.972802000	-2.356193000	-2.353536000
H	2.855817000	-3.988164000	-2.003173000
H	4.241901000	-3.772289000	0.073961000
H	3.722766000	-1.963782000	1.687683000
H	3.722789000	1.963738000	1.687696000
H	4.241943000	3.772248000	0.073983000
H	2.855861000	3.988148000	-2.003151000
H	0.972829000	2.356198000	-2.353522000
H	-0.822857000	1.092871000	3.042831000
H	0.330625000	2.010868000	2.099712000
H	0.330606000	-2.010871000	2.099713000
H	-0.822863000	-1.092862000	3.042835000
H	-2.479068000	2.924945000	2.710267000
H	-4.186187000	3.916154000	1.186575000
H	-4.145719000	3.280403000	-1.241785000
H	-2.402758000	1.649935000	-2.018408000
H	-2.402778000	-1.649919000	-2.018400000
H	-4.145755000	-3.280367000	-1.241772000
H	-4.186228000	-3.916113000	1.186589000
H	-2.479097000	-2.924918000	2.710278000
H	3.450179000	-0.000021000	2.690491000
H	2.072277000	0.883288000	3.340357000
H	2.072270000	-0.883324000	3.340352000
Fe	-0.196219000	0.000001000	-0.730735000
Cl	-0.548989000	0.000003000	-3.099443000

Table S 53: Optimized Cartesian xyz coordinates of ^{5}**A'$_1$** calculated using UB3LYP/BS1 in Gaussian.

C	1.850105000	-2.409148000	-1.400354000
C	2.919779000	-3.250426000	-1.112779000
C	3.611174000	-3.056347000	0.077074000
C	3.220879000	-2.027607000	0.934494000
C	2.142930000	-1.223837000	0.573648000
N	1.471655000	-1.427552000	-0.573765000
C	1.663014000	0.000002000	1.389509000
C	2.142915000	1.223847000	0.573649000
C	3.220851000	2.027634000	0.934499000
C	3.611132000	3.056379000	0.077079000
C	2.919738000	3.250448000	-1.112776000
C	1.850078000	2.409153000	-1.400354000
N	1.471641000	1.427551000	-0.573766000
N	0.151016000	-0.000006000	1.345029000

C	-0.464339000	1.229475000	1.896463000
C	-0.464326000	-1.229492000	1.896464000
C	-1.651169000	-1.739226000	1.095514000
C	-1.651158000	1.739228000	1.095490000
C	-2.643784000	2.507556000	1.700270000
C	-3.653282000	3.049243000	0.910063000
C	-3.642731000	2.801851000	-0.459758000
C	-2.629342000	2.009203000	-0.984133000
N	-1.653710000	1.489517000	-0.222326000
N	-1.653728000	-1.489529000	-0.222304000
C	-2.629380000	-2.009199000	-0.984095000
C	-3.642785000	-2.801815000	-0.459700000
C	-3.653328000	-3.049191000	0.910123000
C	-2.643808000	-2.507520000	1.700313000
C	2.193802000	0.000004000	2.828590000
H	1.275191000	-2.506863000	-2.314562000
H	3.197518000	-4.032684000	-1.807460000
H	4.446943000	-3.693893000	0.340284000
H	3.754519000	-1.866315000	1.860631000
H	3.754490000	1.866350000	1.860637000
H	4.446891000	3.693938000	0.340291000
H	3.197466000	4.032711000	-1.807456000
H	1.275165000	2.506860000	-2.314563000
H	-0.761605000	1.087156000	2.941186000
H	0.285672000	2.023880000	1.893350000
H	0.285683000	-2.023898000	1.893314000
H	-0.761561000	-1.087189000	2.941198000
H	-2.622627000	2.682222000	2.769759000
H	-4.434889000	3.652134000	1.357145000
H	-4.406757000	3.204990000	-1.112103000
H	-2.578771000	1.770185000	-2.040670000
H	-2.578814000	-1.770195000	-2.040635000
H	-4.406828000	-3.204941000	-1.112033000
H	-4.434946000	-3.652057000	1.357220000
H	-2.622644000	-2.682175000	2.769805000
H	3.282906000	0.000014000	2.865999000
H	1.849588000	0.884222000	3.367670000
H	1.849604000	-0.884221000	3.367666000
Fe	-0.182999000	-0.000017000	-0.962823000
Cl	-0.523772000	-0.000056000	-3.217006000

Table S 54: Optimized Cartesian xyz coordinates of $^{1}\mathbf{G_1}$ calculated using UB3LYP/BS1 in Gaussian.

C	-1.518132000	2.333239000	-1.548755000
C	-2.566114000	3.230154000	-1.373663000
C	-3.379931000	3.101682000	-0.252636000
C	-3.126177000	2.078131000	0.661641000
C	-2.064947000	1.215255000	0.419603000
N	-1.277834000	1.347930000	-0.672656000
C	-1.680017000	0.000000000	1.283414000
C	-2.064945000	-1.215257000	0.419604000
C	-3.126175000	-2.078133000	0.661641000

C	-3.379929000	-3.101684000	-0.252635000
C	-2.566111000	-3.230155000	-1.373662000
C	-1.518129000	-2.333241000	-1.548754000
N	-1.277832000	-1.347932000	-0.672655000
N	-0.147434000	0.000001000	1.313351000
C	0.463713000	-1.241444000	1.878073000
C	0.463712000	1.241446000	1.878073000
C	1.580300000	1.771849000	0.994540000
C	1.580301000	-1.771848000	0.994540000
C	2.549022000	-2.655059000	1.457339000
C	3.482415000	-3.160322000	0.553763000
C	3.418409000	-2.765448000	-0.779708000
C	2.430881000	-1.867616000	-1.168808000
N	1.535363000	-1.384526000	-0.293925000
N	1.535361000	1.384529000	-0.293925000
C	2.430880000	1.867618000	-1.168808000
C	3.418409000	2.765447000	-0.779707000
C	3.482416000	3.160321000	0.553764000
C	2.549023000	2.655059000	1.457340000
Fe	0.166182000	0.000001000	-0.685076000
O	0.610849000	0.000001000	-2.573644000
H	-0.845175000	2.374951000	-2.396732000
H	-2.735207000	4.010719000	-2.104403000
H	-4.201407000	3.788003000	-0.085767000
H	-3.746357000	1.965638000	1.540721000
H	-3.746356000	-1.965640000	1.540722000
H	-4.201405000	-3.788006000	-0.085767000
H	-2.735204000	-4.010721000	-2.104402000
H	-0.845172000	-2.374952000	-2.396730000
H	0.823238000	-1.075023000	2.897425000
H	-0.308280000	-2.011851000	1.939580000
H	-0.308282000	2.011852000	1.939577000
H	0.823235000	1.075027000	2.897425000
H	2.572181000	-2.945028000	2.501286000
H	4.247761000	-3.850169000	0.889158000
H	4.125249000	-3.138718000	-1.509851000
H	2.305430000	-1.497686000	-2.181467000
H	2.305429000	1.497688000	-2.181467000
H	4.125251000	3.138717000	-1.509850000
H	4.247763000	3.850167000	0.889160000
H	2.572182000	2.945027000	2.501286000
H	-0.128948000	-0.000002000	-3.186687000
C	-2.310512000	0.000000000	2.674770000
H	-2.008435000	0.884650000	3.238424000
H	-3.400122000	-0.000001000	2.623917000
H	-2.008434000	-0.884649000	3.238425000

Table S 55: Optimized Cartesian xyz coordinates of $^{3}\mathbf{G_1}$ calculated using UB3LYP/BS1 in Gaussian.

C	1.457766000	-2.339721000	-1.734333000
C	2.412139000	-3.334173000	-1.550592000
C	3.120191000	-3.372680000	-0.354323000

C	2.869815000	-2.404279000	0.619668000
C	1.907883000	-1.433747000	0.369371000
N	1.206701000	-1.423791000	-0.788752000
C	1.600991000	-0.236492000	1.298946000
C	2.270744000	0.969231000	0.605691000
C	3.458422000	1.561432000	1.028576000
C	4.005223000	2.585814000	0.255654000
C	3.356123000	2.983452000	-0.907836000
C	2.167569000	2.345896000	-1.251320000
N	1.640802000	1.369914000	-0.507872000
N	0.089748000	-0.032508000	1.217681000
C	-0.410385000	1.282398000	1.708180000
C	-0.658791000	-1.170160000	1.821567000
C	-1.922690000	-1.530800000	1.065777000
C	-1.444813000	1.899891000	0.780134000
C	-2.330386000	2.867517000	1.244337000
C	-3.217859000	3.465897000	0.354834000
C	-3.198943000	3.066073000	-0.978140000
C	-2.299146000	2.084198000	-1.367285000
N	-1.430446000	1.510622000	-0.515773000
N	-1.860138000	-1.367161000	-0.259502000
C	-2.883503000	-1.774475000	-1.018565000
C	-4.021658000	-2.364036000	-0.476705000
C	-4.099038000	-2.515337000	0.905225000
C	-3.031929000	-2.090737000	1.694362000
Fe	-0.158127000	0.007879000	-0.895746000
O	-0.276554000	-0.182236000	-2.757554000
H	0.872302000	-2.224308000	-2.639700000
H	2.591991000	-4.059461000	-2.333848000
H	3.864450000	-4.139932000	-0.177334000
H	3.422715000	-2.413294000	1.549239000
H	3.958298000	1.242019000	1.932331000
H	4.929140000	3.062051000	0.561992000
H	3.755722000	3.768560000	-1.537064000
H	1.618756000	2.613066000	-2.147562000
H	-0.827901000	1.191343000	2.715697000
H	0.429665000	1.975747000	1.779928000
H	-0.012825000	-2.050587000	1.790449000
H	-0.882368000	-0.975146000	2.874986000
H	-2.322557000	3.148567000	2.290954000
H	-3.913068000	4.222691000	0.697626000
H	-3.873296000	3.497452000	-1.706787000
H	-2.262944000	1.727593000	-2.388589000
H	-2.766857000	-1.617776000	-2.085595000
H	-4.826931000	-2.688659000	-1.123593000
H	-4.975671000	-2.957946000	1.363427000
H	-3.058631000	-2.200979000	2.772382000
H	-0.721417000	0.476068000	-3.297798000
C	2.085925000	-0.430264000	2.738440000
H	1.627540000	-1.306386000	3.197727000
H	3.165648000	-0.570853000	2.784481000

H	1.843384000	0.444278000	3.346022000

Table S 56: Optimized Cartesian xyz coordinates of $^5\mathbf{G_1}$ calculated using UB3LYP/BS1 in Gaussian.

C	1.832608000	-2.438817000	-1.489156000
C	2.892947000	-3.285927000	-1.187750000
C	3.585389000	-3.077903000	-0.000280000
C	3.199576000	-2.034179000	0.839672000
C	2.129018000	-1.225328000	0.464758000
N	1.462714000	-1.435087000	-0.684837000
C	1.647692000	0.000002000	1.277683000
C	2.129008000	1.225338000	0.464761000
C	3.199566000	2.034190000	0.839670000
C	3.585371000	3.077917000	-0.000281000
C	2.892922000	3.285943000	-1.187746000
C	1.832583000	2.438831000	-1.489149000
N	1.462696000	1.435098000	-0.684830000
N	0.139256000	-0.000005000	1.237328000
C	-0.480587000	1.228614000	1.782203000
C	-0.480577000	-1.228631000	1.782199000
C	-1.661211000	-1.735756000	0.968388000
C	-1.661215000	1.735745000	0.968386000
C	-2.668614000	2.497741000	1.556761000
C	-3.672423000	3.025226000	0.749120000
C	-3.643208000	2.769404000	-0.619402000
C	-2.614383000	1.985149000	-1.127096000
N	-1.644515000	1.483352000	-0.347234000
N	-1.644501000	-1.483380000	-0.347235000
C	-2.614372000	-1.985174000	-1.127095000
C	-3.643211000	-2.769409000	-0.619395000
C	-3.672436000	-3.025211000	0.749130000
C	-2.668623000	-2.497729000	1.556768000
Fe	-0.191647000	-0.000003000	-1.118924000
O	-0.730475000	-0.000002000	-2.918755000
H	1.252253000	-2.556882000	-2.397651000
H	3.161141000	-4.085237000	-1.866706000
H	4.414673000	-3.718618000	0.275341000
H	3.728962000	-1.866453000	1.767009000
H	3.728957000	1.866463000	1.767004000
H	4.414655000	3.718633000	0.275336000
H	3.161110000	4.085254000	-1.866702000
H	1.252221000	2.556898000	-2.397639000
H	-0.788743000	1.087396000	2.824425000
H	0.268188000	2.024326000	1.787298000
H	0.268201000	-2.024340000	1.787277000
H	-0.788723000	-1.087424000	2.824426000
H	-2.664968000	2.677153000	2.625771000
H	-4.466045000	3.622405000	1.182648000
H	-4.405435000	3.159639000	-1.281792000
H	-2.540245000	1.727453000	-2.178350000
H	-2.540227000	-1.727492000	-2.178352000
H	-4.405440000	-3.159641000	-1.281784000

H	-4.466069000	-3.622373000	1.182662000
H	-2.664984000	-2.677128000	2.625781000
H	-0.221497000	-0.000011000	-3.730483000
C	2.180867000	0.000002000	2.717123000
H	1.834510000	-0.883833000	3.255486000
H	3.269961000	0.000007000	2.757606000
H	1.834503000	0.883833000	3.255488000

Table S 57: Optimized Cartesian xyz coordinates of **$^1A_1$** calculated using UB3LYP/BS1 in Gaussian.

C	1.570212000	-2.379056000	-1.522906000
C	2.633668000	-3.253394000	-1.333832000
C	3.441130000	-3.088527000	-0.214004000
C	3.158534000	-2.057126000	0.682112000
C	2.082973000	-1.218156000	0.422279000
N	1.303274000	-1.376047000	-0.672820000
C	1.672250000	-0.012545000	1.282724000
C	2.060537000	1.217395000	0.447351000
C	3.112721000	2.078853000	0.728191000
C	3.370546000	3.135445000	-0.145925000
C	2.560336000	3.304515000	-1.263257000
C	1.519646000	2.407963000	-1.474106000
N	1.281657000	1.377538000	-0.647645000
N	0.134116000	-0.026948000	1.270539000
C	-0.485768000	1.201105000	1.867706000
C	-0.454405000	-1.283075000	1.836063000
C	-1.583202000	-1.801941000	0.973151000
C	-1.592513000	1.751314000	0.993002000
C	-2.549285000	2.630714000	1.488187000
C	-3.483656000	3.177352000	0.613218000
C	-3.431455000	2.825171000	-0.732735000
C	-2.457327000	1.929469000	-1.150257000
N	-1.553975000	1.396379000	-0.308481000
N	-1.576018000	-1.406662000	-0.318235000
C	-2.527997000	-1.889421000	-1.136311000
C	-3.503472000	-2.782566000	-0.718803000
C	-3.510752000	-3.186305000	0.613531000
C	-2.538149000	-2.682271000	1.471251000
Fe	-0.171875000	-0.006986000	-0.687909000
O	-0.341318000	0.085146000	-2.787037000
H	0.907160000	-2.482223000	-2.373056000
H	2.816194000	-4.044888000	-2.049258000
H	4.276277000	-3.754365000	-0.032653000
H	3.769218000	-1.921386000	1.564212000
H	3.723515000	1.941800000	1.610040000
H	4.187693000	3.818759000	0.051721000
H	2.722113000	4.117348000	-1.959496000
H	0.847799000	2.516736000	-2.317174000
H	-0.851780000	1.000460000	2.876799000
H	0.283433000	1.970732000	1.960807000
H	0.323397000	-2.048579000	1.872998000
H	-0.792204000	-1.128744000	2.863104000

H	-2.559455000	2.887831000	2.540717000
H	-4.237418000	3.865753000	0.975948000
H	-4.134577000	3.230340000	-1.448967000
H	-2.382396000	1.627395000	-2.187037000
H	-2.514651000	-1.541153000	-2.161955000
H	-4.243210000	-3.142595000	-1.422095000
H	-4.262619000	-3.875238000	0.979052000
H	-2.518286000	-2.970177000	2.515611000
H	-0.938414000	-0.435861000	-3.336791000
H	0.478095000	0.175783000	-3.289198000
C	2.258480000	-0.022131000	2.692591000
H	1.934455000	0.852293000	3.259273000
H	1.954685000	-0.916789000	3.238549000
H	3.348570000	-0.009279000	2.671926000

Table S 58: Optimized Cartesian xyz coordinates of $^{3}\mathbf{A_1}$ calculated using UB3LYP/BS1 in Gaussian.

C	-1.465174000	-2.421570000	1.541673000
C	-2.462202000	-3.365072000	1.330464000
C	-3.246262000	-3.257378000	0.187786000
C	-3.006188000	-2.214009000	-0.706438000
C	-1.995562000	-1.301091000	-0.430400000
N	-1.242817000	-1.406308000	0.691765000
C	-1.658245000	-0.079301000	-1.314673000
C	-2.113311000	1.137810000	-0.479274000
C	-3.197393000	1.948663000	-0.793798000
C	-3.537219000	2.991839000	0.068073000
C	-2.779738000	3.200449000	1.214942000
C	-1.704235000	2.356188000	1.460959000
N	-1.385110000	1.344293000	0.640206000
N	-0.145863000	-0.012616000	-1.382638000
C	0.433435000	1.251125000	-1.889507000
C	0.527050000	-1.221435000	-1.904769000
C	1.667367000	-1.708770000	-1.019518000
C	1.514080000	1.824832000	-0.980931000
C	2.431590000	2.747387000	-1.476317000
C	3.359887000	3.323373000	-0.615883000
C	3.349798000	2.953879000	0.726479000
C	2.418389000	2.018121000	1.148315000
N	1.515544000	1.458945000	0.320800000
N	1.645571000	-1.364491000	0.283702000
C	2.593450000	-1.856026000	1.103204000
C	3.598862000	-2.703166000	0.663315000
C	3.635260000	-3.047843000	-0.685555000
C	2.657514000	-2.540972000	-1.534966000
Fe	0.186993000	0.009382000	0.780012000
O	0.284010000	0.040302000	3.100300000
H	-0.817995000	-2.473221000	2.408852000
H	-2.611778000	-4.163165000	2.046146000
H	-4.030872000	-3.976883000	-0.013551000
H	-3.600472000	-2.126253000	-1.605158000
H	-3.772433000	1.782706000	-1.693896000

H	-4.379947000	3.633239000	-0.160783000
H	-3.009418000	4.000857000	1.906415000
H	-1.075786000	2.485033000	2.333932000
H	0.838987000	1.120805000	-2.897228000
H	-0.357946000	1.999876000	-1.974919000
H	-0.201629000	-2.032834000	-1.974858000
H	0.898982000	-1.056688000	-2.920399000
H	2.414300000	3.012710000	-2.526835000
H	4.078725000	4.044363000	-0.986232000
H	4.052678000	3.374008000	1.434181000
H	2.386006000	1.698313000	2.182027000
H	2.534395000	-1.556804000	2.142515000
H	4.337340000	-3.074921000	1.361813000
H	4.411840000	-3.698647000	-1.069174000
H	2.657547000	-2.791600000	-2.589278000
H	0.985152000	0.203592000	3.743451000
C	-2.300985000	-0.134339000	-2.702544000
H	-1.960558000	-1.014004000	-3.251385000
H	-3.389281000	-0.178864000	-2.654821000
H	-2.033613000	0.749914000	-3.283972000
H	-0.520404000	-0.082807000	3.619760000

Table S 59: Optimized Cartesian xyz coordinates of $^{5}\mathbf{A_1}$ calculated using UB3LYP/BS1 in Gaussian.

C	-1.818656000	-2.475509000	1.459880000
C	-2.875256000	-3.319451000	1.141221000
C	-3.562531000	-3.091565000	-0.045048000
C	-3.171787000	-2.035691000	-0.868788000
C	-2.105674000	-1.230144000	-0.480508000
N	-1.446791000	-1.452710000	0.676631000
C	-1.624016000	-0.000030000	-1.288343000
C	-2.105701000	1.230084000	-0.480518000
C	-3.171816000	2.035615000	-0.868828000
C	-3.562592000	3.091492000	-0.045108000
C	-2.875344000	3.319399000	1.141172000
C	-1.818740000	2.475471000	1.459861000
N	-1.446846000	1.452666000	0.676634000
N	-0.104661000	-0.000017000	-1.236223000
C	0.520168000	1.234029000	-1.780440000
C	0.520194000	-1.234054000	-1.780430000
C	1.676229000	-1.765713000	-0.946807000
C	1.676157000	1.765751000	-0.946794000
C	2.668020000	2.544266000	-1.538174000
C	3.653235000	3.115554000	-0.738703000
C	3.622009000	2.890326000	0.635225000
C	2.611977000	2.090709000	1.148468000
N	1.657292000	1.534632000	0.380908000
N	1.657365000	-1.534617000	0.380902000
C	2.612100000	-2.090647000	1.148432000
C	3.622181000	-2.890184000	0.635161000
C	3.653402000	-3.115385000	-0.738771000
C	2.668135000	-2.544150000	-1.538216000

Fe	0.202831000	-0.000011000	0.944187000
O	0.299492000	-0.000021000	3.149176000
H	-1.244406000	-2.622164000	2.367927000
H	-3.144395000	-4.130825000	1.805158000
H	-4.390765000	-3.727655000	-0.334156000
H	-3.696358000	-1.858625000	-1.796832000
H	-3.696365000	1.858534000	-1.796882000
H	-4.390830000	3.727566000	-0.334239000
H	-3.144507000	4.130776000	1.805095000
H	-1.244510000	2.622149000	2.367917000
H	0.854283000	1.081916000	-2.810767000
H	-0.236327000	2.021606000	-1.815282000
H	-0.236276000	-2.021658000	-1.815224000
H	0.854269000	-1.081957000	-2.810772000
H	2.665092000	2.706001000	-2.609608000
H	4.431725000	3.726058000	-1.180648000
H	4.365312000	3.318418000	1.295312000
H	2.556354000	1.887327000	2.211695000
H	2.556484000	-1.887292000	2.211664000
H	4.365522000	-3.318236000	1.295230000
H	4.431928000	-3.725829000	-1.180738000
H	2.665202000	-2.705865000	-2.609652000
H	1.055567000	-0.000241000	3.749026000
H	-0.489996000	0.000156000	3.705018000
C	-2.139180000	-0.000044000	-2.732554000
H	-1.789425000	0.883636000	-3.268555000
H	-1.789405000	-0.883723000	-3.268543000
H	-3.227314000	-0.000057000	-2.784724000

Table S 60: Optimized Cartesian xyz coordinates of $^{1}\mathbf{B_1}$ calculated using UB3LYP/BS1 in Gaussian.

C	1.571990000	2.343508000	1.223308000
C	2.608366000	3.238238000	0.978568000
C	3.351945000	3.102658000	-0.187990000
C	3.041155000	2.074561000	-1.079136000
C	1.994367000	1.216793000	-0.768255000
N	1.274109000	1.356065000	0.367980000
C	1.560198000	-0.000003000	-1.605085000
C	1.994378000	-1.216790000	-0.768248000
C	3.041178000	-2.074547000	-1.079122000
C	3.351979000	-3.102634000	-0.187970000
C	2.608398000	-3.238219000	0.978586000
C	1.572010000	-2.343500000	1.223320000
N	1.274120000	-1.356065000	0.367986000
N	0.029686000	-0.000010000	-1.545095000
C	-0.608168000	-1.240597000	-2.080692000
C	-0.608183000	1.240566000	-2.080702000
C	-1.677195000	1.773287000	-1.144445000
C	-1.677196000	-1.773302000	-1.144443000
C	-2.665526000	-2.655799000	-1.565719000
C	-3.552275000	-3.171586000	-0.622882000
C	-3.422932000	-2.787127000	0.708603000

C	-2.420338000	-1.889069000	1.054381000
N	-1.568389000	-1.393100000	0.142778000
N	-1.568389000	1.393086000	0.142776000
C	-2.420329000	1.889067000	1.054382000
C	-3.422911000	2.787138000	0.708607000
C	-3.552251000	3.171600000	-0.622878000
C	-2.665513000	2.655800000	-1.565717000
C	2.104516000	-0.000006000	-3.032573000
Fe	-0.175892000	-0.000007000	0.465565000
O	-0.596856000	-0.000007000	2.355374000
Cl	0.664571000	0.000009000	3.543758000
H	0.968681000	2.389396000	2.120184000
H	2.824140000	4.019090000	1.696371000
H	4.164415000	3.784980000	-0.407388000
H	3.607435000	1.954111000	-1.992647000
H	3.607459000	-1.954094000	-1.992633000
H	4.164459000	-3.784947000	-0.407363000
H	2.824181000	-4.019065000	1.696393000
H	0.968698000	-2.389393000	2.120193000
H	-1.018493000	-1.072110000	-3.080126000
H	0.160134000	-2.010403000	-2.182009000
H	0.160113000	2.010373000	-2.182054000
H	-1.018530000	1.072056000	-3.080124000
H	-2.737821000	-2.937562000	-2.609616000
H	-4.330972000	-3.861473000	-0.925733000
H	-4.090119000	-3.168268000	1.471126000
H	-2.256474000	-1.532810000	2.065611000
H	-2.256467000	1.532805000	2.065611000
H	-4.090091000	3.168288000	1.471132000
H	-4.330939000	3.861499000	-0.925726000
H	-2.737807000	2.937565000	-2.609614000
H	3.194999000	0.000000000	-3.047349000
H	1.769709000	-0.884780000	-3.577079000
H	1.769700000	0.884761000	-3.577087000

Table S 61: Optimized Cartesian xyz coordinates of $^{3}\mathbf{B_1}$ calculated using UB3LYP/BS1 in Gaussian.

C	-1.501887000	-2.447482000	1.295625000
C	-2.451790000	-3.422235000	1.013803000
C	-3.146639000	-3.352304000	-0.186792000
C	-2.867137000	-2.310187000	-1.070467000
C	-1.905530000	-1.368295000	-0.725853000
N	-1.235188000	-1.438517000	0.452057000
C	-1.545864000	-0.146053000	-1.597855000
C	-2.187919000	1.058364000	-0.876275000
C	-3.314332000	1.743396000	-1.323805000
C	-3.836062000	2.760851000	-0.523829000
C	-3.224170000	3.059746000	0.688304000
C	-2.095683000	2.332856000	1.057066000
N	-1.595894000	1.362472000	0.287396000
N	-0.031841000	0.000640000	-1.477075000
C	0.521359000	1.300453000	-1.953214000

C	0.703458000	-1.158756000	-2.056871000
C	1.934788000	-1.547583000	-1.259099000
C	1.539005000	1.887528000	-0.990078000
C	2.457558000	2.848470000	-1.399251000
C	3.304110000	3.422161000	-0.453950000
C	3.207280000	3.014350000	0.873211000
C	2.277743000	2.038858000	1.209155000
N	1.462768000	1.485374000	0.295146000
N	1.845812000	-1.369921000	0.064187000
C	2.850561000	-1.775906000	0.849946000
C	3.991535000	-2.386746000	0.339158000
C	4.092851000	-2.562039000	-1.038094000
C	3.048734000	-2.132650000	-1.854899000
C	-2.000702000	-0.273070000	-3.054499000
Fe	0.155891000	0.004360000	0.645976000
O	0.513995000	0.176582000	2.472686000
Cl	-0.544386000	-0.331344000	3.755807000
H	-0.943128000	-2.448410000	2.221825000
H	-2.636119000	-4.213592000	1.728839000
H	-3.893212000	-4.095552000	-0.439829000
H	-3.394136000	-2.242790000	-2.012128000
H	-3.785180000	1.502566000	-2.266759000
H	-4.712642000	3.309094000	-0.848336000
H	-3.607781000	3.836408000	1.337554000
H	-1.574840000	2.516570000	1.990258000
H	0.963315000	1.195772000	-2.948650000
H	-0.297713000	2.016562000	-2.048268000
H	0.033245000	-2.021309000	-2.052100000
H	0.969961000	-0.968897000	-3.101055000
H	2.505345000	3.146079000	-2.440161000
H	4.026004000	4.173616000	-0.750909000
H	3.843794000	3.436800000	1.640104000
H	2.144456000	1.666744000	2.218892000
H	2.721959000	-1.592853000	1.911266000
H	4.781358000	-2.707106000	1.006745000
H	4.972165000	-3.023150000	-1.472214000
H	3.097943000	-2.255101000	-2.930759000
H	-3.083584000	-0.366606000	-3.134581000
H	-1.706254000	0.610175000	-3.625045000
H	-1.562735000	-1.150884000	-3.530897000

Table S 62: Optimized Cartesian xyz coordinates of $^{5}\mathbf{B_1}$ calculated using UB3LYP/BS1 in Gaussian.

C	-1.876467000	-2.417792000	1.153505000
C	-2.924353000	-3.262105000	0.802693000
C	-3.547347000	-3.066134000	-0.423946000
C	-3.112095000	-2.032834000	-1.253435000
C	-2.060639000	-1.224716000	-0.829123000
N	-1.456109000	-1.428546000	0.355834000
C	-1.539673000	0.000011000	-1.619268000
C	-2.060631000	1.224734000	-0.829112000
C	-3.112080000	2.032865000	-1.253418000

C	-3.547324000	3.066160000	-0.423919000
C	-2.924331000	3.262114000	0.802723000
C	-1.876453000	2.417788000	1.153528000
N	-1.456102000	1.428548000	0.355848000
N	-0.032245000	0.000006000	-1.504809000
C	0.612909000	1.230094000	-2.020093000
C	0.612900000	-1.230084000	-2.020102000
C	1.751228000	-1.739859000	-1.149597000
C	1.751234000	1.739862000	-1.149582000
C	2.780978000	2.508517000	-1.687949000
C	3.741133000	3.042303000	-0.832816000
C	3.647091000	2.787022000	0.532712000
C	2.600603000	1.995562000	0.990032000
N	1.673136000	1.485937000	0.164329000
N	1.673130000	-1.485948000	0.164316000
C	2.600599000	-1.995578000	0.990013000
C	3.647089000	-2.787031000	0.532684000
C	3.741130000	-3.042298000	-0.832847000
C	2.780972000	-2.508507000	-1.687974000
C	-1.999624000	0.000019000	-3.082870000
Fe	0.175565000	-0.000005000	0.822813000
O	0.669226000	0.000005000	2.684054000
Cl	-0.561415000	-0.000041000	3.881058000
H	-1.356471000	-2.522260000	2.099151000
H	-3.236935000	-4.048804000	1.477249000
H	-4.363711000	-3.706094000	-0.737330000
H	-3.591230000	-1.871969000	-2.208894000
H	-3.591215000	1.872014000	-2.208880000
H	-4.363683000	3.706130000	-0.737298000
H	-3.236908000	4.048808000	1.477286000
H	-1.356457000	2.522243000	2.099176000
H	0.971099000	1.087861000	-3.045657000
H	-0.136454000	2.024172000	-2.061516000
H	-0.136467000	-2.024158000	-2.061523000
H	0.971086000	-1.087848000	-3.045667000
H	2.827214000	2.689297000	-2.755671000
H	4.550513000	3.645173000	-1.227485000
H	4.372434000	3.183393000	1.231739000
H	2.479610000	1.744243000	2.038439000
H	2.479608000	-1.744269000	2.038422000
H	4.372434000	-3.183407000	1.231705000
H	4.550510000	-3.645165000	-1.227523000
H	2.827208000	-2.689276000	-2.755697000
H	-3.085355000	0.000024000	-3.176225000
H	-1.627519000	0.883968000	-3.603496000
H	-1.627527000	-0.883930000	-3.603503000

Table S 63: Optimized Cartesian xyz coordinates of $^{1}\mathbf{E_1}$ calculated using UB3LYP/BS1 in Gaussian.

C	-2.559857000	3.205212000	-1.399443000
C	-3.362593000	3.085019000	-0.270935000
C	-3.099830000	2.075507000	0.659330000

C	-2.035182000	1.218035000	0.428244000
N	-1.255161000	1.357493000	-0.667240000
C	-1.649690000	0.000000000	1.287871000
C	-2.035182000	-1.218035000	0.428244000
C	-3.099829000	-2.075510000	0.659330000
C	-3.362590000	-3.085021000	-0.270935000
C	-2.559854000	-3.205214000	-1.399443000
C	-1.503056000	-2.317071000	-1.567987000
N	-1.255161000	-1.357493000	-0.667240000
N	-0.113298000	0.000000000	1.303745000
C	0.489999000	-1.244974000	1.876317000
C	0.489999000	1.244974000	1.876317000
C	1.573163000	1.799941000	0.976587000
C	1.573163000	-1.799941000	0.976587000
C	2.518369000	-2.714714000	1.425053000
C	3.426215000	-3.250712000	0.515232000
C	3.362907000	-2.857186000	-0.819252000
C	2.404974000	-1.929527000	-1.199707000
N	1.531714000	-1.412815000	-0.314641000
N	1.531713000	1.412815000	-0.314641000
C	2.404974000	1.929530000	-1.199707000
C	3.362905000	2.857188000	-0.819252000
C	3.426213000	3.250714000	0.515232000
C	2.518367000	2.714716000	1.425053000
C	-1.503058000	2.317071000	-1.567987000
C	-2.263418000	-0.000001000	2.684470000
H	-2.743401000	3.969083000	-2.143992000
H	-4.190540000	3.765460000	-0.110931000
H	-3.720472000	1.968883000	1.538624000
H	-3.720471000	-1.968885000	1.538624000
H	-4.190537000	-3.765462000	-0.110931000
H	-2.743398000	-3.969086000	-2.143992000
H	-0.844224000	-2.346455000	-2.426785000
H	0.876442000	-1.062407000	2.881027000
H	-0.290263000	-2.003044000	1.974011000
H	-0.290264000	2.003044000	1.974011000
H	0.876442000	1.062407000	2.881027000
H	2.540139000	-3.006257000	2.468293000
H	4.170732000	-3.965825000	0.844242000
H	4.046216000	-3.255492000	-1.558175000
H	2.303640000	-1.582705000	-2.220115000
H	2.303639000	1.582707000	-2.220115000
H	4.046214000	3.255494000	-1.558175000
H	4.170730000	3.965828000	0.844242000
H	2.540136000	3.006260000	2.468293000
H	-0.844226000	2.346455000	-2.426785000
H	-1.961623000	0.884776000	3.247270000
H	-3.353002000	-0.000001000	2.637465000
H	-1.961623000	-0.884776000	3.247270000
Fe	0.199457000	0.000000000	-0.748389000
O	0.451566000	0.000000000	-2.357687000

Table S 64: Optimized Cartesian xyz coordinates of $^{3}\mathbf{E_1}$ calculated using UB3LYP/BS1 in Gaussian.

C	2.597162000	-3.187728000	-1.396542000
C	3.383475000	-3.075740000	-0.255762000
C	3.106328000	-2.072801000	0.677131000
C	2.043901000	-1.215019000	0.436997000
N	1.277419000	-1.347062000	-0.669183000
C	1.651123000	-0.000001000	1.297328000
C	2.043901000	1.215021000	0.437002000
C	3.106327000	2.072802000	0.677135000
C	3.383479000	3.075737000	-0.255761000
C	2.597167000	3.187723000	-1.396541000
C	1.541529000	2.299894000	-1.572813000
N	1.277417000	1.347063000	-0.669179000
N	0.117789000	-0.000001000	1.301697000
C	-0.495152000	1.244316000	1.867568000
C	-0.495150000	-1.244320000	1.867566000
C	-1.589836000	-1.780934000	0.970163000
C	-1.589833000	1.780936000	0.970162000
C	-2.546330000	2.683503000	1.419163000
C	-3.464492000	3.204022000	0.510535000
C	-3.401769000	2.805473000	-0.822388000
C	-2.432128000	1.889749000	-1.203118000
N	-1.547716000	1.390234000	-0.320437000
N	-1.547720000	-1.390233000	-0.320437000
C	-2.432136000	-1.889743000	-1.203116000
C	-3.401780000	-2.805464000	-0.822384000
C	-3.464502000	-3.204013000	0.510538000
C	-2.546337000	-2.683496000	1.419166000
C	1.541527000	-2.299894000	-1.572816000
C	2.256085000	-0.000007000	2.697675000
H	2.793216000	-3.944190000	-2.145410000
H	4.210072000	-3.756227000	-0.089137000
H	3.714470000	-1.970954000	1.565661000
H	3.714467000	1.970958000	1.565667000
H	4.210076000	3.756222000	-0.089136000
H	2.793222000	3.944182000	-2.145411000
H	0.898722000	2.325231000	-2.443417000
H	-0.873297000	1.065550000	2.876134000
H	0.279383000	2.009611000	1.953816000
H	0.279385000	-2.009615000	1.953803000
H	-0.873287000	-1.065559000	2.876136000
H	-2.568576000	2.978429000	2.461442000
H	-4.217309000	3.910320000	0.839690000
H	-4.094832000	3.189808000	-1.559559000
H	-2.335390000	1.534214000	-2.220927000
H	-2.335395000	-1.534208000	-2.220925000
H	-4.094845000	-3.189799000	-1.559554000
H	-4.217321000	-3.910308000	0.839694000
H	-2.568584000	-2.978421000	2.461445000
H	0.898722000	-2.325228000	-2.443422000

H	1.949950000	-0.884707000	3.258409000
H	3.345910000	-0.000009000	2.659062000
H	1.949955000	0.884696000	3.258412000
Fe	-0.190006000	-0.000002000	-0.752548000
O	-0.414279000	-0.000006000	-2.360598000

Table S 65: Optimized Cartesian xyz coordinates of $^{5}\mathbf{E_1}$ calculated using UB3LYP/BS1 in Gaussian.

C	2.800755000	-3.216334000	-1.298065000
C	3.517075000	-3.054552000	-0.117920000
C	3.157874000	-2.040965000	0.775135000
C	2.084149000	-1.221750000	0.456815000
N	1.386990000	-1.398635000	-0.685767000
C	1.625718000	-0.000001000	1.278494000
C	2.084150000	1.221749000	0.456815000
C	3.157874000	2.040962000	0.775136000
C	3.517076000	3.054549000	-0.117919000
C	2.800757000	3.216334000	-1.298063000
C	1.732070000	2.362554000	-1.549390000
N	1.386990000	1.398635000	-0.685766000
N	0.088280000	0.000000000	1.205050000
C	-0.525083000	1.244241000	1.784759000
C	-0.525084000	-1.244241000	1.784759000
C	-1.655479000	-1.785932000	0.936772000
C	-1.655477000	1.785932000	0.936771000
C	-2.627824000	2.634946000	1.451308000
C	-3.570271000	3.183045000	0.582946000
C	-3.517818000	2.865323000	-0.772608000
C	-2.531523000	1.996250000	-1.215067000
N	-1.622370000	1.471682000	-0.373516000
N	-1.622371000	-1.471682000	-0.373515000
C	-2.531525000	-1.996249000	-1.215066000
C	-3.517821000	-2.865321000	-0.772607000
C	-3.570275000	-3.183042000	0.582947000
C	-2.627826000	-2.634945000	1.451309000
C	1.732069000	-2.362554000	-1.549391000
C	2.138008000	-0.000001000	2.718162000
H	3.059973000	-3.983928000	-2.015809000
H	4.352901000	-3.705114000	0.111052000
H	3.714578000	-1.904495000	1.692065000
H	3.714578000	1.904491000	1.692066000
H	4.352904000	3.705110000	0.111055000
H	3.059976000	3.983926000	-2.015807000
H	1.138861000	2.434180000	-2.453044000
H	-0.862052000	1.063370000	2.807334000
H	0.247454000	2.014302000	1.838338000
H	0.247453000	-2.014304000	1.838337000
H	-0.862052000	-1.063371000	2.807334000
H	-2.645884000	2.870842000	2.508587000
H	-4.335644000	3.850123000	0.961675000
H	-4.230992000	3.275556000	-1.475873000
H	-2.444542000	1.699064000	-2.253078000

H	-2.444544000	-1.699063000	-2.253078000
H	-4.230997000	-3.275551000	-1.475872000
H	-4.335649000	-3.850117000	0.961677000
H	-2.645886000	-2.870841000	2.508588000
H	1.138860000	-2.434179000	-2.453045000
H	1.799722000	-0.885164000	3.258692000
H	3.227552000	-0.000002000	2.749418000
H	1.799722000	0.885162000	3.258692000
Fe	-0.213542000	0.000000000	-0.880253000
O	-0.442030000	0.000000000	-2.478038000

Table S 66: Optimized Cartesian xyz coordinates of $^{2}\mathbf{C_1}$ calculated using UB3LYP/BS1 in Gaussian.

C	-1.533555000	2.342328000	-1.545470000
C	-2.581155000	3.238136000	-1.363882000
C	-3.388356000	3.104844000	-0.239773000
C	-3.130695000	2.080148000	0.673996000
C	-2.074954000	1.215719000	0.425784000
N	-1.297888000	1.352503000	-0.672738000
C	-1.685930000	0.000002000	1.283772000
C	-2.074988000	-1.215696000	0.425771000
C	-3.130759000	-2.080092000	0.673970000
C	-3.388448000	-3.104772000	-0.239808000
C	-2.581245000	-3.238083000	-1.363913000
C	-1.533614000	-2.342308000	-1.545488000
N	-1.297920000	-1.352496000	-0.672749000
N	-0.146815000	-0.000020000	1.285227000
C	0.466288000	-1.244723000	1.862782000
C	0.466326000	1.244656000	1.862800000
C	1.588433000	1.762938000	0.988397000
C	1.588429000	-1.762970000	0.988405000
C	2.554273000	-2.647232000	1.451895000
C	3.493943000	-3.148620000	0.553344000
C	3.441937000	-2.748804000	-0.779431000
C	2.459976000	-1.849992000	-1.172975000
N	1.556052000	-1.371189000	-0.301353000
N	1.556058000	1.371150000	-0.301356000
C	2.459964000	1.849973000	-1.172988000
C	3.441901000	2.748815000	-0.779456000
C	3.493900000	3.148645000	0.553316000
C	2.554252000	2.647236000	1.451876000
Fe	0.172296000	-0.000017000	-0.715471000
O	0.528488000	-0.000020000	-2.485482000
H	-0.865742000	2.402679000	-2.395626000
H	-2.752319000	4.020465000	-2.092026000
H	-4.209571000	3.790502000	-0.067972000
H	-3.745553000	1.969506000	1.556840000
H	-3.745618000	-1.969437000	1.556811000
H	-4.209687000	-3.790404000	-0.068018000
H	-2.752430000	-4.020402000	-2.092063000
H	-0.865797000	-2.402676000	-2.395640000
H	0.813985000	-1.064444000	2.881656000

H	-0.304447000	-2.016024000	1.923461000
H	-0.304400000	2.015961000	1.923560000
H	0.814077000	1.064327000	2.881647000
H	2.568914000	-2.943581000	2.493889000
H	4.255634000	-3.841042000	0.891428000
H	4.153774000	-3.118232000	-1.506270000
H	2.364948000	-1.487650000	-2.188931000
H	2.364939000	1.487620000	-2.188940000
H	4.153724000	3.118257000	-1.506301000
H	4.255572000	3.841094000	0.891390000
H	2.568890000	2.943592000	2.493867000
H	-0.232768000	-0.000013000	-3.078412000
C	-2.286691000	-0.000001000	2.685664000
H	-1.979427000	0.884785000	3.245612000
H	-3.376634000	0.000017000	2.649800000
H	-1.979454000	-0.884806000	3.245596000

Table S 67: Optimized Cartesian xyz coordinates of $^{4}\mathbf{C_1}$ calculated using UB3LYP/BS1 in Gaussian.

C	-1.534598000	2.364820000	-1.536875000
C	-2.580822000	3.258376000	-1.342407000
C	-3.377280000	3.114896000	-0.212252000
C	-3.110714000	2.083258000	0.690326000
C	-2.054217000	1.220072000	0.433755000
N	-1.287282000	1.374521000	-0.667628000
C	-1.672761000	0.000021000	1.298705000
C	-2.054255000	-1.220024000	0.433762000
C	-3.110778000	-2.083177000	0.690341000
C	-3.377375000	-3.114813000	-0.212229000
C	-2.580922000	-3.258325000	-1.342384000
C	-1.534672000	-2.364801000	-1.536858000
N	-1.287326000	-1.374503000	-0.667619000
N	-0.160863000	-0.000001000	1.369635000
C	0.454268000	-1.237895000	1.904477000
C	0.454300000	1.237874000	1.904484000
C	1.561820000	1.773684000	1.012201000
C	1.561783000	-1.773718000	1.012195000
C	2.510246000	-2.671393000	1.491755000
C	3.454670000	-3.197831000	0.615455000
C	3.430635000	-2.809233000	-0.722000000
C	2.469652000	-1.899597000	-1.135794000
N	1.557016000	-1.398228000	-0.281158000
N	1.557051000	1.398191000	-0.281149000
C	2.469696000	1.899545000	-1.135785000
C	3.430690000	2.809169000	-0.721991000
C	3.454726000	3.197772000	0.615463000
C	2.510293000	2.671349000	1.491762000
Fe	0.204913000	-0.000006000	-0.866914000
O	0.536790000	-0.000016000	-2.674421000
H	-0.878477000	2.423171000	-2.395997000
H	-2.759682000	4.044028000	-2.065058000
H	-4.199008000	3.796806000	-0.028075000

H	-3.721091000	1.965399000	1.574877000
H	-3.721152000	-1.965295000	1.574892000
H	-4.199123000	-3.796698000	-0.028047000
H	-2.759805000	-4.043977000	-2.065029000
H	-0.878554000	-2.423176000	-2.395982000
H	0.839170000	-1.079316000	2.915535000
H	-0.311246000	-2.013289000	1.986856000
H	-0.311196000	2.013284000	1.986880000
H	0.839207000	1.079274000	2.915537000
H	2.504704000	-2.956191000	2.537261000
H	4.199367000	-3.899322000	0.972346000
H	4.147053000	-3.195073000	-1.435663000
H	2.399024000	-1.546604000	-2.156981000
H	2.399066000	1.546551000	-2.156971000
H	4.147116000	3.194997000	-1.435653000
H	4.199431000	3.899255000	0.972353000
H	2.504752000	2.956149000	2.537267000
H	0.120278000	-0.000021000	-3.539575000
C	-2.331024000	0.000034000	2.678862000
H	-2.036384000	0.883335000	3.248069000
H	-3.419444000	0.000050000	2.613839000
H	-2.036410000	-0.883273000	3.248073000

Table S 68: Optimized Cartesian xyz coordinates of $^{6}\mathbf{C_1}$ calculated using UB3LYP/BS1 in Gaussian.

C	1.741371000	-2.411782000	-1.508661000
C	2.800459000	-3.269847000	-1.238125000
C	3.519251000	-3.082955000	-0.063221000
C	3.164055000	-2.046406000	0.802683000
C	2.096037000	-1.224776000	0.465625000
N	1.402056000	-1.417692000	-0.674973000
C	1.639220000	0.000000000	1.286947000
C	2.096035000	1.224777000	0.465626000
C	3.164051000	2.046410000	0.802684000
C	3.519245000	3.082960000	-0.063219000
C	2.800452000	3.269851000	-1.238123000
C	1.741366000	2.411784000	-1.508659000
N	1.402053000	1.417692000	-0.674972000
N	0.117701000	-0.000001000	1.242228000
C	-0.494136000	1.236169000	1.807703000
C	-0.494134000	-1.236172000	1.807703000
C	-1.638514000	-1.774523000	0.970997000
C	-1.638514000	1.774521000	0.970996000
C	-2.606435000	2.610445000	1.517794000
C	-3.574482000	3.162077000	0.682352000
C	-3.552851000	2.859608000	-0.677618000
C	-2.569566000	2.004888000	-1.151424000
N	-1.631901000	1.476439000	-0.341217000
N	-1.631901000	-1.476441000	-0.341216000
C	-2.569567000	-2.004890000	-1.151422000
C	-3.552853000	-2.859608000	-0.677616000
C	-3.574484000	-3.162076000	0.682355000

C	-2.606436000	-2.610445000	1.517796000
Fe	-0.221603000	-0.000001000	-0.997379000
O	-0.499123000	-0.000002000	-2.770651000
H	1.145743000	-2.510792000	-2.408158000
H	3.049442000	-4.060214000	-1.934408000
H	4.349699000	-3.733920000	0.183476000
H	3.717737000	-1.895249000	1.718707000
H	3.717733000	1.895252000	1.718708000
H	4.349691000	3.733926000	0.183479000
H	3.049433000	4.060219000	-1.934404000
H	1.145738000	2.510793000	-2.408156000
H	-0.828914000	1.070278000	2.834852000
H	0.269819000	2.015723000	1.853374000
H	0.269821000	-2.015726000	1.853371000
H	-0.828910000	-1.070282000	2.834853000
H	-2.600119000	2.831501000	2.578505000
H	-4.335629000	3.817922000	1.088232000
H	-4.287148000	3.269815000	-1.358766000
H	-2.509418000	1.720301000	-2.194765000
H	-2.509419000	-1.720303000	-2.194764000
H	-4.287151000	-3.269815000	-1.358763000
H	-4.335632000	-3.817919000	1.088235000
H	-2.600120000	-2.831500000	2.578507000
H	-0.273997000	-0.000002000	-3.703396000
C	2.178237000	0.000000000	2.719876000
H	1.842787000	-0.884112000	3.264071000
H	3.267780000	0.000001000	2.742949000
H	1.842785000	0.884111000	3.264071000

Table S 69: Optimized Cartesian xyz coordinates of $^{2}\mathbf{D_1}$ calculated using UB3LYP/BS1 in Gaussian.

C	-1.553868000	-2.365847000	1.214750000
C	-2.584660000	-3.265843000	0.968727000
C	-3.340341000	-3.119920000	-0.188237000
C	-3.044311000	-2.081687000	-1.074767000
C	-2.005604000	-1.217345000	-0.765315000
N	-1.282787000	-1.361453000	0.369091000
C	-1.574430000	0.000002000	-1.596957000
C	-2.005588000	1.217358000	-0.765321000
C	-3.044283000	2.081714000	-1.074777000
C	-3.340303000	3.119950000	-0.188248000
C	-2.584626000	3.265863000	0.968721000
C	-1.553846000	2.365853000	1.214747000
N	-1.282773000	1.361457000	0.369088000
N	-0.036749000	-0.000008000	-1.514070000
C	0.603385000	1.244102000	-2.061879000
C	0.603366000	-1.244131000	-2.061871000
C	1.679874000	-1.763715000	-1.134768000
C	1.679881000	1.763696000	-1.134768000
C	2.665945000	2.647420000	-1.555096000
C	3.561936000	3.153740000	-0.616167000
C	3.447504000	2.758022000	0.713854000

C	2.449324000	1.859351000	1.062883000
N	1.585556000	1.375242000	0.153267000
N	1.585552000	-1.375257000	0.153267000
C	2.449330000	-1.859353000	1.062879000
C	3.447517000	-2.758016000	0.713848000
C	3.561945000	-3.153738000	-0.616172000
C	2.665944000	-2.647429000	-1.555098000
C	-2.094806000	0.000001000	-3.030779000
Fe	0.175717000	-0.000005000	0.500728000
O	0.514783000	-0.000004000	2.270887000
Cl	-0.718333000	-0.000002000	3.465209000
H	-0.938685000	-2.433161000	2.101671000
H	-2.783778000	-4.059568000	1.677089000
H	-4.149444000	-3.806375000	-0.407710000
H	-3.616097000	-1.961492000	-1.984773000
H	-3.616066000	1.961527000	-1.984785000
H	-4.149396000	3.806416000	-0.407723000
H	-2.783739000	4.059588000	1.677083000
H	-0.938666000	2.433159000	2.101671000
H	1.001440000	1.062153000	-3.061771000
H	-0.163735000	2.014845000	-2.161532000
H	-0.163761000	-2.014870000	-2.161495000
H	1.001404000	-1.062203000	-3.061773000
H	2.728701000	2.939597000	-2.596479000
H	4.337724000	3.846154000	-0.920532000
H	4.123354000	3.130200000	1.472923000
H	2.314299000	1.503003000	2.076293000
H	2.314307000	-1.503004000	2.076289000
H	4.123375000	-3.130185000	1.472915000
H	4.337738000	-3.846146000	-0.920538000
H	2.728697000	-2.939609000	-2.596480000
H	-3.184977000	0.000009000	-3.055132000
H	-1.757621000	0.885018000	-3.572717000
H	-1.757634000	-0.885024000	-3.572710000

Table S 70: Optimized Cartesian xyz coordinates of $^{4}\mathbf{D_1}$ calculated using UB3LYP/BS1 in Gaussian.

C	-1.573552000	-2.355203000	1.202066000
C	-2.610641000	-3.247140000	0.955304000
C	-3.343155000	-3.109358000	-0.217344000
C	-3.026579000	-2.082619000	-1.110307000
C	-1.985076000	-1.219734000	-0.801627000
N	-1.276877000	-1.371758000	0.340172000
C	-1.559635000	-0.000052000	-1.647000000
C	-1.985141000	1.219631000	-0.801661000
C	-3.026686000	2.082455000	-1.110369000
C	-3.343316000	3.109202000	-0.217434000
C	-2.610813000	3.247051000	0.955213000
C	-1.573681000	2.355170000	1.202002000
N	-1.276955000	1.371719000	0.340134000
N	-0.044214000	-0.000015000	-1.640469000
C	0.600134000	1.239293000	-2.138844000

C	0.600189000	-1.239294000	-2.138840000
C	1.655804000	-1.775938000	-1.185194000
C	1.655714000	1.775996000	-1.185191000
C	2.626370000	2.677344000	-1.609899000
C	3.519156000	3.205602000	-0.681965000
C	3.422714000	2.815438000	0.651817000
C	2.443869000	1.901853000	1.010716000
N	1.581553000	1.397827000	0.106140000
N	1.581626000	-1.397773000	0.106135000
C	2.443969000	-1.901754000	1.010709000
C	3.422862000	-2.815288000	0.651808000
C	3.519322000	-3.205446000	-0.681973000
C	2.626506000	-2.677236000	-1.609906000
C	-2.145080000	-0.000085000	-3.059212000
Fe	0.196812000	0.000005000	0.589885000
O	0.445394000	0.000029000	2.443779000
Cl	-0.570191000	0.000029000	3.798238000
H	-0.972422000	-2.409409000	2.100625000
H	-2.831651000	-4.026314000	1.673251000
H	-4.155116000	-3.790830000	-0.442055000
H	-3.588503000	-1.968209000	-2.026840000
H	-3.588602000	1.967995000	-2.026901000
H	-4.155310000	3.790630000	-0.442166000
H	-2.831865000	4.026232000	1.673139000
H	-0.972558000	2.409430000	2.100563000
H	1.041382000	1.080824000	-3.126478000
H	-0.160473000	2.013833000	-2.263687000
H	-0.160383000	-2.013872000	-2.263666000
H	1.041416000	-1.080813000	-3.126482000
H	2.677544000	2.964281000	-2.653573000
H	4.279880000	3.910282000	-0.996241000
H	4.096451000	3.203405000	1.404816000
H	2.320917000	1.551464000	2.028125000
H	2.321003000	-1.551370000	2.028118000
H	4.096621000	-3.203219000	1.404807000
H	4.280082000	-3.910086000	-0.996250000
H	2.677692000	-2.964170000	-2.653580000
H	-3.235375000	-0.000112000	-3.050014000
H	-1.821622000	0.883313000	-3.612415000
H	-1.821579000	-0.883482000	-3.612393000

Table S 71: Optimized Cartesian xyz coordinates of $^{6}\mathbf{D_1}$.txt calculated using UB3LYP/BS1 in Gaussian.

C	1.765756000	2.400868000	1.189384000
C	2.818354000	3.257471000	0.889190000
C	3.496753000	3.076678000	-0.310199000
C	3.111098000	2.045905000	-1.170535000
C	2.053046000	1.224817000	-0.803604000
N	1.395834000	1.414211000	0.359838000
C	1.571534000	0.000610000	-1.612622000
C	2.053169000	-1.224012000	-0.804308000
C	3.111307000	-2.044777000	-1.171715000

C	3.497054000	-3.076026000	-0.311990000
C	2.818655000	-3.257603000	0.887280000
C	1.765971000	-2.401280000	1.187971000
N	1.395962000	-1.414159000	0.359016000
N	0.050634000	0.000501000	-1.522960000
C	-0.580678000	-1.237041000	-2.065759000
C	-0.580812000	1.238284000	-2.065059000
C	-1.695542000	1.778682000	-1.188864000
C	-1.695405000	-1.777988000	-1.189900000
C	-2.676495000	-2.619235000	-1.703677000
C	-3.614067000	-3.175040000	-0.836651000
C	-3.549899000	-2.871930000	0.521882000
C	-2.556486000	-2.011802000	0.963150000
N	-1.648590000	-1.478392000	0.122061000
N	-1.648671000	1.478366000	0.122931000
C	-2.556552000	2.011277000	0.964355000
C	-3.550017000	2.871605000	0.523596000
C	-3.614252000	3.175455000	-0.834769000
C	-2.676690000	2.620168000	-1.702138000
C	2.066234000	0.001048000	-3.061027000
Fe	-0.222196000	-0.000117000	0.701377000
O	-0.428954000	-0.000501000	2.512299000
Cl	0.339611000	-0.002434000	4.002147000
H	1.204125000	2.495403000	2.111213000
H	3.093402000	4.041531000	1.582779000
H	4.320352000	3.727147000	-0.580092000
H	3.635054000	1.898672000	-2.104552000
H	3.635259000	-1.896938000	-2.105638000
H	4.320721000	-3.726253000	-0.582261000
H	3.093767000	-4.042052000	1.580402000
H	1.204353000	-2.496431000	2.109746000
H	-0.951344000	-1.070950000	-3.080382000
H	0.182380000	-2.015459000	-2.138062000
H	0.182177000	2.016805000	-2.136985000
H	-0.951513000	1.072705000	-3.079753000
H	-2.703513000	-2.841906000	-2.763723000
H	-4.384521000	-3.835196000	-1.217213000
H	-4.258982000	-3.286038000	1.226960000
H	-2.465242000	-1.729089000	2.005070000
H	-2.465237000	1.728003000	2.006117000
H	-4.259087000	3.285298000	1.228929000
H	-4.384750000	3.835785000	-1.214938000
H	-2.703757000	2.843425000	-2.762060000
H	3.154559000	0.001134000	-3.117037000
H	1.714437000	-0.882997000	-3.594930000
H	1.714323000	0.885349000	-3.594431000

References

[1] (a) M. Lubben, A. Meetsma, E. C. Wilkinson, B. L. Feringa, L. Que, Jr., *Angew. Chem., Int. Ed. Engl.* **1995**, *34*, 1512-1514; (b) A. Draksharapu, Q. Li, H. Logtenberg, T. A. van den Berg, A. Meetsma, J. S. Killeen, B. L. Feringa, R. Hage, G. Roelfes, W. R. Browne, *Inorg. Chem.* **2012**, *51*, 900-913.

[2] G. Roelfes, M. Lubben, K. Chen, R Y. N. Ho, A. Meetsma, S. Genseberger, R. M. Hermant, R. Hage, S. K. Mandal, V. G. Young, Jr., Y. Zang, H. Kooijman, A. L. Spek, L. Que, Jr., B. L. Feringa, *Inorg. Chem*., **1999**, *38*, 1929-1936.

[3] ASTM E1840-96(2007) Standard Guide for Raman Shift Standards for Spectrometer Calibration, ASTM International, DOI: 10.1520/E1840-96R07.

[4] A. R. McDonald, L. Que, Jr., *Coord. Chem. Rev*. **2013**, *257*, 414-428. And references therein.

[5] The pH of the solution changed from 3.3 to 3.5 and 3.7 for first and second batch additions of 0.5 equiv of NaOCl, respectively.

[6] Due to the instability of $[(N4Py)Fe(CH_3CN)](ClO_4)_2$ in water towards ligand dissociation at pH < 3, experiments were performed at ca. pH 3.

[7] Y. -M. Lee, S. N. Dhuri, S. C. Sawant, J. Cho, M. Kubo, T. Ogura, S. Fukuzumi, W. Nam, *Angew. Chem. Int. Ed.* **2009**, *48*, 1803-1806.

[8] Assignment of the origin of the oxygen in $[(MeN4Py)Fe^{IV}(O)]^{2+}$ is not possible due to the rapid exchange of NaOCl with H_2O.

[9] Aqueous NaOBr was prepared by adding solid NaBr to an aqueous solution of NaOCl. The O-Cl stretching mode in the NaOCl (711 cm^{-1}) was replaced by a new band at 618 cm^{-1} assigned to O-Br stretch after addition of NaBr (Figure S12).

[10] Z. Cong, S. Yanagisawa, T. Kurahashi, T. Ogura, S. Nakashima, H. Fuji, *J. Am. Chem. Soc.* **2012**, *134*, 20617-20620.

[11] (a) T. Petrenko, F. Neese, *J. Chem. Phys*., **2007**, *127*, 164319; (b) T. Petrenko, F. Neese, *J. Chem. Phys*., **2012**, *137*, 234107

[12] A. D. Becke, *Phys. Rev. A*, **1988,** *38*, 3098.

[13] J. P. Perdew, *Phys. Rev. B*, **1986,** *33***,** 8822.

[14] (a) A. Schaefer, H. Horn and R. Ahlrichs, *J. Chem. Phys*. **1992**, *97*, 2571; (b) F. Weigenda and R. Ahlrichs, *Phys. Chem. Chem. Phys*., **2005**, *7*, 3297.

[15] (a) F. Negri and M. Z. Zgierski, *J. Chem. Phys*. 1994, *100*, 1387; (b) K. Gustav and M. Storch, *Int. J. Quantum Chem.* **1990**, *38*, 25; (c) K. Gustav, C. Seydenschwanz, *Chem. Phys. Lett*. **1986**, *123*, 261.

[16] A. Schaefer, C. Huber, R. Ahlrichs, *J. Chem. Phys.* **1994**, *100*, 5829.

[17] (a) A. D. Becke, *J. Chem. Phys*. **1993**, *98*, 5648–5652; (b) C. Lee, W. Yang, R. G. Parr, *Phys. Rev. B* **1988**, *37*, 785–789.

[18] *Gaussian*-09, Revision B.01, M. J. Frisch, G. W. Trucks, H. B. Schlegel, G. E. Scuseria, M. A. Robb, J. R. Cheeseman, G. Scalmani, V. Barone, B. Mennucci, G. A. Petersson, H. Nakatsuji, M. Caricato, X. Li, H. P. Hratchian, A. F. Izmaylov, J. Bloino, G. Zheng, J. L. Sonnenberg, M. Hada, M. Ehara, K. Toyota, R. Fukuda, J. Hasegawa, M. Ishida, T. Nakajima, Y. Honda, O. Kitao, H. Nakai, T. Vreven, J. A. Montgomery, Jr., J. E. Peralta, F. Ogliaro, M. Bearpark, J. J. Heyd, E. Brothers, K. N. Kudin, V. N. Staroverov, T. Keith, R. Kobayashi, J. Normand, K. Raghavachari, A. Rendell, J. C. Burant, S. S. Iyengar, J. Tomasi, M. Cossi, N. Rega, J. M. Millam, M. Klene, J. E. Knox, J. B. Cross, V. Bakken, C. Adamo, J. Jaramillo, R. Gomperts, R. E. Stratmann, O. Yazyev, A. J. Austin, R. Cammi, C. Pomelli, J. W. Ochterski, R. L. Martin, K. Morokuma, V. G. Zakrzewski, G. A. Voth, P. Salvador, J. J. Dannenberg, S. Dapprich, A. D. Daniels, O. Farkas, J. B. Foresman, J. V. Ortiz, J. Cioslowski, and D. J. Fox, Gaussian, Inc., Wallingford CT, **2010**.

[19] S. Grimme, J. Antony, S. Ehrlich, H.Krieg, *J. Chem. Phys*. **2010**, *132*, 154104-154119.

[20] *Jaguar* 7.7, version 7.9, Schrodinger, LLC, New York, NY, **2011**.

[21] P. J. Hay, W. R. Wadt, *J. Chem. Phys*. **1985**, *82*, 299–310.
